# Minimality and comparison of sets of multi-attribute vectors

**DOI:** 10.1007/s10458-022-09572-8

**Published:** 2022-08-13

**Authors:** Federico Toffano, Nic Wilson

**Affiliations:** Insight Centre for Data Analytics, School of Computer Science and Information Technology, University College Cork, Cork, Ireland

**Keywords:** Possibly strictly optimal alternatives, Multi-criteria decision making, Multi-criteria utility theory, Multi-objective decision support systems, Preference elicitation, MSC Code1, MSC Code2, More

## Abstract

In a decision-making problem, there is often some uncertainty regarding the user preferences. We assume a parameterised utility model, where in each scenario we have a utility function over alternatives, and where each scenario represents a possible user preference model consistent with the input preference information. With a set $A$ of alternatives available to the decision-maker, we can consider the associated utility function, expressing, for each scenario, the maximum utility among the alternatives. We consider two main problems: firstly, finding a minimal subset of $A$ that is equivalent to it, i.e., that has the same utility function. We show that for important classes of preference models, the set of possibly strictly optimal alternatives is the unique minimal equivalent subset. Secondly, we consider how to compare $A$ to another set of alternatives $B$, where $A$ and $B$ correspond to different initial decision choices. This is closely related to the problem of computing setwise max regret. We derive mathematical results that allow different computational techniques for these problems, using linear programming, and especially, with a novel approach using the extreme points of the epigraph of the utility function.

## Introduction

In a decision-making problem, there can often be uncertainty regarding the user preferences. Suppose that, in a particular situation, $A$ is the set of alternatives that are available to the decision-maker. This is interpreted in a disjunctive fashion, in that the user is free to choose any element $\alpha$ of $A$. However, as is common, we do not know precisely the user’s preferences. The preference information available to the system is represented in terms of a set of user preference models, parameterised by a set (of *scenarios*) ${\mathcal W}$ where, associated with each scenario $w\in {\mathcal W}$, is a (real-valued) utility function $f_w$ over alternatives.

Each element $w$ of ${\mathcal W}$ is viewed as representing a possible model of the user’s preferences that is consistent with the preference information we know. If we knew that $w$ were the true scenario, so that $f_w$ represents the user’s preferences over alternatives, then we would be able to choose a best element of $A$ with respect to $f_w$, leading to a utility value $\textit{Ut}_A(w) = \max _{\alpha \in A} f_w(\alpha )$. However, the situation will frequently be ambiguous given a non-singleton set ${\mathcal W}$ of possible user models or scenarios.

The set ${\mathcal W}$ incorporates what we know about the user preferences; for example, if we have learned that the user regards alternative $\beta$ as at least as good as alternative $\gamma$, then ${\mathcal W}$ will only include scenarios $w$ such that $f_w(\beta ) \ge f_w(\gamma )$.

This framework is fairly general; for instance, the utility function $f_w$ may be based on a decomposition of utility, using, for example, an additive representation for a combinatorial problem (e.g., [37, 42, 58, 62]). Also, $f_w(\alpha )$ could represent the expected utility of alternative $\alpha$ given that $w$ is the correct user model, based on a probabilistic model with parameter $w$, for example in a multi-objective influence diagram [22, 41, 43], with $\alpha$ corresponding to a policy.

We consider, in particular, the following related pair of questions:

1. Are there elements of $A$ that can be eliminated unproblematically? In particular, is there a strict subset $A'$ of $A$ that is equivalent to $A$?
2. Given a choice between one situation, in which the available alternatives are $A$, and another situation, in which alternatives $B$ are available, is $A$ at least as good as $B$ in every scenario?

Regarding (1), we need to be able to eliminate unimportant choices, which can help to make the list of options manageable, in particular, if we want to display the alternatives to the user. We interpret this as finding a minimal subset $A'$ of $A$ such that $\textit{Ut}_A(w) = \textit{Ut}_{A'}(w)$ for every scenario $w\in {\mathcal W}$. In this situation, we say that $A$ and $A'$ are *utility-equivalent*.

Question (2) concerns a case in which the user may have a choice between (I) being able to obtain any of the set of alternatives $A$, and (II) any alternative in $B$ (and thus, the user could obtain any alternative in $A\cup B$). Sets $A$ and $B$ may correspond to different choices $X=a$ and $X=b$ of a fundamental variable *X*, and determining that $A$ dominates $B$ may lead us to exclude $X=b$, thus simplifying the problem. For instance, $A$ might correspond to hotels in Paris, and $B$ to hotels in Lisbon, for a potential weekend away. It can be useful to determine if one of these clearly dominates the other; if, for instance, $A$ dominates $B$, then there may be no need for the system and the user to further consider $B$, and may therefore focus on Paris rather than Lisbon. We interpret this task as determining if in every scenario the utility of $A$ is at least that for $B$, i.e., $\textit{Ut}_A(w) \ge \textit{Ut}_B(w)$ for all $w\in {\mathcal W}$. We can this relation *utility-dominance*.

The focus of this paper is to determine important properties of the utility-dominance and utility-equivalence relations, and to derive computational procedures, in order to find a minimal equivalent subset, and for testing dominance between $A$ and $B$; we also determine properties and a computational technique for a form of maximum regret, that can be viewed as a degree of dominance, and which corresponds to *setwise max regret* defined in [62, 65], and relates to the value of a query. The main computational procedures are based on linear programming (LP), or, alternatively, a novel method using the extreme points of the epigraph of the utility function (which we abbreviate to EEU). These procedures have been compared and evaluated in [59].

From the computational perspective we focus especially on the case in which each alternative $\alpha$ is associated with a multi-attribute utility vector $\hat{\alpha }$, based on a weighted average user preference model. Each utility vector is then an element of ${I\!R}^{p}$, representing a number ${p}$ of scales of utility (or objectives); each scenario $w$ is a (typically normalised non-negative) vector in ${I\!R}^{p}$, with $\alpha \succcurlyeq _w\beta$ if and only if the weighted sum of $\hat{\alpha }$ with respect to $w$ is at least that of $\hat{\beta }$. An input preference of $\alpha$ over $\beta$ then leads to a linear constraint on the weights vector $w$, and we can define the set of consistent preference models ${\mathcal W}$ as the convex polytope generated by a set of input preferences of this form.

Given a finite set of alternatives $A$, we define the notion of setwise-minimal equivalent subset, and we show relationships with the set $\mathrm{PSO}_{\mathcal W}(A)$ of possibly strictly optimal elements, where ${\mathcal W}$ is the set of parameters relating to a family of user preference models. It suffices to consider sets $A$ of alternatives that are equivalence-free, i.e., such that no two alternatives in $A$ have identical utility in all scenarios. For equivalence-free $A$, the set $\mathrm{PSO}_{\mathcal W}(A)$ consists of those alternatives $\alpha \in A$ that are such that there exists some scenario in ${\mathcal W}$ for which $\alpha$ is the only optimal alternative in $A$.

The main contributions of the paper are as follows:

- we give sufficient conditions for the set $\mathrm{PSO}_{\mathcal W}(A)$ of possibly strictly optimal alternatives to be utility-equivalent with $A$. These apply for many natural situations including when the user preferences models are linear, and the set ${\mathcal W}$ of parameters defining the set of user preference models is convex.
- We show that then $\mathrm{PSO}_{\mathcal W}(A)$ is the unique minimal equivalent subset of $A$, for equivalence-free $A$ (i.e., when no two alternatives in $A$ have identical utility in all scenarios in ${\mathcal W}$). Furthermore, the $\mathrm{PSO}_{\mathcal W}$ operator can be used to filter query sets to avoid the potential of a partially inconsistent answer.
- We derive sophisticated computational methods for computing utility dominance, setwise max regret and for computing the unique minimal equivalent subset. These include both linear programming methods, and extreme point methods using the epigraph of the utility functions. To increase the efficiency, a number of pre-processing methods are developed, using the properties we derive for dominance relations between sets. Our algorithms are experimentally tested based on randomly generated instances.

This paper extends an earlier work [60], including further theoretical results with proofs of theorems, propositions and lemmas, and new experimental results related to a new implementation of our algorithms fixing some runtime exceptions related to precision issues (see Sect. 13). Part of this work is also included in the first author’s PhD thesis [56].

Section 2 discusses related work. Section 3 gives the formal setup, defining dominance relations between sets of alternatives, and giving basic properties. Section 4 discusses different ways of defining optimal alternatives in a set, including the possibly strictly optimal set. Section 5 defines the setwise minimal equivalent set, for a set of alternatives, and explores the relationship between such sets and the possibly strictly optimal set. Section 6 considers continuous spaces of scenarios, and gives a sufficient condition for equivalence of the possibly strictly optimal set with the input set of alternatives. Section 7 considers the problem of reducing the size of a set $A$, whilst maintaining equivalence. Section 8 defines a form of maximum regret in this context, shows how it relates to dominance, and gives properties that will be useful for computation. Section 9 discusses the importance of the possibly optimal and possibly strictly optimal alternatives in incremental preference elicitation. Section 10 describes the EEU method. Section 11 brings together the computational techniques for the weighted multi-attribute utility case. Sections 12 and 13 describe the implementation and experimental testing, and Sect. 14 concludes the paper.

## Related work

Multiattribute utility theory (MAUT) [37] involves numerical representations of user preferences with respect to alternatives evaluated over multiattribute spaces. Imprecisely specified multiattribute utility theory (ISMAUT) [68] is one of the earliest attempt to deal with parameterised utility information representing user preferences with linear inequalities and reducing the set of alternatives to those that are not dominated by any other alternative. Related research such as [33] and [67], deals with similar issues.

A major division in recent work on parameterised user preference models is whether a Bayesian model is assumed over the scenarios (corresponding to the different user preference models), or if there is a purely qualitative (logical) representation of the uncertainty over scenarios, where all we represent is that the scenario is in a set ${\mathcal W}$. Bayesian approaches include [12, 13, 20, 64]. Work involving a qualitative uncertainty representation includes [7, 14, 18, 42, 62]. Linear imprecise preference models, including those based on a simple form of MAUT model, have been considered in work such as [18, 36, 41, 49] including in a conversational recommender system context [19, 62, 63].

Parameterised preference models are commonly used with interactive preference elicitation approaches (see, e.g., [9, 30, 38, 54, 57]). In this context, the purpose is to explore the alternatives based on different interactions with the decision-maker and without listing all the available alternatives. The parameterisation represents different decision-maker’s preference scenarios, and the likelihood or the restrictions on the parameters represent the information obtained by the interaction with the decision-maker. Methods that iteratively interact with the decision-maker to reduce the uncertainty about a parameterised preference models are also called *Incremental* [3, 10, 40]. In general, the purpose of such methods is to recommend alternatives to the decision-maker without defining a precise utility function using methods such as the minimax regret criterion (see Sect. 8). A typical interactive approach consists of the following steps:

1. *Computation*: generate some undominated solutions.
2. *Interaction*: show to the decision-maker some of the generated solutions asking to input new preference information.
3. *Termination*: interruption of the elicitation process by the decision-maker or if some specified stopping criterion has been satisfied.

Different approaches have been explored to generate new queries for the decision-maker (see, e.g., [52]). A classical interactive approach is based on a comparison of alternatives (see, e.g., [53, 75]), i.e., the preference elicitation system asks the decision-maker to specify their preference between a set of alternatives, and the response is used to reduce the uncertainty of the preference model. Ideally, the uncertainty of the preference model should be reduced to a scenario in which we have a unique optimal alternative according to the preference information collected. However, often this is unfeasible or requires too many interactions with the decision-maker. Because of this, the parameterisation of utility functions leads to different notions of optimality that can be used to classify alternatives (see, e.g., [71]).

In Sect. 4 we consider a number of operators representing different notions of optimality. The set $\mathrm{UD}_{\mathcal W}(A)$ is a subset of $A$ not including strictly dominated alternatives, a natural generalisation of the Pareto-optimal elements, appears in many contexts, e.g., [36, 42]. Possibly optimal $\mathrm{PO}_{\mathcal W}(A)$ (also known as *potentially optimal*) elements have been considered in many publications, such as [6, 10, 28, 29, 33, 72]. See Sect. 11 for details and references about the computation of $\mathrm{UD}_{\mathcal W}(A)$ and $\mathrm{PO}_{\mathcal W}(A)$ for linear and convex utility functions. The possibly strictly optimal set $\mathrm{PSO}_{\mathcal W}(A)$ and the maximally possibly optimal set $\mathrm{MPO}_{\mathcal W}(A)$ have been considered much less [46, 70, 71]. Regret-based decision making has a long history, with recent work in AI including [7, 14, 18]. We describe in Sect. 8 the relationship between the dominance relation $\succcurlyeq _{\forall \forall \exists }^{{\mathcal W}}$ and setwise max regret [62, 65]. See Sect. 11 for details and references about the computation of the setwise max regret and the setwise minimax regret for linear and convex utility functions.

## Basic terminology and dominance relations between sets

In this section we give some basic definitions, in particular regarding utility-dominance and related dominance relations, and utility-equivalence. Section 3.1 describes the basic set up, involving a parameterised family of utility functions over a set $\Omega$ of alternatives. There are natural dominance and equivalence relations induced between alternatives, as described in Sect. 3.2. The utility of a finite subset of alternatives is defined to be the maximum utility over the alternatives in the set. This leads to a natural (utility-)dominance relation between finite sets of alternatives, and the corresponding utility-equivalence relation, as described in Sect. 3.3, where $A$ utility-dominates $B$ if and only if in every compatible scenario, the utility of $A$ is at least as great as the utility of $B$. We also define two computationally simpler dominance relations between sets, which are useful as sufficient conditions for utility-dominance.

### Uncertain preference structures

We consider a (possibly infinite) set $\Omega$ of alternatives, and another set ${\mathcal U}$, the elements of which we call *scenarios*, that corresponds with a set of user preference models. With each scenario $w\in {\mathcal U}$ is associated a utility function $f_w$ on $\Omega$, i.e., a function from $\Omega$ to ${I\!R}$; this gives rise to a total pre-order $\succcurlyeq _w$ on $\Omega$ given by $\alpha \succcurlyeq _w\beta$ $\iff$ $f_w(\alpha ) \ge f_w(\beta )$, for $\alpha ,\beta \in \Omega$.

For each $w\in {\mathcal U}$, we also define associated relations $\succ _w$ and $\equiv _w$ in the standard way: $\alpha \succ _w\beta$ if and only if $\alpha \succcurlyeq _w\beta$ and $\lnot ( \beta \succcurlyeq _w\alpha )$, which is if and only if $f_w(\alpha ) > f_w(\beta )$. We define $\alpha \equiv _w\beta$ if and only if $\alpha \succcurlyeq _w\beta$ and $\beta \succcurlyeq _w\alpha$, which is if and only if $f_w(\alpha ) = f_w(\beta )$.

*Notation* ${\mathcal M}$: We use the symbol ${\mathcal M}$ to represent the set of finite non-empty subsets of the set of alternatives $\Omega$.

Each $A\in {\mathcal M}$ gives rise to a function giving values of utility for each element of ${\mathcal U}$. This expresses how good the set $A$ is, with respect to different user models.

#### Definition 1

(*Utilities associated with* $A$.) We define, for $w\in {\mathcal U}$, $\textit{Ut}_A(w)$ to be $\max _{\alpha \in A} f_w(\alpha )$.

We do not *a priori* assume anything about the functions $f_w$; however certain mathematical results make additional assumptions, such as continuity with respect to $w$. Of particular interest in this paper is the case when $f_w(\alpha )$ is a linear function of $w$, where ${\mathcal U}= {I\!R}^{p}$ for some ${p}$, and so $f_w(\alpha )$ can be written as $\sum _{i=1}^{p}\alpha _i w(i) = (\alpha _1, \ldots ,\alpha _{{p}})\cdot w$, for some reals $\alpha _i$, with $\alpha _i$ representing how good alternative $\alpha$ is with respect to objective/criterion *i*. We then write the vector $(\alpha _1, \ldots ,\alpha _{{p}})$ as $\hat{\alpha }$. Thus, in the linear case, for each $\alpha \in \Omega$ there exists an associated vector $\hat{\alpha }\in {I\!R}^{p}$, and $f_w(\alpha ) = \hat{\alpha }\cdot w$ for all $w\in {\mathcal U}$.

If $\alpha \in {I\!R}^{p}$ then we could set $\hat{\alpha }= \alpha$, giving a simple weighted sum utility function. However, this linear function case also covers more complex preference models, including GAI representations [16, 31], Ordered Weighted Averages [73], and preference models based on Choquet integrals [7, 32], since the utility functions in these cases are linear in the parameter $w$ (although not linear with respect to $\alpha$ in the latter two cases). For the GAI and Choquet representations, there are a larger number of parameters, so the dimension ${p}$ of the utility and parameter spaces is larger.

### Dominance and equivalence between alternatives

Very often we will have information that restricts the set of scenarios (i.e., user models). In particular, the user may previously have answered some queries, and we then only consider the set ${\mathcal W}$ of user models compatible with their answers.

For ${\mathcal W}\subseteq {\mathcal U}$ we define relation $\succcurlyeq _{\mathcal W}$ on $\Omega$ by $\alpha \succcurlyeq _{\mathcal W}\beta$ $\iff$ for all $w\in {\mathcal W}$, $\alpha \succcurlyeq _w\beta$. Thus, $\alpha \succcurlyeq _{\mathcal W}\beta$ holds if and only if $\alpha$ is at least as good as $\beta$ in every scenario in ${\mathcal W}$. We define $\succ _{\mathcal W}$ to be the strict part of $\succcurlyeq _{\mathcal W}$, i.e., for $\alpha ,\beta \in \Omega$, $\alpha \succ _{\mathcal W}\beta$ if and only if $\alpha \succcurlyeq _{\mathcal W}\beta$ and $\beta \not \succcurlyeq _{\mathcal W}\alpha$. Thus, $\alpha \succ _{\mathcal W}\beta$ if and only if $\alpha$ is at least as good as $\beta$ in every scenario in ${\mathcal W}$, and strictly better in at least one scenario in ${\mathcal W}$. Relation $\succ _{\mathcal W}$ is transitive and acyclic. We define equivalence relation $\equiv _{\mathcal W}$ to be the symmetric part of $\succcurlyeq _{\mathcal W}$, given by $\alpha \equiv _{\mathcal W}\beta$ if and only if $\alpha \succcurlyeq _{\mathcal W}\beta$ and $\beta \succcurlyeq _{\mathcal W}\alpha$.

We define the notion of being *equivalence-free*. Alternatives $\alpha$ and $\beta$ are equivalent, i.e., $\alpha \equiv _{\mathcal W}\beta$, if and only if $\alpha \equiv _w\beta$ for all $w\in {\mathcal W}$. It can be unnecessary to include two equivalent alternatives $\alpha$ and $\beta$ in a set of alternatives $A$, since $\alpha$ should be acceptable if and only if $\beta$ is acceptable. In an *equivalence-free* set of alternatives no two alternatives are equivalent:

#### Definition 2

(*Equivalence-free*) We say that $A$ ($\in {\mathcal M}$) is $\equiv _{\mathcal W}$-free (or *equivalence-free*) if for all $\alpha ,\beta \in A$, we have $\alpha \not \equiv _{\mathcal W}\beta$.

One can reduce any $A$ to an equivalence-free set $A'$ by including exactly one element in $A'$ of each $\equiv _{\mathcal W}$-equivalence class in $A$.

### Dominance relations between sets

Given a set of scenarios ${\mathcal W}$, we will define three relations, $\succcurlyeq _{\forall \forall \exists }^{{\mathcal W}}$, $\succcurlyeq _{\forall \exists \forall }^{{\mathcal W}}$ and $\succcurlyeq _{\exists \forall \forall }^{{\mathcal W}}$, between sets of alternatives $A$ and $B$ in ${\mathcal M}$, that specify when $A$ is better than $B$. These relations are based on a set ${\mathcal W}$ of scenarios, and the corresponding set of relations, $\succcurlyeq _w$, for $w\in {\mathcal W}$. In this paper we focus especially on the relation $\succcurlyeq _{\forall \forall \exists }^{{\mathcal W}}$, with the other two relations being useful computationally. $A\succcurlyeq _{\forall \forall \exists }^{{\mathcal W}} B$ holds if and only if in every scenario $w\in {\mathcal W}$, there’s an element of $A$ that is at least as good as any element of $B$. The relation thus relates to Question (2) in the introduction. This section gives some basic properties of these three dominance relations between sets, that are useful for our algorithmic methods.

#### Definition 3

(*Dominance relations* $\succcurlyeq _{\forall \forall \exists }^{{\mathcal W}}$, $\succcurlyeq _{\forall \exists \forall }^{{\mathcal W}}$ and $\succcurlyeq _{\exists \forall \forall }^{{\mathcal W}}$) For subset ${\mathcal W}$ of ${\mathcal U}$, we define binary relations $\succcurlyeq _{\forall \forall \exists }^{{\mathcal W}}$, and $\succcurlyeq _{\forall \exists \forall }^{{\mathcal W}}$ and $\succcurlyeq _{\exists \forall \forall }^{{\mathcal W}}$ on ${\mathcal M}$ as follows. Consider any $A, B\in {\mathcal M}$.

- $A\succcurlyeq _{\forall \forall \exists }^{{\mathcal W}} B$ holds if and only if for all $\beta \in B$ and for all $w\in {\mathcal W}$ there exists $\alpha \in A$ such that $\alpha \succcurlyeq _w\beta$.
- $A\succcurlyeq _{\forall \exists \forall }^{{\mathcal W}} B$ holds if and only if for all $\beta \in B$ there exists $\alpha \in A$ such that for all $w\in {\mathcal W}$, $\alpha \succcurlyeq _w\beta$.
- $A\succcurlyeq _{\exists \forall \forall }^{{\mathcal W}} B$ holds if and only if there exists $\alpha \in A$ such that for all $\beta \in B$ and for all $w\in {\mathcal W}$, $\alpha \succcurlyeq _w\beta$.

In the notation in the subscript for the three relations (such as $\forall \exists \forall$ in relation $\succcurlyeq _{\forall \exists \forall }^{{\mathcal W}}$), the first $\forall$ symbol relates to the quantification *for all* $\beta \in B$, the second $\forall$ symbol relates to *for all* $w\in {\mathcal W}$, and the $\exists$ symbol relates to *there exists* $\alpha \in A$.

Relation $\succcurlyeq _{\forall \forall \exists }^{{\mathcal W}}$ and its corresponding equivalence relation are the main foci of attention in this paper. However, for computational reasons we consider relations $\succcurlyeq _{\forall \exists \forall }^{{\mathcal W}}$ and $\succcurlyeq _{\exists \forall \forall }^{{\mathcal W}}$, which allow computationally efficient sufficient conditions for $A\succcurlyeq _{\forall \forall \exists }^{{\mathcal W}} B$.

Since each relation $\succcurlyeq _w$ is a total pre-order, and $A$ is finite (as is $B$), we have $A\succcurlyeq _{\forall \forall \exists }^{{\mathcal W}} B$ if and only if for all $w\in {\mathcal W}$ there exists $\alpha \in A$ such that for all $\beta \in B$, $\alpha \succcurlyeq _w\beta$.

Relations $\succcurlyeq _{\forall \exists \forall }^{{\mathcal W}}$ and $\succcurlyeq _{\exists \forall \forall }^{{\mathcal W}}$ can be written in terms of $\succcurlyeq _{\mathcal W}$:

- $A\succcurlyeq _{\forall \exists \forall }^{{\mathcal W}} B$ holds if and only if for all $\beta \in B$ there exists $\alpha \in A$ such that $\alpha \succcurlyeq _{\mathcal W}\beta$, i.e., every element of $B$ is $\succcurlyeq _{\mathcal W}$-dominated by some element of $A$.
- $A\succcurlyeq _{\exists \forall \forall }^{{\mathcal W}} B$ holds if and only if there exists $\alpha \in A$ such that for all $\beta \in B$, $\alpha \succcurlyeq _{\mathcal W}\beta$, i.e., there’s some element $\alpha$ of $A$ that is better than every element of $B$ in every scenario.

The three relations are all transitive, and are nested: $\succcurlyeq _{\exists \forall \forall }^{{\mathcal W}}$ $\subseteq$ $\succcurlyeq _{\forall \exists \forall }^{{\mathcal W}}$ $\subseteq$ $\succcurlyeq _{\forall \forall \exists }^{{\mathcal W}}$. Thus, for example, $A\succcurlyeq _{\forall \exists \forall }^{{\mathcal W}} B$ implies $A\succcurlyeq _{\forall \forall \exists }^{{\mathcal W}} B$. We also have chaining properties for the three relations. These are valuable, for instance, if we are comparing a number of sets $A_i$, $i=1,\ldots , K$, since if we determine that $A_i \succcurlyeq _{\forall \forall \exists }^{{\mathcal W}} A_j$ and $A_j\succcurlyeq _{\forall \forall \exists }^{{\mathcal W}} A_k$, then we do not need to check that $A_i \succcurlyeq _{\forall \forall \exists }^{{\mathcal W}} A_k$, since it is implied.

#### Proposition 1

For any ${\mathcal W}\subseteq {\mathcal U}$ we have $\succcurlyeq _{\exists \forall \forall }^{{\mathcal W}}$ $\subseteq$ $\succcurlyeq _{\forall \exists \forall }^{{\mathcal W}}$ $\subseteq$ $\succcurlyeq _{\forall \forall \exists }^{{\mathcal W}}$. Also, $\succcurlyeq _{\mathcal W}$ and each of the relations, $\succcurlyeq _{\forall \forall \exists }^{{\mathcal W}}$,

$\succcurlyeq _{\forall \exists \forall }^{{\mathcal W}}$ and $\succcurlyeq _{\exists \forall \forall }^{{\mathcal W}}$, is transitive. Furthermore, we have the following chaining properties:

(i) If $A\succcurlyeq _{\forall \exists \forall }^{{\mathcal W}} B$ and $B\succcurlyeq _{\exists \forall \forall }^{{\mathcal W}} C$ then $A\succcurlyeq _{\exists \forall \forall }^{{\mathcal W}} C$.
(ii) If $\succcurlyeq _{*}^{{\mathcal W}}$ is any of the relations $\succcurlyeq _{\forall \forall \exists }^{{\mathcal W}}$, $\succcurlyeq _{\forall \exists \forall }^{{\mathcal W}}$ or $\succcurlyeq _{\exists \forall \forall }^{{\mathcal W}}$ then $A\succcurlyeq _{\exists \forall \forall }^{{\mathcal W}} B$ and $B\succcurlyeq _{*}^{{\mathcal W}} C$ implies $A\succcurlyeq _{\exists \forall \forall }^{{\mathcal W}} C$.

*Corresponding equivalence relations:* As well as being transitive, relations $\succcurlyeq _{\forall \forall \exists }^{{\mathcal W}}$ and $\succcurlyeq _{\forall \exists \forall }^{{\mathcal W}}$ are reflexive (relation $\succcurlyeq$ on ${\mathcal M}$ is *reflexive* if for all $A\subseteq \Omega$, $A\succcurlyeq A$). Because the relation $\succcurlyeq _{\forall \forall \exists }^{{\mathcal W}}$ is reflexive and transitive, its symmetric part, defined by $A\equiv _{\forall \forall \exists }^{{\mathcal W}} B$ if and only if $A\succcurlyeq _{\forall \forall \exists }^{{\mathcal W}} B$ and $B\succcurlyeq _{\forall \forall \exists }^{{\mathcal W}} A$, is an equivalence relation. Similarly, we define the relation $\equiv _{\forall \exists \forall }^{{\mathcal W}}$ to be the symmetric part of $\succcurlyeq _{\forall \exists \forall }^{{\mathcal W}}$ with $A\equiv _{\forall \exists \forall }^{{\mathcal W}} B$ if and only if $A\succcurlyeq _{\forall \exists \forall }^{{\mathcal W}} B$ and $B\succcurlyeq _{\forall \exists \forall }^{{\mathcal W}} A$. Therefore we have $A\equiv _{\forall \exists \forall }^{{\mathcal W}} B$ if and only if (i) for all $\beta \in B$ there exists $\alpha \in A$ such that $\alpha \succcurlyeq _{\mathcal W}\beta$; and (ii) for all $\alpha \in A$ there exists $\beta \in B$ such that $\alpha \succcurlyeq _{\mathcal W}\beta$. Proposition 1 implies that $\equiv _{\forall \exists \forall }^{{\mathcal W}}$ $\subseteq$ $\equiv _{\forall \forall \exists }^{{\mathcal W}}$.

Clearly, $\succcurlyeq _{\forall \forall \exists }^{{\mathcal W}}$ and $\succcurlyeq _{\forall \exists \forall }^{{\mathcal W}}$ determine the corresponding equivalence relations; conversely, $\succcurlyeq _{\forall \forall \exists }^{{\mathcal W}}$ and $\succcurlyeq _{\forall \exists \forall }^{{\mathcal W}}$ can be expressed in terms of their corresponding equivalence relations:

#### Proposition 2

For all $A,B\in {\mathcal M}$, $A\succcurlyeq _{\forall \forall \exists }^{{\mathcal W}} B$ $\iff$ $A\equiv _{\forall \forall \exists }^{{\mathcal W}} A\cup B$; and $A\succcurlyeq _{\forall \exists \forall }^{{\mathcal W}} B$ $\iff$ $A\equiv _{\forall \exists \forall }^{{\mathcal W}} A\cup B$.

The following result shows how the relation $\succcurlyeq _{\forall \forall \exists }^{{\mathcal W}}$ and its corresponding equivalence relation $\equiv _{\forall \forall \exists }^{{\mathcal W}}$ can be expressed in terms of the utility functions. For this reason, we refer to the relation $\equiv _{\forall \forall \exists }^{{\mathcal W}}$ as *utility-equivalence*, and the relation $\succcurlyeq _{\forall \forall \exists }^{{\mathcal W}}$ as *utility-dominance*. Part (iii) gives another representation of the relation $\succcurlyeq _{\exists \forall \forall }^{{\mathcal W}}$ that allows efficient computation.

#### Proposition 3

Consider any ${\mathcal W}\subseteq {\mathcal U}$ and $A, B\in {\mathcal M}$.

(i) $$A\succcurlyeq _{\forall \forall \exists }^{{\mathcal W}} B$$ $$\iff $$ for all $$w\in {\mathcal W}$$, $$\textit{Ut}_A(w) \ge \textit{Ut}_B(w)$$.
(ii) $A\equiv _{\forall \forall \exists }^{{\mathcal W}} B$ $\iff$ for all $w\in {\mathcal W}$, $\textit{Ut}_A(w) = \textit{Ut}_B(w)$.
(iii) $A\succcurlyeq _{\exists \forall \forall }^{{\mathcal W}} B$ if and only if there exists $\alpha \in A$ such that for all $w\in {\mathcal W}$, $f_w(\alpha ) \ge \textit{Ut}_B(w)$.

One can also consider a (strong form of) strict dominance $A\gg _{\forall \forall \exists }^{{\mathcal W}} B$ defined as for all $w\in {\mathcal W}$, $\textit{Ut}_A(w) > \textit{Ut}_B(w)$; this corresponds with the dominance relation defined in Definition 2 of [10].

#### Example 1

**Fig. 1:**
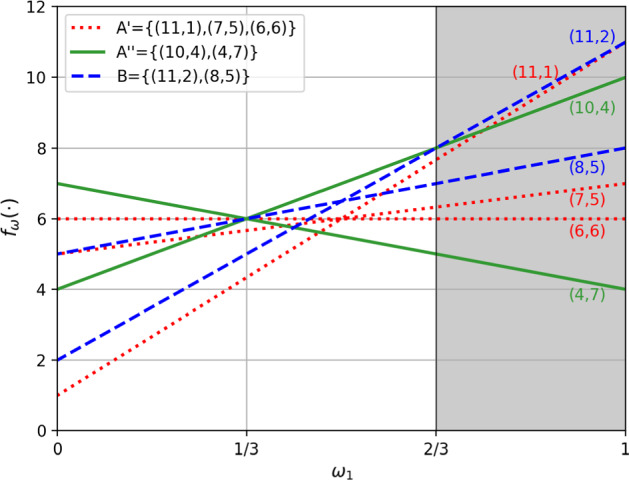
$f_w(\alpha )$ and $f_w(\beta )$ for each $\alpha \in A$ and $\beta \in B$, where $A= A'\cup A'' = {\{ (11,1), (10, 4), (7,5), (6,6), (4,7) \}}$, $B= {\{(11,2), (8,5)\}}$ and $w\in {\mathcal W}={\{(w_1, w_2) \, : \,w_1 + w_2 = 1 \  \&  \ 0\le w_1 \le \frac{2}{3} \}}$

Let $A= {\{ (11,1), (10, 4), (7,5), (6,6), (4,7)\}}$ and $B= {\{(11,2), (8,5)\}}$ be sets of utility vectors of hotels in Paris and Lisbon respectively. For example, the first value of each utility vector could be a score for the location and the second value could be a score for cleanliness, where the higher the score, the better. We assume linear utility functions of the form $f_w(\alpha ) = w\cdot \alpha$. Let ${\mathcal U}= {\{(w_1, w_2) \, : \,w_1, w_2\ge 0 \  \&  \ w_1 + w_2 = 1\}}$, representing different normalised weightings of the two criteria. We assume that the user has an associated weights vector that is unknown and we want to recommend to the user a trip to Paris or Lisbon based on her preferences on the available hotels. Suppose then that we ask the user for her preference between the hotel with utility vector (10, 4) and the hotel with utility vector (11, 2). An input preference of (10, 4) over (11, 2) implies $w\cdot (10,4) \ge w\cdot (11,2)$ and so $2 w_2 \ge w_1$ and thus, $w_1 \le \frac{2}{3}$, leading to the set of scenarios ${\mathcal W}= {\{(w_1, w_2) \, : \,w_1 + w_2 = 1 \  \&  \ 0\le w_1 \le \frac{2}{3} \}}$. This example is illustrated in Fig. 1, and it is easy to see that $A\succcurlyeq _{\forall \forall \exists }^{{\mathcal W}} B$ since for $0\le w_1\le \frac{1}{3}$ there is no line above the line associated to $(4,7)\in A$, and for $\frac{1}{3}\le w_1\le \frac{2}{3}$ there is no line above the line associated to $(10,4)\in A$, i.e., $\not \exists \beta \in B$ s.t. $f_w(\beta ) >\textit{Ut}_A(w)$ for any $w\in {\mathcal W}$. Therefore in this case we can recommend to the user the trip to Paris.

Note that, $A\not \succcurlyeq _{\forall \exists \forall }^{{\mathcal W}} B$ since the line associated to $(8,5)\in B$ is above the line associated to any $\alpha \in A$ in at least one point $w\in {\mathcal W}$.

All three relations satisfy obvious monotonicity properties with respect to $A$, $B$ and ${\mathcal W}$: see Proposition 4 below. In addition, for $\succcurlyeq$ being either $\succcurlyeq _{\forall \forall \exists }^{{\mathcal W}}$ or $\succcurlyeq _{\forall \exists \forall }^{{\mathcal W}}$, to determine if $A\succcurlyeq B$ it is sufficient to check that $A\succcurlyeq {\{\beta \}}$ holds for each $\beta \in B$. We call this property *Right Decomposition*:

#### Definition 4

(*Right Decomposition*) We say that relation $\succcurlyeq$ on ${\mathcal M}$ satisfies the Right Decomposition property if $A\succcurlyeq B$ if and only if $A\succcurlyeq {\{\beta \}}$ holds for each $\beta \in B$.

Relations $\succcurlyeq _{\forall \forall \exists }^{{\mathcal W}}$ and $\succcurlyeq _{\forall \exists \forall }^{{\mathcal W}}$ satisfy the Right Decomposition property; it turns out that this can be useful computationally, as it means that, for $\succcurlyeq$ being either $\succcurlyeq _{\forall \forall \exists }^{{\mathcal W}}$ or $\succcurlyeq _{\forall \exists \forall }^{{\mathcal W}}$, to determine if $A\succcurlyeq B$ it is sufficient to check that $A\succcurlyeq {\{\beta \}}$ holds for each $\beta \in B$.

#### Proposition 4

For any ${\mathcal W}\subseteq {\mathcal U}$. Let $\succcurlyeq ^{\mathcal W}$ be any of $\succcurlyeq _{\forall \forall \exists }^{{\mathcal W}}$, $\succcurlyeq _{\forall \exists \forall }^{{\mathcal W}}$ and $\succcurlyeq _{\exists \forall \forall }^{{\mathcal W}}$, and let $A', B\in {\mathcal M}$ and let $A\subseteq A'$ and let $B'\subseteq B$, and let ${\mathcal W}' \subseteq {\mathcal W}$. If $A\succcurlyeq ^{\mathcal W}B$ then $A' \succcurlyeq ^{{\mathcal W}'} B'$. Relations $\succcurlyeq _{\forall \forall \exists }^{{\mathcal W}}$ and $\succcurlyeq _{\forall \exists \forall }^{{\mathcal W}}$ satisfy Right Decomposition and are reflexive. If $A\supseteq B$ then $A\succcurlyeq _{\forall \forall \exists }^{{\mathcal W}} B$ and $A\succcurlyeq _{\forall \exists \forall }^{{\mathcal W}} B$.

## Some choice functions associated with the set ${\mathcal W}$ of scenarios

Given a finite set $A$ of alternatives, and a set of scenarios (i.e., user models) ${\mathcal W}$, some alternatives may very well be of less interest than others. It is therefore often desirable to define a reduced, i.e., filtered, set $\mathrm{OP}_{\mathcal W}(A)$ of alternatives, by eliminating elements that are considered to be non-optimal. $\mathrm{OP}_{\mathcal W}$ will be a *choice function*, i.e., it maps a finite set $A$ of alternatives to a subset of $A$. There are a variety of the different natural ways of defining such a choice function $\mathrm{OP}_{\mathcal W}$. We consider $\mathrm{UD}_{\mathcal W}(A)$, which removes strictly dominated alternatives from $A$, and $\mathrm{PO}_{\mathcal W}(A)$, which removes alternatives that are not possibly optimal, i.e., not optimal with respect to any scenario in ${\mathcal W}$, and two refined variations, $\mathrm{MPO}_{\mathcal W}(A)$ and $\mathrm{PSO}_{\mathcal W}(A)$. $\mathrm{MPO}_{\mathcal W}(A)$ consists of alternatives in $A$ that are optimal in a maximal set of scenarios.

Our focus is especially on $\mathrm{PSO}_{\mathcal W}(A)$, the set of possibly strictly optimal alternatives; for equivalence-free $A$, this is the set of alternatives that are uniquely optimal in some scenario, i.e., they are (strictly) better than any other element of $A$. Our interest in $\mathrm{PSO}_{\mathcal W}(A)$ is particularly because of the close relationship with minimal equivalent subsets (shown in later sections such as Sects. 5 and 6). The operators $\mathrm{PO}_{\mathcal W}$, $\mathrm{UD}_{\mathcal W}$ and $\mathrm{MPO}_{\mathcal W}$ can all be useful in the efficient computation of $\mathrm{PSO}_{\mathcal W}(A)$. This section gives basic properties of these operators, and their relationships with utility-dominance and utility-equivalence. $\mathrm{PO}_{\mathcal W}$, $\mathrm{UD}_{\mathcal W}$ and $\mathrm{MPO}_{\mathcal W}$ all always maintain utility-equivalence, i.e., for any $A\in {\mathcal M}$, $\mathrm{PO}_{\mathcal W}(A)$, $\mathrm{UD}_{\mathcal W}(A)$ and $\mathrm{MPO}_{\mathcal W}(A)$ are all utility-equivalent with $A$. Although $\mathrm{PSO}_{\mathcal W}$ does not maintain utility-equivalence in general, we will later show that for many natural forms of uncertain preference structure, $\mathrm{PSO}_{\mathcal W}$ does maintain utility-equivalence.

The operators $\mathrm{UD}_{\mathcal W}$, $\mathrm{PO}_{\mathcal W}$, $\mathrm{MPO}_{\mathcal W}$ and $\mathrm{PSO}_{\mathcal W}$ defined below, respect the equivalence $\equiv _{\mathcal W}$ in that if $\mathrm{OP}_{\mathcal W}$ is any of these operators and $\alpha ,\beta \in A$ are such that $\alpha \equiv _{\mathcal W}\beta$ then $\mathrm{OP}_{\mathcal W}(A) \ni \alpha$ $\iff$ $\mathrm{OP}_{\mathcal W}(A) \ni \beta$.

We first define the set $\mathrm{UD}_{\mathcal W}(A)$ of undominated alternatives in $A$.

### Definition 5

(The Undominated set $\mathrm{UD}_{\mathcal W}(A)$.) For $A\in {\mathcal M}$ we define $\mathrm{UD}_{\mathcal W}(A)$ to be the set of $\alpha \in A$ such that there does not exist $\gamma \in A$ such that $\gamma \succ _{\mathcal W}\alpha$.

Thus, the element $\alpha$ of $A$ is not in $\mathrm{UD}_{\mathcal W}(A)$ if and only if there exists some $\gamma \in A$ such that $\gamma$ is at least as good as $\alpha$ in every scenario, and strictly better in at least one scenario. The set $\mathrm{UD}_{\mathcal W}(A)$ is a natural generalisation of the Pareto-optimal elements, and is sometimes referred to as the set of *undominated* elements in $A$.

Proposition 5 gives some basic properties of the relationships between function $\mathrm{UD}_{\mathcal W}$ and the three dominance relations; in particular, part (ii) shows that it preserves equivalences between sets of alternatives, and implies that $\mathrm{UD}_{\mathcal W}(A)$ is non-empty for non-empty $A$.

### Proposition 5

Assume that ${\mathcal W}\subseteq {\mathcal U}$ and $A\in {\mathcal M}$. Then, $\mathrm{UD}_{\mathcal W}(A)$ is non-empty and the following hold.

(i) For $B\in {\mathcal M}$ and, for $\succcurlyeq$ being any of $\succcurlyeq _{\forall \forall \exists }^{{\mathcal W}}$, $\succcurlyeq _{\forall \exists \forall }^{{\mathcal W}}$ or $\succcurlyeq _{\exists \forall \forall }^{{\mathcal W}}$, we have $A\succcurlyeq B$ $\iff$ $\mathrm{UD}_{\mathcal W}(A) \succcurlyeq \mathrm{UD}_{\mathcal W}(B)$.
(ii) $\mathrm{UD}_{\mathcal W}(A) \equiv _{\forall \exists \forall }^{{\mathcal W}} A$ and $\mathrm{UD}_{\mathcal W}(A) \equiv _{\forall \forall \exists }^{{\mathcal W}} A$. Hence, if $A$ is non-empty then $\mathrm{UD}_{\mathcal W}(A)$ is also.

We next define the Possibly Optimal Set $\mathrm{PO}_{\mathcal W}(A)$.

### Definition 6

($\mathrm{O}_w(A)$ and Possibly Optimal Set $\mathrm{PO}_{\mathcal W}(A)$.) For each $w\in {\mathcal U}$ and $A\in {\mathcal M}$ we define $\mathrm{O}_w(A)$ to be all elements $\alpha$ of $A$ that are optimal in $A$ in scenario $w$, i.e., such that for all $\beta \in A$, $\alpha \succcurlyeq _w\beta$. For ${\mathcal W}\subseteq {\mathcal U}$ we define $\mathrm{PO}_{\mathcal W}(A)$ to be $\bigcup _{w\in {\mathcal W}} \mathrm{O}_w(A)$, the set of alternatives that are optimal in some scenario, i.e., optimal for some consistent user preference model.

Next we define the possibly strictly optimal Set $\mathrm{PSO}_{\mathcal W}(A)$:

### Definition 7

($\mathrm{SO}_w^{\mathcal W}(A)$ and Possibly Strictly Optimal Set $\mathrm{PSO}_{\mathcal W}(A)$.) We define $\mathrm{SO}_w^{\mathcal W}(A)$ to be all elements $\alpha$ of $A$ such that $\alpha \succ _w\beta$, for all $\beta \in A$ with $\beta \not \equiv _{\mathcal W}\alpha$. These elements $\alpha$ are said to be *strictly optimal in scenario* $w$. We then define $\mathrm{PSO}_{\mathcal W}(A)$, the set of *possibly strictly optimal* elements, to be $\bigcup _{w\in {\mathcal W}} \mathrm{SO}_w^{\mathcal W}(A)$, i.e., all the elements that are strictly optimal in some scenario in ${\mathcal W}$.

For equivalence-free $A\in {\mathcal M}$, the set $\mathrm{PSO}_{\mathcal W}(A)$ consists of all alternatives $\alpha \in A$ that are uniquely optimal in some scenario $w\in {\mathcal W}$ (i.e., $\mathrm{O}_w(A) = {\{\alpha \}}$). It can be easily seen that $\mathrm{PSO}_{\mathcal W}(A) \subseteq \mathrm{PO}_{\mathcal W}(A) \cap \mathrm{UD}_{\mathcal W}(A)$.

It is convenient to have a notation for the set of scenarios in which $\alpha$ is optimal in $A$:

### Definition 8

($\mathrm{Opt}_{\mathcal W}^A(\alpha )$) We define, for $\alpha \in A$, $\mathrm{Opt}_{\mathcal W}^A(\alpha )$ to consist of all scenarios $w\in {\mathcal W}$ in which $\alpha$ is optimal in $A$, i.e., $\alpha \in \mathrm{O}_w(A)$.

Thus, $\alpha$ is possibly optimal ($\alpha \in \mathrm{PO}_{\mathcal W}(A)$) if and only if the set $\mathrm{Opt}_{\mathcal W}^A(\alpha )$ is non-empty; this is because both hold if and only if there exists a scenario $w$ in which $\alpha$ is optimal ($\alpha \in \mathrm{O}_w(A)$).

For $B\subseteq A$, the next result shows that utility-equivalence of $B$ and $A$ can be expressed in terms of the sets $\mathrm{Opt}_{\mathcal W}^A(\beta )$: we have $B\equiv _{\forall \forall \exists }^{{\mathcal W}} A$ if and only if $\bigcup _{\beta \in B} \mathrm{Opt}_{\mathcal W}^A(\beta ) = {\mathcal W}$; this follows because each is equivalent to the condition that in every scenario there exists some element of $B$ that is optimal, i.e., for all $w\in {\mathcal W}$ there exists $\beta \in B$ with $\beta \in \mathrm{O}_w(A)$.

### Lemma 1

Let ${\mathcal W}\subseteq {\mathcal U}$ and let $A\in {\mathcal M}$. For $B\subseteq A$, $B\equiv _{\forall \forall \exists }^{{\mathcal W}} A$ if and only if $\bigcup _{\beta \in B} \mathrm{Opt}_{\mathcal W}^A(\beta ) = {\mathcal W}$. In particular, $\bigcup _{\alpha \in A} \mathrm{Opt}_{\mathcal W}^A(\alpha ) = {\mathcal W}$.

We now define the maximally possibly optimal elements to be those that are optimal in a maximal set of scenarios.

### Definition 9

(*Maximally Possibly Optimal Set* $\mathrm{MPO}_{\mathcal W}(A)$.) For $A\in {\mathcal M}$, we define $\mathrm{MPO}_{\mathcal W}(A)$ to consist of all $\alpha \in A$ such that there exists no $\beta \in A$ such that $\mathrm{Opt}_{\mathcal W}^A(\beta ) \supsetneqq \mathrm{Opt}_{\mathcal W}^A(\alpha )$.

In other words, if alternative $\alpha$ in $A$ is not maximally possibly optimal in $A$ then there exists an alternative $\beta$ in $A$ that is optimal in every scenario that $\alpha$ is optimal (and at least one more scenario).

*Example* 1*continued:* We have the set of undominated elements $\mathrm{UD}_{\mathcal W}(A) = {\{ (10, 4), (7,5), (6,6), (4,7) \}}$. Abbreviating $w$ to just its first component $w_1$ we have ${\mathcal W}= [0, \frac{2}{3}]$, and $\mathrm{Opt}_{\mathcal W}^A(10,4) = [\frac{1}{3}, \frac{2}{3}]$; $\mathrm{Opt}_{\mathcal W}^A(6,6)= {\{\frac{1}{3}\}}$, $\mathrm{Opt}_{\mathcal W}^A(4,7) = [0, \frac{1}{3}]$ and $\mathrm{Opt}_{\mathcal W}^A(11,1) = \mathrm{Opt}_{\mathcal W}^A(7,5) = \emptyset$. Thus, $\mathrm{PO}_{\mathcal W}(A) = \{ (10, 4), (6,6),$ $(4,7) \}$. We have $\mathrm{PSO}_{\mathcal W}(A) = \mathrm{MPO}_{\mathcal W}(A) = {\{ (10, 4), (4,7) \}}$ and ${\{ (10, 4), (4,7) \}}$ $\equiv _{\forall \forall \exists }^{{\mathcal W}} A$. The $\mathrm{PSO}_{\mathcal W}$ operator thus leads here to stronger filtering than the $\mathrm{PO}_{\mathcal W}$ operator. In Fig. 1 we can see a graphical interpretation of $\mathrm{Opt}_{\mathcal W}^A((4,7))$ $=[0,\frac{1}{3}]$, i.e., $w_1\in [0,\frac{1}{3}]$ is an interval in which there is no line strictly above the line associated to (4, 7). We have $(4,7)\in \mathrm{PSO}_{\mathcal W}(A)$ because for any $w_1\in [0,\frac{1}{3})$ the line associated to (4, 7) is (strictly) above all the other lines, and $(6,6)\in \mathrm{PO}_{\mathcal W}(A)$ because at $w=\frac{1}{3}$ there is no line (strictly) above the line associated to (6, 6). $\square$

We now give some basic properties of the operator $\mathrm{PSO}_{\mathcal W}$. Part (i) shows that for any utility-equivalent subset $B$ of an equivalence-free set $A$ contains any possibly strictly optimal elements. Part (ii) is used to show part (iii), which implies that the relation $\succcurlyeq _{\forall \forall \exists }^{{\mathcal W}}$ could be used for computing $\mathrm{PSO}_{\mathcal W}(A)$.

### Proposition 6

Consider any ${\mathcal W}\subseteq {\mathcal U}$ and a set $A\in {\mathcal M}$.

(i) For $B\subseteq A$, if $B\equiv _{\forall \forall \exists }^{{\mathcal W}} A$ then for all $\alpha \in \mathrm{PSO}_{\mathcal W}(A)$ there exists $\beta \in B$ with $\beta \equiv _{\mathcal W}\alpha$. If, in addition, $A$ is $\equiv _{\mathcal W}$-free then $B\supseteq \mathrm{PSO}_{\mathcal W}(A)$.
(ii) If $\alpha \in A\setminus \mathrm{PSO}_{\mathcal W}(A)$ then $\bigcup _{\beta \in A\setminus {\{\alpha \}}} \mathrm{Opt}_{\mathcal W}^A(\beta ) = {\mathcal W}$ and so $A\setminus {\{\alpha \}} \equiv _{\forall \forall \exists }^{{\mathcal W}} A$.
(iii) For $\equiv _{\mathcal W}$-free $A$, $\mathrm{PSO}_{\mathcal W}(A)$ is the set of all $\alpha \in A$ such that $A\setminus {\{\alpha \}} \not \succcurlyeq _{\forall \forall \exists }^{{\mathcal W}} {\{\alpha \}}$.

*Maintaining utility equivalence:* let $A\in {\mathcal M}$ and let $\mathrm{OP}$ be a function that maps subsets of $A$ to subsets of $A$. Given ${\mathcal W}$ and its associated set of utility functions, we say that $\mathrm{OP}$ *maintains utility-equivalence* [*over* $A$] if $\mathrm{OP}(B) \equiv _{\forall \forall \exists }^{{\mathcal W}} B$ for all subsets $B$ of $A$.

The following result states relationships between the different operators, and shows that we can replace $A$ and $B$ by (for instance) $\mathrm{PO}_{\mathcal W}(A)$ and $\mathrm{PO}_{\mathcal W}(B)$, respectively, in testing $A\succcurlyeq _{\forall \forall \exists }^{{\mathcal W}} B$. It shows that $\mathrm{UD}_{\mathcal W}$, $\mathrm{MPO}_{\mathcal W}$ and $\mathrm{PO}_{\mathcal W}$ all maintain utility-equivalence for arbitrary $A$, which implies that each maps a non-empty set to a non-empty set. Example 2 shows that $\mathrm{PSO}_{\mathcal W}$ does not always maintain utility-equivalence. However, in Sects. 5.2 and 6 we give sufficient conditions for it to maintain utility-equivalence.

### Proposition 7

Assume that ${\mathcal W}\subseteq {\mathcal U}$ and $A\in {\mathcal M}$. Then the following hold:

(i) $$\mathrm{PSO}_{\mathcal W}(A) \subseteq \mathrm{MPO}_{\mathcal W}(A) \cap \mathrm{UD}_{\mathcal W}(A) \subseteq \mathrm{MPO}_{\mathcal W}(A) \subseteq \mathrm{PO}_{\mathcal W}(A) \subseteq A$$.
(ii) $\mathrm{OP}_{\mathcal W}(A)\equiv _{\forall \forall \exists }^{{\mathcal W}} A$ if $\mathrm{OP}_{\mathcal W}(A)$ is any of the following: $\mathrm{UD}_{\mathcal W}(A)$, $\mathrm{PO}_{\mathcal W}(A)$, $\mathrm{MPO}_{\mathcal W}(A)$, $\mathrm{PO}_{\mathcal W}(A) \cap \mathrm{UD}_{\mathcal W}(A)$, or $\mathrm{MPO}_{\mathcal W}(A) \cap \mathrm{UD}_{\mathcal W}(A)$. Thus, $A\succcurlyeq _{\forall \forall \exists }^{{\mathcal W}} B$ $\iff$ $\mathrm{OP}_{\mathcal W}(A) \succcurlyeq _{\forall \forall \exists }^{{\mathcal W}} \mathrm{OP}_{\mathcal W}(B)$.

An immediate consequence of part (ii) is that $\mathrm{OP}_{\mathcal W}(A)$ is non-empty if $\mathrm{OP}_{\mathcal W}(A)$ is any of the following: $\mathrm{MPO}_{\mathcal W}(A) \cap \mathrm{UD}_{\mathcal W}(A)$, $\mathrm{MPO}_{\mathcal W}(A)$, $\mathrm{PO}_{\mathcal W}(A) \cap \mathrm{UD}_{\mathcal W}(A)$, or $\mathrm{PO}_{\mathcal W}(A)$.

### Example 2

Let ${\mathcal W}= {\{w_1, w_2, w_3\}}$, let $A= {\{\alpha ,\beta ,\gamma \}}$ and suppose that $\alpha \equiv _{w_1} \beta \succ _{w_1} \gamma$; and $\beta \equiv _{w_2} \gamma \succ _{w_2} \alpha$; and $\gamma \equiv _{w_3} \alpha \succ _{w_3} \beta$. $A$ is equivalence-free, and no alternative dominates any other alternative; e.g., $\alpha \not \succ _{\mathcal W}\beta$ because $\beta \succ _{w_2} \alpha$. Thus, $\mathrm{UD}_{\mathcal W}(A) = A$. We have ${\{\alpha ,\beta \}} \equiv _{\forall \forall \exists }^{{\mathcal W}} A$ since in every scenario either $\alpha$ or $\beta$ is optimal in $A$. Similarly, ${\{\alpha ,\gamma \}}$ and ${\{\beta ,\gamma \}}$ are utility-equivalent to $A$. However, it is not the case that ${\{\alpha ,\beta \}} \equiv _{\forall \exists \forall }^{{\mathcal W}} A$ because neither $\alpha$ or $\beta$ dominate $\gamma$. We have $\mathrm{Opt}_{\mathcal W}^A(\alpha ) = {\{w_1, w_3\}}$ because $\alpha$ is optimal in scenarios $w_1$ and $w_3$, and we have $\mathrm{Opt}_{\mathcal W}^A(\beta ) = {\{w_1, w_2\}}$, and $\mathrm{Opt}_{\mathcal W}^A(\gamma ) = {\{w_2, w_3\}}$. Since these three sets are non-empty, each alternative is possibly optimal, and because none contains any other, each alternative is maximally possibly optimal. Hence, $\mathrm{PO}_{\mathcal W}(A) = \mathrm{MPO}_{\mathcal W}(A) = A$. However, there are no possibly strictly optimal alternatives, i.e. $\mathrm{PSO}_{\mathcal W}(A) = \emptyset$. The reason is that we have $\mathrm{O}_{w_1} = {\{\alpha ,\beta \}}$, and $\mathrm{O}_{w_2} = {\{\beta ,\gamma \}}$, and $\mathrm{O}_{w_3} = {\{\beta ,\gamma \}}$, so there are no uniquely optimal alternatives. Hence, $\mathrm{PSO}_{\mathcal W}(A)$ is not utility-equivalent to $A$, i.e., $\mathrm{PSO}_{\mathcal W}(A)\not \equiv _{\forall \forall \exists }^{{\mathcal W}} A$.

## Setwise-minimal equivalent subsets and the possibly strictly optimal elements

In this section we consider the issue of replacing $A$ with an (utility-)equivalent subset of $A$ that is minimal. This relates with Question (1) in the introduction. We show that, for equivalence-free set $A$ of alternatives, the set of possibly strictly optimal elements $\mathrm{PSO}_{\mathcal W}(A)$ is the intersection of all the minimal equivalent subsets. Also, we give a sufficient condition for $\mathrm{PSO}_{\mathcal W}(A)$ to be utility-equivalent with $A$ (${\mathcal W}$ being $A$-extendable), and in this case, $\mathrm{PSO}_{\mathcal W}(A)$ is the unique minimal utility-equivalent subset of $A$.

*Setwise-minimal equivalent subsets:* We may want to reduce $A$ to a utility-equivalent subset that cannot be reduced any further, i.e., there is no proper subset of it that is also utility equivalent to $A$. Theorem 1 determines when there is a unique such subset.

### Definition 10

($\text {SME}_{\mathcal W}(A)$.) We define $\text {SME}_{\mathcal W}(A)$ to be the set of subsets $B$ of $A$ that are setwise-minimal equivalent to $A$, i.e., such that $B\equiv _{\forall \forall \exists }^{{\mathcal W}} A$ and there does not exist any strict subset $C$ of $B$ such that $C\equiv _{\forall \forall \exists }^{{\mathcal W}} A$.

In Sect. 7.1 we give a simple method for finding setwise-minimal equivalent subsets.

### Relating $\text {SME}_{\mathcal W}(A)$ and $\mathrm{PSO}_{\mathcal W}(A)$ in the general case

Theorem 1 below gives some relationships between $\mathrm{PSO}_{\mathcal W}(A)$, $\text {SME}_{\mathcal W}(A)$ and the dominance relation $\succcurlyeq _{\forall \forall \exists }^{{\mathcal W}}$, for equivalence-free $A$. Any setwise-minimal equivalent subset of $A$ contains $\mathrm{PSO}_{\mathcal W}(A)$, the set of possibly strictly optimal elements. The latter set is equal to the intersection of all the setwise-minimal equivalent subsets, and is equivalent to $A$ if and only if there is a unique minimal equivalent subset, which is thus equal to $\mathrm{PSO}_{\mathcal W}(A)$.

The condition that $\mathrm{PSO}_{\mathcal W}(A)$ is equivalent to $A$ holds in the linear multi-objective case considered in Sect. 11 below (see Theorem 3), and so then $\mathrm{PSO}_{\mathcal W}(A)$ is the unique minimal equivalent subset of $A$.

#### Theorem 1

Let ${\mathcal W}\subseteq {\mathcal U}$ be a set of scenarios and assume that $A$ ($\in {\mathcal M}$) is $\equiv _{\mathcal W}$-free. Then the following hold:

(i) $$\bigcap _{B\in \text {SME}_{\mathcal W}(A)} B= \mathrm{PSO}_{\mathcal W}(A)$$;
(ii) $\mathrm{PSO}_{\mathcal W}(A) \equiv _{\forall \forall \exists }^{{\mathcal W}} A$ if and only if $\text {SME}_{\mathcal W}(A)$ is a singleton, which is if and only if $\mathrm{PSO}_{\mathcal W}(A)$ is the unique setwise-minimal equivalent subset for $A$.

#### Proof

(i): First consider any $B\in \text {SME}_{\mathcal W}(A)$, and thus, $B\equiv _{\forall \forall \exists }^{{\mathcal W}} A$. Proposition 6 implies that $B\supseteq \mathrm{PSO}_{\mathcal W}(A)$. Hence, $\bigcap _{B\in \text {SME}_{\mathcal W}(A)} B\supseteq \mathrm{PSO}_{\mathcal W}(A)$.

Conversely, consider any $\alpha \in A\setminus \mathrm{PSO}_{\mathcal W}(A)$. Proposition 6 implies $A\setminus {\{\alpha \}} \equiv _{\forall \forall \exists }^{{\mathcal W}} A$. Since $A$ is finite, there exists a subset $C$ of $A\setminus {\{\alpha \}}$ that is setwise-minimal equivalent to $A$, and so $C\in \text {SME}_{\mathcal W}(A)$ and $C\not \ni \alpha$, which implies that $\bigcap _{B\in \text {SME}_{\mathcal W}(A)} B\not \ni \alpha$. This proves that $\bigcap _{B\in \text {SME}_{\mathcal W}(A)} B\subseteq \mathrm{PSO}_{\mathcal W}(A)$, and thus, $\bigcap _{B\in \text {SME}_{\mathcal W}(A)} B= \mathrm{PSO}_{\mathcal W}(A)$.

(ii): Now let us assume that $\text {SME}_{\mathcal W}(A)$ is a singleton, say ${\{B\}}$. By definition, $B\equiv _{\forall \forall \exists }^{{\mathcal W}} A$, and, by the first part, $B= \mathrm{PSO}_{\mathcal W}(A)$, showing that $\mathrm{PSO}_{\mathcal W}(A) \equiv _{\forall \forall \exists }^{{\mathcal W}} A$.

Conversely, assume that $\mathrm{PSO}_{\mathcal W}(A) \equiv _{\forall \forall \exists }^{{\mathcal W}} A$, which implies that there exists some subset $C$ of $\mathrm{PSO}_{\mathcal W}(A)$ such that $C\in \text {SME}_{\mathcal W}(A)$. Using the first part we have $\mathrm{PSO}_{\mathcal W}(A) = \bigcap _{B\in \text {SME}_{\mathcal W}(A)} B\subseteq C\subseteq \mathrm{PSO}_{\mathcal W}(A)$. Thus, $C= \mathrm{PSO}_{\mathcal W}(A) = \bigcap _{B\in \text {SME}_{\mathcal W}(A)} B$ and so $\mathrm{PSO}_{\mathcal W}(A) \in \text {SME}_{\mathcal W}(A)$, and any element of $\text {SME}_{\mathcal W}(A)$ contains $\mathrm{PSO}_{\mathcal W}(A)$. By definition of $\text {SME}_{\mathcal W}(A)$ this implies that $\text {SME}_{\mathcal W}(A) = {\{\mathrm{PSO}_{\mathcal W}(A)\}}$. $\square$

In Example 1 of Sect. 3.3, $A$ is equivalence-free, and there is a unique minimal equivalent subset, i.e., ${\{ (10, 4), (4,7) \}}$, which is equal to $\mathrm{PSO}_{\mathcal W}(A)$. In Example 2 of Sect. 4, $A= {\{\alpha ,\beta ,\gamma \}}$ is equivalence-free. The minimal equivalent subsets are ${\{\alpha ,\beta \}}$, ${\{\beta ,\gamma \}}$ and ${\{\beta ,\gamma \}}$. Their intersection is empty, as is $\mathrm{PSO}_{\mathcal W}(A)$.

Proposition 8 relates $\mathrm{PSO}_{\mathcal W}$ to some properties of choice functions. A choice function $\mathrm{Opt}: 2^C\rightarrow 2^C$ satisfies the *Heritage* property if $\mathrm{Opt}(A) \cap B\subseteq \mathrm{Opt}(B)$ holds for any subsets $B$ and $A$ such that $B\subseteq A\subseteq C$. It satisfies the *Outcast* property if $\mathrm{Opt}(A) = \mathrm{Opt}(B)$ whenever $\mathrm{Opt}(A) \subseteq B\subseteq A\subseteq C$. A choice function $\mathrm{Opt}$ over $\Omega$ is said to be *path independent* [21, 47] if it satisfies $\mathrm{Opt}(A\cup B) = \mathrm{Opt}(\mathrm{Opt}(A)\cup B)$ for any $A, B\subseteq C$. This holds if and only if $\mathrm{Opt}$ satisfies the Heritage and Outcast properties. [1, 21]. The path independence property allows the computation of the best elements to be performed in a modular way [45, 47, 72]. The proposition shows that if $\mathrm{PSO}_{\mathcal W}$ maintains equivalence with respect to $\equiv _{\forall \forall \exists }^{{\mathcal W}}$ then it satisfies the important property of path independence. Part (i) relates to the Heritage property for choice functions, and (ii) relates to the Outcast property.

#### Proposition 8

Consider an arbitrary set of scenarios ${\mathcal W}$ ($\subseteq {\mathcal U}$) and suppose $A,B\in {\mathcal M}$ with $B\subseteq A$.

(i) $$\mathrm{PSO}_{\mathcal W}(A) \cap B\subseteq \mathrm{PSO}_{\mathcal W}(B)$$; and
(ii) if $\mathrm{PSO}_{\mathcal W}(A) \equiv _{\forall \forall \exists }^{{\mathcal W}} A$, and $\mathrm{PSO}_{\mathcal W}(A) \subseteq B$ then $\mathrm{PSO}_{\mathcal W}(B) = \mathrm{PSO}_{\mathcal W}(A)$;
(iii) if $\mathrm{PSO}_{\mathcal W}(B) \equiv _{\forall \forall \exists }^{{\mathcal W}} B$ for all $B\subseteq A$ then $\mathrm{PSO}_{\mathcal W}$ satisfies path independence on $2^A$, i.e., for any subsets $B$ and $C$ of $A$, $\mathrm{PSO}_{{\mathcal W}}(B\cup C) = \mathrm{PSO}_{{\mathcal W}}(\mathrm{PSO}_{{\mathcal W}}(B) \cup C)$.

### The case of $A$-extendable ${\mathcal W}$

In this section we consider a condition, that ${\mathcal W}$ is $A$-*Extendable*, which is sufficient for the function $\mathrm{PSO}_{{\mathcal W}}$ to maintain utility-equivalence (i.e., for the equivalence $\mathrm{PSO}_{{\mathcal W}}(B) \equiv _{\forall \forall \exists }^{{\mathcal W}} B$ to hold for all subsets $B$ of $A$); thus, by Theorem 1, given this condition, $\mathrm{PSO}_{{\mathcal W}}(A)$ is equal to the unique setwise-minimal equivalent subset for equivalence-free $A$. Because of Proposition 8(iii) above, the sufficient condition also implies that $\mathrm{PSO}_{{\mathcal W}}$, viewed as a function on $2^A$, satisfies path independence. In Sect. 6 we show that this sufficient condition holds for a large class of utility functions, including the linear case. Our algorithms for computing the minimal equivalent subset for $A$ make use of the utility-equivalence property, i.e., that $\mathrm{PSO}_{{\mathcal W}}(A) \equiv _{\forall \forall \exists }^{{\mathcal W}} A$.

Loosely speaking, ${\mathcal W}$ is $A$-*Extendable* if for every element $w$ of ${\mathcal W}$ there exists an element $w'$ whose preference ordering is more precise than (or equal to) that for $w$, and such that $w'$ totally orders non-equivalent elements of $A$. If this latter condition holds we say that $w'$ *is total over* $A$ *given* ${\mathcal W}$, and ${\mathcal W}_A^{\not =}$ is defined to be the set of all such $w'$.

#### Definition 11

[*Total* $w$ over ${\mathcal W}$, and ${\mathcal W}_A^{\not =}$.] Given $w\in {\mathcal W}\subseteq {\mathcal U}$ and $A\in {\mathcal M}$, we say that $w$ is total over $A$ given ${\mathcal W}$ if for all $\alpha ,\beta \in A$, either $\alpha \succ _w\beta$ or $\beta \succ _w\alpha$ or $\alpha \equiv _{\mathcal W}\beta$. Let ${\mathcal W}_A^{\not =}$ be the set of all $w\in {\mathcal W}$ that are total over $A$ given ${\mathcal W}$.

#### Definition 12

( $A$-Extendable.) Consider ${\mathcal W}\subseteq {\mathcal U}$ and $A\in {\mathcal M}$. For $w,w'\in {\mathcal W}$, we say that $w'$ extends $w$ over $A$ if for any $\alpha ,\beta \in A$, if $\alpha \succ _w\beta$ then $\alpha \succ _{w'} \beta$. (Thus, if $\alpha \succcurlyeq _{w'} \beta$ then $\alpha \succcurlyeq _w\beta$.)

We say that ${\mathcal W}$ is $A$-*Extendable* if for all $w\in {\mathcal W}$ there exists $w' \in {\mathcal W}$ that is total over $A$ given ${\mathcal W}$ (i.e., $w' \in {\mathcal W}_A^{\not =}$) and that extends $w$ over $A$.

Consider a set of scenarios ${\mathcal W}$ like in Example 1, and consider an arbitrary finite set $A$ of pairs of real numbers. Not every element of ${\mathcal W}$ is necessarily total over $A$. For instance, if $A$ contains the pairs (3, 2) and (0, 4) then $w= (0.4, 0.6)$ ranks (3, 2) the same as it ranks (0, 4), i.e., $(3,2) \equiv _w(0,4)$, even though $(3,2) \not \equiv _{\mathcal W}(0,4)$. However, ${\mathcal W}$ is $A$-Extendable, i.e., any scenario in ${\mathcal W}$ can be extended to one that is total over $A$. To illustrate this for $w$, consider positive $\epsilon > 0$; then $w' = (0.4 + \epsilon , 0.6 - \epsilon )$ will have $(3, 2) \succ _{w'} (0,4)$, and, if we choose $\epsilon$ to be sufficiently small we will have for all $\alpha , \beta \in A$, (i) if $\alpha \succ _w\beta$ then $\alpha \succ _{w'} \beta$; and (ii) if $\alpha \equiv _{w'} \beta$ then $\alpha = \beta$. (The fact that $A$ is finite is crucial for this to hold.) By (i), $w'$ extends $w$ over $A$, and by (ii), $w'$ is total over ${\mathcal W}$, i.e., $w'\in {\mathcal W}_A^{\not =}$.

The results of Sect. 6 show that the $$ A$$-Extendable property holds very often for continuous sets of scenarios $${\mathcal W}$$; roughly speaking, it holds if $$f_w(\alpha )$$ is a completely smooth function of $$w$$. Extendability also holds for classes of discrete $${\mathcal W}$$ such as for the sets of lexicographic preference models defined from the compositional preference languages in [70].

We give three lemmas that enable the proof of Theorem 2 below. The following result, which follows almost immediately from the definitions, shows that the distinction between $\mathrm{PSO}$ and $\mathrm{PO}$ disappears when all scenarios are total.

#### Lemma 2

Suppose that subset ${\mathcal T}$ of ${\mathcal W}$ only contains $w$ that are total over $A$ given ${\mathcal W}$. Then for all $B\subseteq A$, $\mathrm{PSO}_{{\mathcal T}}(B) = \mathrm{PO}_{{\mathcal T}}(B)$.

Note that for any $w\in {\mathcal W}_A^{\not =}$, relations $\equiv _w$ and $\equiv _{\mathcal W}$ are equal (when viewed as relations on $A$) and so, $\equiv _{{\mathcal W}_A^{\not =}} \ = \ \equiv _{\mathcal W}$; we therefore have the following result:

#### Lemma 3

Suppose that ${\mathcal W}\subseteq {\mathcal U}$ and that $A$ ($\in {\mathcal M}$) is such that ${\mathcal W}_A^{\not =}$ is non-empty. Then, for all $\alpha ,\beta \in A$, $\alpha \equiv _{{\mathcal W}_A^{\not =}} \beta$ $\iff$ $\alpha \equiv _{{\mathcal W}} \beta$.

Lemma 4 shows that, under the assumption that ${\mathcal W}$ is $A$-Extendable, reducing ${\mathcal W}$ to ${\mathcal W}_A^{\not =}$ does not affect $\mathrm{PSO}_{{\mathcal W}}$ or the relation $\succcurlyeq _{\forall \forall \exists }^{{\mathcal W}}$ on subsets of $A$. The reason is that each element of ${\mathcal W}$ is extended by some element of ${\mathcal W}_A^{\not =}$.

#### Lemma 4

Suppose that ${\mathcal W}$ is $A$-Extendable. Let ${\mathcal V}= {\mathcal W}_A^{\not =}$. Then, for any $B,C\subseteq A$, we have

(i) $$B\succcurlyeq _{\forall \forall \exists }^{{\mathcal V}} C$$ $$\iff $$ $$B\succcurlyeq _{\forall \forall \exists }^{{\mathcal W}} C$$.
(ii) $$\mathrm{PSO}_{{\mathcal V}}(B) = \mathrm{PSO}_{{\mathcal W}}(B)$$.

The theorem below shows that $\mathrm{PSO}_{{\mathcal W}}$ maintains utility-equivalence over subsets of $A$ if ${\mathcal W}$ is $A$-Extendable.

#### Theorem 2

Suppose that $A\in {\mathcal M}$ and that the set of scenarios ${\mathcal W}$ ($\subseteq {\mathcal U}$) is $A$-Extendable. Then, for any $B\subseteq A$, we have $B\equiv _{\forall \forall \exists }^{{\mathcal W}} \mathrm{PSO}_{{\mathcal W}}(B) = \mathrm{PO}_{{\mathcal W}_A^{\not =}}(B)$.

#### Proof

Let ${\mathcal V}= {\mathcal W}_A^{\not =}$, which is non-empty because ${\mathcal W}$ is $A$-Extendable. By Proposition 7, $B\equiv _{\forall \forall \exists }^{{\mathcal V}} \mathrm{PO}_{{\mathcal V}}(B)$, and so $B\equiv _{\forall \forall \exists }^{{\mathcal W}} \mathrm{PO}_{{\mathcal V}}(B)$, by Lemma 4(i). By Lemma 2, since each $w\in {\mathcal V}$ is total over $B$, we have $\mathrm{PO}_{{\mathcal V}}(B)$ is equal to $\mathrm{PSO}_{{\mathcal V}}(B)$, which equals $\mathrm{PSO}_{{\mathcal W}}(B)$ by Lemma 4(ii), completing the proof. $\square$

Theorem 2 and Proposition 8 immediately imply the corollary below, stating that extendability implies path independence of the operator $\mathrm{PSO}_{{\mathcal W}}$. (It also follows by considering the equality $\mathrm{PSO}_{{\mathcal W}}(B) = \mathrm{PO}_{{\mathcal W}_A^{\not =}}(B)$ given by Theorem 2, since for any set ${\mathcal W}'$, $\mathrm{PO}_{{\mathcal W}'}$ satisfies path independence.)

#### Corollary 1

Suppose that $A\in {\mathcal M}$ and that ${\mathcal W}$ is $A$-Extendable. Then, $\mathrm{PSO}_{{\mathcal W}}$ on $2^A$ satisfies path independence, i.e., for any subsets $B$ and $C$ of $A$, $\mathrm{PSO}_{{\mathcal W}}(B\cup C) = \mathrm{PSO}_{{\mathcal W}}(\mathrm{PSO}_{{\mathcal W}}(B) \cup C)$.

## $\mathrm{PSO}_{\mathcal W}(A)$ as unique minimal equivalent set in the continuous case

The results in this section show that for continuous sets ${\mathcal W}$ of user models, under natural assumptions on the utility functions, we have that the set of possibly strictly optimal alternatives $\mathrm{PSO}_{\mathcal W}(A)$ is the unique minimal equivalent set for $A$. In particular, we show that a particular property, the Identity property, is sufficient for ${\mathcal W}$ being $A$-Extendable (see Sect. 5.2) and hence for $\mathrm{PSO}$ maintaining utility-equivalence. The Identity property, which holds for important classes of function, states essentially that if two utility functions over ${\mathcal W}$ are equal locally, i.e., within a neighbourhood of a point in ${\mathcal W}$, then they are globally equal. This property holds for linear utility functions (where $f_w(\alpha ) = w\cdot \hat{\alpha }$) and other polynomial (and analytic) functions. The results also imply that in the linear case and when ${\mathcal W}$ is convex, $\mathrm{PSO}_{\mathcal W}(A) = \mathrm{MPO}_{\mathcal W}(A)$; this enables then a method for computing the minimal equivalent set by computing $\mathrm{MPO}_{\mathcal W}(A)$.

In this section we will be considering, for a given alternative $\alpha$, how $f_w(\alpha )$ varies as a function of $w$. It is then convenient to make the following definition:

For each $\alpha \in \Omega$, we define function $f^\alpha$ on ${\mathcal U}$ by $f^\alpha (w) = f_w(\alpha )$, for each $w\in {\mathcal U}$.

For $\alpha ,\beta \in \Omega$, and ${\mathcal W}\subseteq {\mathcal U}$ we define ${\mathcal W}_{\alpha \not =\beta } = {\{w\in {\mathcal W}\, : \,f^\alpha (w) \not = f^\beta (w) \}}$. These are the scenarios for which $\alpha$ has different utility from $\beta$. Recalling the definition of ${\mathcal W}_A^{\not =}$ from Sect. 5.2, we have, for $A\in {\mathcal M}$, that ${\mathcal W}_A^{\not =}$ is equal to the set of all elements $w$ of ${\mathcal W}$ such that $f^\alpha (w) \not = f^\beta (w)$ holds for all $\alpha , \beta \in A$ with $\alpha \not \equiv _{\mathcal W}\beta$. Thus,

$$ {\mathcal W}_A^{\not =}= \bigcap _{\alpha ,\beta \in A: \alpha \not \equiv _{\mathcal W}\beta } {\mathcal W}_{\alpha \not =\beta }. $$

We recall some basic topological definitions for subsets ${\mathcal W}$ of ${I\!R}^{p}$. For $\epsilon > 0$ let $B_\epsilon (w)$ be the set of elements of ${I\!R}^{p}$ that are within Euclidean distance $\epsilon$ of $w$, and let $B^{\mathcal W}_\epsilon (w) = B_\epsilon (w) \cap {\mathcal W}$, i.e., the set of elements of ${\mathcal W}$ that are within Euclidean distance $\epsilon$ of $w$. Subset ${\mathcal W}'$ of ${\mathcal W}$ is said to be *open* (in the standard topology of ${I\!R}^{p}$ induced on ${\mathcal W}$) if and only if for every $w\in {\mathcal W}'$ there exists some $\epsilon > 0$ such that $B^{\mathcal W}_\epsilon (w) \subseteq {\mathcal W}'$, i.e., $B_\epsilon (w) \cap {\mathcal W}\subseteq {\mathcal W}'$. For example, it can be shown that ${\mathcal W}_A^{\not =}$ and ${\mathcal W}_{\alpha \not =\beta }$ (for $\alpha ,\beta \in \Omega$) are open sets of ${\mathcal W}$.

Subset ${\mathcal W}'$ of ${\mathcal W}$ is said to be *closed* if its complement ${\mathcal W}\setminus {\mathcal W}'$ in ${\mathcal W}$ is an open set in ${\mathcal W}$. For example, it can be shown that $\mathrm{Opt}_{\mathcal W}^A(\alpha )$ (the set of elements in ${\mathcal W}$ that make $\alpha$ optimal in $A$) is always a closed set in ${\mathcal W}$. For arbitrary subset ${\mathcal W}'$ of ${\mathcal W}$, its closure $\mathrm{Cl}({\mathcal W}')$ is defined to be the intersection of all closed sets in ${\mathcal W}$ that contain ${\mathcal W}'$, which is the unique smallest closed set containing ${\mathcal W}'$. If $\mathrm{Cl}({\mathcal W}') = {\mathcal W}$ then we say that ${\mathcal W}'$ is *dense in* ${\mathcal W}$; this means that for every element of ${\mathcal W}$ there exists an arbitrarily close element of ${\mathcal W}'$.

### The Identity property

We first define the Identity property, which in Sect. 6.2 we show is sufficient for ${\mathcal W}$ being $A$-Extendable and hence for $\mathrm{PSO}_{\mathcal W}$ maintaining utility-equivalence (by Theorem 2). We go on to show that the Identity property holds for sets of linear functions of the scenario parameter $w$, as well as for sets of multivariate polynomials.

#### Definition 13

(*Identity Property*.) Let *F* be a set of continuous real-valued functions on a subset ${\mathcal W}$ of ${I\!R}^{p}$ (and similarly, for an arbitrary metric space ${\mathcal W}$). We say that *F* satisfies the *Identity property* if for every $f, g \in F$, if *f* and *g* agree on any non-empty open subset of ${\mathcal W}$ then they agree on ${\mathcal W}$. In other words, if there exists a non-empty open subset *T* of ${\mathcal W}$ such that for all $w\in T$, $f(w) = g(w)$ then for all $w\in {\mathcal W}$, $f(w) = g(w)$.

Thus, *F* satisfies the Identity property if and only if when two of the functions are locally equal then they are globally equal. Loosely speaking, it holds for classes of functions that are completely smooth.

*Observation:* If the Identity property holds for *F* and $G \subseteq F$ then the Identity property holds for *G*.

#### The Identity property for linear functions of $w$

The Identity property holds for classes of natural functions, in particular, for linear functions.

##### Proposition 9

Assume that each $\alpha$ in finite set $A$ is associated with a vector $\hat{\alpha }\in {I\!R}^{p}$. Let ${\mathcal W}$ be a convex subset of ${I\!R}^{p}$, and define, for each $\alpha \in A$, the function $f^\alpha$ by $f^\alpha (w) = \hat{\alpha }\cdot w$, representing the utility of alternative $\alpha$ in scenario $w$. Then the set of functions ${\{f^\alpha \, : \,\alpha \in A\}}$ satisfies the Identity Property.

Note that we’re not making any assumptions at all on the form of the function that maps $\alpha$ to $\hat{\alpha }$. (It can also be shown that the assumption that ${\mathcal W}$ is convex can be considerably weakened.)

#### The Identity Property for multivariate polynomials

Consider, for instance, a convex set ${\mathcal W}\subseteq {I\!R}^{p}$, and where, for each $\alpha \in A$, the function $f^\alpha$ is a multivariate polynomial function of the components of $w$. For example, the function *g* given as follows is a multivariate polynomial in ${p}= 3$ variables $w_1$, $w_2$ and $w_3$ (with these three variables being the components of $w$): $g(w) = 3w_1 -4.5 w_2 + 2w_1 w_2--3.2 w_1 w_2 w_3^2 + w_2^3 w_3^2 + 9w_1^2 w_3$.

More generally for the multivariate polynomial case, $f^\alpha (w)$ can be written as $\sum _\tau r_\tau (\alpha ) \, G_\tau (w)$ where the sum is finite and each $\tau$ corresponds with a ${p}$-dimensional vector of non-negative integer indices, and $G_\tau (w)$ is the corresponding product $w_1^{\tau (1)}\cdots w_{p}^{\tau ({p})}$, and each $r_\tau$ is a function on $A$.

If ${\mathcal W}$ is a convex set (or similarly, a finite union of convex sets of the same dimension) and for each $\alpha \in A$ we have that $f^\alpha$ is some multivariate polynomial function of the components of $w$ ($\in {\mathcal W}$) then the set of functions ${\{f^\alpha \, : \,\alpha \in A\}}$ satisfies the Identity Property.

To see that this is the case, the first step is to rewrite the multivariate polynomial in terms of *k* components $$w_i$$, where *k* is the dimension of $${\mathcal W}$$. This is always possible; for example, if $$k = {p}-1$$ then we just have the constraint $$\sum _i w_i = 1$$, so we can replace $$w_{p}$$ by $$1 - (w_1 +\cdots + w_{{p}-1})$$, and multiply out to obtain a multivariate polynomial in $${p}-1 $$ variables. To show the Identity Property we need to show that if two multivariate polynomials are equal in a neighbourhood of $$u\in {\mathcal W}$$ then they are equal on the whole of $${\mathcal W}$$. By taking the difference between the multivariate polynomials this is equivalent to: if a multivariate polynomial is equal to zero in a neighbourhood of $$u$$ then it is equal to zero on all of $${\mathcal W}$$, i.e., it is the zero function. Now, this is the case since if a multivariate polynomial $$\sum _\tau r_\tau G_\tau (v)$$ equals zero for all $$v\in {I\!R}^k$$ close to $$u$$ then each $$r_\tau $$ has to be zero. The latter fact can be shown by reasoning as follows. For any fixed values of variables $$w_1, \ldots , w_{k-1}$$ close to values $$u_1, \ldots , u_{k-1}$$ we can consider the multivariate polynomial as a (univariate) polynomial in $$w_k$$, which is zero in an interval around $$u_k$$, and thus, by a basic classical result, is equal to the zero polynomial. Hence, for each power of $$u_k$$, the corresponding coefficient is zero, where the corresponding coefficient is a multivariate polynomial in the $$k-1$$ variables $${\{w_1, \ldots , w_{k-1}\}}$$. Iterating this for $$k-1, k-2, \ldots , 1$$ implies that each coefficient $$r_\tau $$ is equal to zero, as required.

#### An example when the identity property does not hold

We have shown that the Identity property holds some for some important classes of smooth functions. Here we give an example of a very small set of functions where the Identity property fails to hold.

##### Example 3

Consider an example based on Example 2 of Sect. 4, again with $A= {\{\alpha ,\beta ,\gamma \}}$ but with ${\mathcal W}$ being a convex subset of ${I\!R}^{p}$. Suppose that disjoint non-empty regions ${\mathcal W}_1$, ${\mathcal W}_2$ and ${\mathcal W}_3$ of ${\mathcal W}$ are such that ${\mathcal W}_1 \cup {\mathcal W}_2 \cup {\mathcal W}_3 = {\mathcal W}$, and that for all $w_1 \in {\mathcal W}_1$, $w_2 \in {\mathcal W}_2$ and $w_3 \in {\mathcal W}_3$ we have $f_{w_1}(\alpha ) = f_{w_1}(\beta ) > f_{w_1}(\gamma )$; $f_{w_2}(\beta ) = f_{w_2}(\gamma ) > f_{w_2}(\alpha )$; and $f_{w_3}(\gamma ) = f_{w_3}(\alpha ) > f_{w_3}(\beta )$. As in Example 2 we have $\mathrm{PSO}_{\mathcal W}(A)$ being empty. The Identity property does not hold for the set of functions ${\{f^\alpha , f^\beta , f^\gamma \}}$ because, for instance, $f^\alpha (w_1) = f^\beta (w_1)$ for all $w_1$ in any open ball contained in ${\mathcal W}_1$, so the two functions $f^\alpha$ are $f^\beta$ are locally equal, but they are not globally equal, since $f^\alpha (w_2) \not = f^\beta (w_2)$ for $w_2 \in {\mathcal W}_2$. The functions cannot all be smooth, and in fact there must be discontinuities in the functions at the boundaries between the regions. Even if we retract the assumption that the union of the three regions covers ${\mathcal W}$, we will still have that the Identity property fails, for the same reason.

### Consequences of the identity property

The lemma below establishes equivalent forms of the Identity property. For our purposes the key implication is (a) $\Rightarrow$ (c), i.e., that the Identity property implies that ${\mathcal W}_A^{\not =}$ is dense in ${\mathcal W}$. The Identity property (a) implies the property (b) that for every non-equivalent $\alpha ,\beta \in A$, ${\mathcal W}_{\alpha \not =\beta }$ is dense in ${\mathcal W}$, i.e., for every element $w$ in ${\mathcal W}$, there is an arbitrarily close element in ${\mathcal W}$ that distinguishes $\alpha$ and $\beta$. If this were not the case, then there would be an open set (a neighbourhood) containing $w$ in which every scenario makes $\alpha$ and $\beta$ equal, i.e., $f^\alpha (w') = f^\beta (w')$ for all $w'$ in the open set; but the Identity property would then imply that $f^\alpha = f^\beta$, i.e., $\alpha$ and $\beta$ are equivalent, contradicting the assumption. From (b) it can be shown that (c) ${\mathcal W}_A^{\not =}$ is dense in ${\mathcal W}$, using the fact that ${\mathcal W}_A^{\not =}$ is a finite intersection of open dense sets ${\mathcal W}_{\alpha \not =\beta }$.

#### Lemma 5

Let $A\in {\mathcal M}$ and assume, for each $\alpha \in A$, that the function $f^\alpha$ is a real-valued continuous function on the metric space ${\mathcal W}$. The three conditions below are equivalent, i.e., if one holds then the other two also hold.

- The set of functions ${\{f^\alpha \, : \,\alpha \in A\}}$ on ${\mathcal W}$ satisfies the Identity property.
- For all $\alpha , \beta \in A$ with $\alpha \not \equiv _{\mathcal W}\beta$, we have $\mathrm{Cl}({\mathcal W}_{\alpha \not =\beta }) = {\mathcal W}$.
- $$\mathrm{Cl}({\mathcal W}_A^{\not =}) = {\mathcal W}$$.

If the topological closure $\mathrm{Cl}({\mathcal W}_A^{\not =})$ of ${\mathcal W}_A^{\not =}$ is equal to ${\mathcal W}$ then for any element $w$ in ${\mathcal W}$, we can find arbitrarily close other elements $w'$ that totally order (non-equivalent elements of) $A$. Because of the finiteness of $A$ and the continuity of the functions $f^\alpha$ this implies that we can then find such an element $w'\in {\mathcal W}$ that extends $w$, showing that ${\mathcal W}$ is $A$-Extendable.

#### Lemma 6

Let $A\in {\mathcal M}$ and assume, for each $\alpha \in A$, that the function $f^\alpha$ is a real-valued continuous function on the metric space ${\mathcal W}$. If ${\mathcal W}$ equals the topological closure $\mathrm{Cl}({\mathcal W}_A^{\not =})$ of ${\mathcal W}_A^{\not =}$ then ${\mathcal W}$ is $A$-Extendable.

Putting Lemmas 5 and 6 together we obtain the following result showing that the Identity property is a sufficient condition for ${\mathcal W}$ to be $A$-Extendable.

#### Proposition 10

Let $A\in {\mathcal M}$ and assume, for each $\alpha \in A$, that the function $f^\alpha$ is a real-valued continuous function on the metric space ${\mathcal W}$. If the set of functions ${\{f^\alpha \, : \,\alpha \in A\}}$ on ${\mathcal W}$ satisfies the Identity property then ${\mathcal W}$ is $A$-Extendable.

Proposition 10, together with Theorem 2, implies that the Identity property is sufficient for $\mathrm{PSO}_{\mathcal W}$ to maintain utility-equivalence, and the existence of unique minimal equivalent subset for a set $A$.

#### Theorem 3

Let $C\in {\mathcal M}$ and assume, for each $\alpha \in C$, that the function $f^\alpha$ is a real-valued continuous function on the metric space ${\mathcal W}$, and that the set of functions ${\{f^\alpha \, : \,\alpha \in C\}}$ satisfies the Identity property. Then the following hold, for all non-empty $A\subseteq C$.

(i) $$\mathrm{PSO}_{\mathcal W}(A) \equiv _{\forall \forall \exists }^{{\mathcal W}} A$$.
(ii) If $A$ is $\equiv _{\mathcal W}$-free then there exists a unique setwise-minimal equivalent subset for $A$, i.e., $\text {SME}_{\mathcal W}(A)$ is a singleton, and this equals $\mathrm{PSO}_{\mathcal W}(A)$.

#### Proof

(i): Since ${\{f^\alpha \, : \,\alpha \in C\}}$ satisfies the Identity property, by Proposition 10, ${\mathcal W}$ is $A$-Extendable, and thus, $\mathrm{PSO}_{\mathcal W}(A) \equiv _{\forall \forall \exists }^{{\mathcal W}} A$, using Theorem 2.

(ii): If $A$ is $\equiv _{\mathcal W}$-free then, by part (i) and Theorem 1, $\mathrm{PSO}_{\mathcal W}(A)$ is the unique setwise-minimal equivalent subset for $A$. $\square$

Theorem 3 immediately implies that $\mathrm{PSO}_{\mathcal W}$ maintains utility-equivalence for the linear case (by Proposition 9), as well as for other cases such as for multivariate polynomial utility functions. The assumption that ${\mathcal W}$ is convex can be substantially weakened, e.g., to ${\mathcal W}$ being a finite union of convex sets of the same dimension.

#### Corollary 2

Assume that each $\alpha$ in finite set $A$ is associated with a vector $\hat{\alpha }\in {I\!R}^{p}$. Let ${\mathcal W}$ be a convex subset of ${I\!R}^{p}$, and define, for each $\alpha \in A$, the function $f^\alpha$ by $f^\alpha (w) = \hat{\alpha }\cdot w$, representing the utility of alternative $\alpha$ in scenario $w$. Then we have $\mathrm{PSO}_{\mathcal W}(A) \equiv _{\forall \forall \exists }^{{\mathcal W}} A$.

We observed earlier that an alternative $\alpha$ is possibly optimal ($\alpha \in \mathrm{PO}_{\mathcal W}(A)$) if and only if the set of scenarios $\mathrm{Opt}_{\mathcal W}^A(\alpha )$ ($\subseteq {\mathcal W}$) is non-empty (see Definition 8 in Sect. 4). When the Identity property holds (including the cases of linear and polynomial utility functions), we have a related statement regarding possibly strict optimality, namely: $\alpha$ is possibly strictly optimal if and only if $\mathrm{Opt}_{\mathcal W}^A(\alpha )$ has a non-empty interior, which corresponds, at least in the linear case, with $\mathrm{Opt}_{\mathcal W}^A(\alpha )$ having the same dimension as ${\mathcal W}$ (this follows from Auxiliary Lemma 15 in the appendix, since $\mathrm{Opt}_{\mathcal W}^A(\alpha )$ is then convex). In the running example (see the continuation of Example 1 in Sect. 4), the alternative (6, 6) is possibly optimal but not possibly strictly optimal, which is reflected by $\mathrm{Opt}_{\mathcal W}^A(6,6)= {\{\frac{1}{3}\}}$, being a non-empty set of smaller dimension than ${\mathcal W}$.

#### Proposition 11

Let $C\in {\mathcal M}$ and assume, for each $\alpha \in C$, that the function $f^\alpha$ is a real-valued continuous function on the metric space ${\mathcal W}$, and that the set of functions ${\{f^\alpha \, : \,\alpha \in C\}}$ satisfies the Identity property. Suppose that $A\subseteq C$. For $\alpha \in A$, we have $\alpha \in \mathrm{PSO}_{\mathcal W}(A)$ if and only if $\mathrm{Opt}_{\mathcal W}^A(\alpha )$ contains a non-empty open set, i.e., has a non-empty interior.

We show below that for the linear case with convex ${\mathcal W}$, we have that the maximally possible optimal elements are the same as the possibly strictly optimal elements, so that $\mathrm{MPO}_{\mathcal W}=\mathrm{PSO}_{\mathcal W}$. We use this property as a basis of our algorithm for computing the set of possibly strictly optimal elements (which equals the minimal equivalent set) in Sect. 12.1.

#### Corollary 3

Assume that ${\mathcal W}$ is a convex subset of ${I\!R}^{p}$, and consider $A\in {\mathcal M}$ and assume that for each $\alpha \in A$ there exists $\hat{\alpha }\in {I\!R}^{p}$ such that for all $w\in {I\!R}^{p}$, $f_w(\alpha ) = w\cdot \hat{\alpha }$. Then $\mathrm{MPO}_{\mathcal W}(A) =\mathrm{PSO}_{\mathcal W}(A)$.

## Filtering algorithms for minimal equivalent subsets, $\mathrm{PSO}_{\mathcal W}(A)$ and $\mathrm{MPO}_{\mathcal W}(A)$

this section includes two simple kinds of filtering to reduce the input set of alternatives, both of which can be used for the computation of the possibly strictly optimal elements. In Sect. 7.1 we use a simple filtering method to compute a setwise-minimal equivalent set, and thus, in certain circumstances, $\mathrm{PSO}_{\mathcal W}(A)$ (such as when the hypotheses of Theorem 3 or Corollary 2 hold). We use this in a linear programming algorithm for computing the set of Possibly Optimal elements in Sect. 11.3 below, which is used in the method (a) in Sect. 12.1.

In Sect. 7.2 we show how the set of maximally possibly optimal elements $\mathrm{MPO}_{\mathcal W}(A)$ can be computed using a certain dominance relation between alternatives in $A$. This therefore gives another method for computing $\mathrm{PSO}_{\mathcal W}(A)$ when $\mathrm{PSO}_{\mathcal W}(A) = \mathrm{MPO}_{\mathcal W}(A)$ (cf. Corollary 3 above). We use this in the (b) algorithm in Sect. 12.1 below.

### Filtering with relations on sets

A simple way of generating a minimal equivalent subset of $A$ is to sequentially delete elements $\alpha$ of $A$ that are not needed for maintaining equivalence, i.e., such that $A\setminus {\{\alpha \}}\succcurlyeq _{\forall \forall \exists }^{{\mathcal W}} {\{\alpha \}}$, since then $A\setminus {\{\alpha \}} \equiv _{\forall \forall \exists }^{{\mathcal W}} A$. This is what is done in the operation $\textit{Filter}_\sigma (A; \succcurlyeq _{\forall \forall \exists }^{{\mathcal W}})$ defined below, to produce a minimal equivalent subset of $A$,^1^

For $\alpha \in A$, define $\textit{Filter}(A,\alpha ; \succcurlyeq _{\forall \forall \exists }^{{\mathcal W}})$ to be $A\setminus {\{\alpha \}}$ if $A\setminus {\{\alpha \}}\succcurlyeq _{\forall \forall \exists }^{{\mathcal W}} {\{\alpha \}}$; otherwise it equals $A$.

More generally, for $B\subseteq A$, we define $\textit{Filter}(A,B; \succcurlyeq )$ to be $A\setminus B$ if $A\setminus B\succcurlyeq B$; otherwise it equals $A$.

Let us label $A$ as $\alpha _1, \ldots , \alpha _n$, where $n = |A|$. Formally, the labelling is a bijection $\sigma$ from ${\{1,\ldots , n\}}$ to $A$ (so that $\sigma (i) = \alpha _i$), and let $\Lambda$ be the set of all *n*! labellings. We define $\textit{Filter}_\sigma (A; \succcurlyeq _{\forall \forall \exists }^{{\mathcal W}})$ iteratively as follows. We set $A^0 = A$. For $i=1, \ldots , n$, we set $A^i = \textit{Filter}(A^{i-1},\alpha _i; \succcurlyeq _{\forall \forall \exists }^{{\mathcal W}})$. We then define $\textit{Filter}_\sigma (A; \succcurlyeq _{\forall \forall \exists }^{{\mathcal W}})$ to be $A^n$, i.e., the set remaining after iteratively deleting elements from $A$ that are dominated with respect to relation $\succcurlyeq _{\forall \forall \exists }^{{\mathcal W}}$.

As the proposition below states, when applying the filtering operation $\textit{Filter}_\sigma (A; \succcurlyeq _{\forall \forall \exists }^{{\mathcal W}})$, (i) equivalence is always maintained; and (ii) we always obtain a minimal equivalent subset, and any such subset can be achieved for some ordering. Part (iii) implies that for any labelling $\sigma$ we have $\textit{Filter}_\sigma (A; \succcurlyeq _{\forall \forall \exists }^{{\mathcal W}}) = \mathrm{PSO}_{\mathcal W}(A)$ if $\mathrm{PSO}_{\mathcal W}(A) \equiv _{\forall \forall \exists }^{{\mathcal W}} A$ and $A$ is equivalence-free.

#### Proposition 12

Let ${\mathcal W}$ ($\subseteq {\mathcal U}$) be a set of scenarios, let $A\in {\mathcal M}$ and let $\sigma$ be any labelling of $A$. Then we have:

(i) $$A\equiv _{\forall \forall \exists }^{{\mathcal W}} \textit{Filter}_\sigma (A; \succcurlyeq _{\forall \forall \exists }^{{\mathcal W}}) \subseteq A$$.
(ii) $$\text {SME}_{\mathcal W}(A) = {\{\textit{Filter}_\sigma (A; \succcurlyeq _{\forall \forall \exists }^{{\mathcal W}}) \, : \,\sigma \in \Lambda \}}$$.
(iii) If $A$ is $\equiv _{\mathcal W}$-free and $\mathrm{PSO}_{\mathcal W}(A) \equiv _{\forall \forall \exists }^{{\mathcal W}} A$ then $\textit{Filter}_\sigma (A; \succcurlyeq _{\forall \forall \exists }^{{\mathcal W}}) = \mathrm{PSO}_{\mathcal W}(A)$ for any labelling $\sigma$.

Hence, we can generate a setwise minimal equivalent subset of $A$ by choosing any labelling $\sigma$ and computing $\textit{Filter}_\sigma (A; \succcurlyeq _{\forall \forall \exists }^{{\mathcal W}})$. Furthermore, this will be equal to $\mathrm{PSO}_{\mathcal W}(A)$, and be the unique setwise minimal equivalent subset, if the operator $\mathrm{PSO}_{\mathcal W}$ maintains utility-equivalence, such as in the linear case (see Corollary 2).

### A structure for computation of $\mathrm{MPO}_{\mathcal W}(A)$

The maximally possibly optimal elements of $A$ are those that are undominated with respect to a certain strict partial order ($\mathrm{Opt}_{\mathcal W}^A$-dominance) as defined below. This leads to a simple method for computing $\mathrm{MPO}_{\mathcal W}(A)$, by iteratively deleting elements, if one has a method for testing the $\mathrm{Opt}_{\mathcal W}^A$-dominance relation.

#### Definition 14

( $\mathrm{Opt}_{\mathcal W}^A$-dominance.) Let ${\mathcal W}$ ($\subseteq {\mathcal U}$) be a set of scenarios and let $A\in {\mathcal M}$. For $\alpha ,\beta \in \Omega$, we say that $\alpha$ $\mathrm{Opt}_{\mathcal W}^A$-dominates $\beta$ if $\alpha ,\beta \in A$ and $\mathrm{Opt}_{\mathcal W}^A(\alpha ) \supsetneqq \mathrm{Opt}_{\mathcal W}^A(\beta )$. For $\beta \in A$, we say that $\beta$ is $\mathrm{Opt}_{\mathcal W}^A$-dominated if there exists $\alpha$ such that $\alpha$ $\mathrm{Opt}_{\mathcal W}^A$-dominates $\beta$; otherwise, $\beta$ is $\mathrm{Opt}_{\mathcal W}^A$-undominated.

#### Lemma 7

Let ${\mathcal W}$ ($\subseteq {\mathcal U}$) be a set of scenarios and let $A\in {\mathcal M}$. $\mathrm{MPO}_{\mathcal W}(A)$ is the set of $\mathrm{Opt}_{\mathcal W}^A$-undominated elements (of $A$). $\mathrm{Opt}_{\mathcal W}^A$-dominance is an irreflexive transitive relation on $A$, and if $\alpha$ is $\mathrm{Opt}_{\mathcal W}^A$-dominated then there exists $\mathrm{Opt}_{\mathcal W}^A$-undominated $\beta$ that $\mathrm{Opt}_{\mathcal W}^A$-dominates $\alpha$.

The fact that $\mathrm{Opt}_{\mathcal W}^A$-dominance is a strict partial order means that one can iteratively delete $\mathrm{Opt}_{\mathcal W}^A$-dominated elements from $A$ until one reaches the set of $\mathrm{Opt}_{\mathcal W}^A$-undominated elements, i.e., $\mathrm{MPO}_{\mathcal W}(A)$. The result below formally expresses this fact.

#### Proposition 13

Let ${\mathcal W}$ ($\subseteq {\mathcal U}$) be a set of scenarios and let $A\in {\mathcal M}$. Let $A_1, \ldots , A_k$ be a sequence of sets with (i) $A_1 = A$; (ii) $A_i \supsetneqq A_{i+1}$ for $i=1, \ldots , k-1$; (iii) every element of $A_i \setminus A_{i+1}$ is $\mathrm{Opt}_{\mathcal W}^{A}$-dominated by some element in $A_i$; and (iv) every element of $A_k$ is not $\mathrm{Opt}_{\mathcal W}^{A}$-dominated by any other element of $A_k$. Then $A_k = \mathrm{MPO}_{\mathcal W}(A)$.

#### Proof

Every element of $A\setminus A_k$ is $\mathrm{Opt}_{\mathcal W}^{A}$-dominated by some element in $A_i$, for some $i\in {\{1,\ldots , k-1\}}$, so is $\mathrm{Opt}_{\mathcal W}^{A}$-dominated. Thus, by Lemma 7, $A_k \supseteq \mathrm{MPO}_{\mathcal W}(A)$. If $\alpha \in A_k$ were $\mathrm{Opt}_{\mathcal W}^{A}$-dominated, then, by Lemma 7, there would exists some $\mathrm{Opt}_{\mathcal W}^{A}$-undominated element $\beta$, (i.e., $\beta \in \mathrm{MPO}_{\mathcal W}(A)$) that $\mathrm{Opt}_{\mathcal W}^{A}$-dominates it, which would contradict (iv), since $A_k \supseteq \mathrm{MPO}_{\mathcal W}(A)$. Thus, every element of $A_k$ is $\mathrm{Opt}_{\mathcal W}^{A}$-undominated, i.e., $A_k \subseteq \mathrm{MPO}_{\mathcal W}(A)$, and hence $A_k = \mathrm{MPO}_{\mathcal W}(A)$. $\square$

## Setwise max regret

*Minimax Regret* [39, 51] is a decision criterion used in decision-making problems under uncertainty. In the context of artificial intelligence, it can be used to recommend an alternative that minimises the *max regret* (i.e., the worst-case loss) with respect to a utility function and all the available alternatives [13, 14, 17, 50]. Applications include, for example, the elicitation of multi-attribute utilities (see, e.g., [10, 18, 66]), or the elicitation of preferences for ranking and voting problems (see, e.g., [7, 8, 40]).

The utility-dominance condition $A\succcurlyeq _{\forall \forall \exists }^{{\mathcal W}} B$ states that in every scenario, the set of alternatives $A$ is at least as good as the set $B$; or, equivalently, $\textit{Ut}_A(w) \ge \textit{Ut}_B(w)$ for all $w\in {\mathcal W}$, by Proposition 3. A natural related numerical measure is setwise max regret $\textit{SMR}_{\mathcal W}(A, B)$ defined below which is a generalisation of the max regret and expresses how much worse $A$ could be than $B$, i.e., the maximum regret of choosing $A$ over $B$ [59, 62, 65]. For example, a set minimising the $\textit{SMR}$ could be used as a recommendation set in a decision-making problem.

Recommendation sets can also be used in elicitation, where they are treated as choice queries (i.e., queries of the kind “Among alternatives $\alpha$, $\beta$, and $\gamma$, which one do you prefer?") with the goal of reducing uncertainty to improve the quality of future recommendations; that is, reducing minimax regret. To minimise the number of interactions with the decision-maker, we need to carefully choose the queries to reduce the uncertainty as fast as possible. Ideally, evaluating a question at a given iteration should take into account all future questions and possible responses (e.g., [13, 34]). However, in practice, this evaluation is often carried out *myopically*. It turns out [65] that optimal recommendation sets with respect to $\textit{SMR}$ are also myopically optimal in an elicitation sense, as they ensure the highest worst-case (with respect to the possible query’s responses) reduction of minimax regret a posteriori.

### Definition 15

( $\textit{SMR}_{\mathcal W}(A, B)$.) For ${\mathcal W}\subseteq {\mathcal U}$ and finite subsets $A$ and $B$ of the set of alternatives, $\Omega$ we define the setwise max regret $\textit{SMR}_{\mathcal W}(A, B)$ to be $\sup _{w\in {\mathcal W}} \textit{Ut}_B(w) - \textit{Ut}_A(w)$.

When $A\subseteq B$, $\textit{SMR}_{\mathcal W}(A, B)$ is non-negative and equals the setwise max regret $\textit{SMR}(A,{\mathcal W})$ defined in [62]; that paper defines a method that involves finding a subset $A$ of $B$ (among a particular class of subsets, e.g., all those of a fixed cardinality *k*) that minimises $\textit{SMR}_{\mathcal W}(A, B)$.^2^$A$ can then be considered as a maximally informative query, to be used in an incremental elicitation process for finding an optimal element of $B$. $\textit{SMR}_{\mathcal W}(A, B)$ is closely related also to the notion of setwise max regret defined in [5].

Regarding $\textit{Ut}_A(w)$ as the utility achieved from set $A$ in scenario $w$ (and similarly, for $\textit{Ut}_B(w)$), we have that $\textit{SMR}_{\mathcal W}(A, B)$ is the worst-case loss of utility (or maximum regret) if we choose set $A$ instead of set $B$. For instance if $A$ is a subset of $B$, and $\textit{SMR}_{\mathcal W}(A, B)$ is very close to zero, then we might consider that $A$ is a sufficiently close approximation of $B$, simplifying the set of choices for the user. We have $\textit{SMR}_{\mathcal W}(A, B) \le 0$ if and only if $A\succcurlyeq _{\forall \forall \exists }^{{\mathcal W}} B$ (see Proposition 14 below). The problem of computing $\textit{SMR}_{\mathcal W}(A, B)$ is thus strongly related to that of determining $A\succcurlyeq _{\forall \forall \exists }^{{\mathcal W}} B$.

The definitions and results from earlier sections (apart from Sect. 6), regarding $\succcurlyeq _{\forall \forall \exists }^{{\mathcal W}}$, $\text {SME}$, $\mathrm{PO}$, $\mathrm{PSO}$ and $\mathrm{UD}$, depended only on the orderings $\succcurlyeq _w$, for $w\in {\mathcal W}$, and so were ordinal, in the sense that they are not affected by any strictly monotonic transformations of each function $f_w$ (where the monotonic transformations can be different for each $w$). However, this is not the case for $\textit{SMR}$, which has much weaker invariance properties.

We say that $\textit{SMR}_{\mathcal W}(A, B)$ *is achieved* if there exists $w\in {\mathcal W}$ such that $\textit{Ut}_B(w) - \textit{Ut}_A(w) = \textit{SMR}_{\mathcal W}(A, B)$, so that then $\textit{SMR}_{\mathcal W}(A, B)$ is equal to $\max _{w\in {\mathcal W}} \textit{Ut}_B(w) - \textit{Ut}_A(w)$. We will mainly be interested in situations in which $\textit{SMR}_{\mathcal W}(A, B)$ is achieved; this always happens, for instance, if for each $\alpha \in \Omega$, $f_w(\alpha )$ is a continuous function of $w$, and ${\mathcal W}$ is compact.

Proposition 14 shows the connections between setwise max regret and (i) utility dominance, (ii) the possibly optimal alternatives and (iii) the possibly strictly optimal alternatives. (i) relates the function $\textit{SMR}_{\mathcal W}$ with the relation $\succcurlyeq _{\forall \forall \exists }^{{\mathcal W}}$: the setwise max regret is strictly positive if and only if $A$ does not utility-dominate $B$. (ii) relates the function $\textit{SMR}_{\mathcal W}$ with the Possibly Optimal operator $\mathrm{PO}_{\mathcal W}$: setwise max regret is strictly negative if and only if no element of $B$ is possibly optimal in $A\cup B$; and (iii) with the Possibly Strictly Optimal operator $\mathrm{PSO}_{\mathcal W}$.

### Proposition 14

Consider $$A,B\in {\mathcal M}$$ and $${\mathcal W}\subseteq {\mathcal U}$$.

(i) $\textit{SMR}_{\mathcal W}(A, B) \le 0$ if and only if $A\succcurlyeq _{\forall \forall \exists }^{{\mathcal W}} B$.
(ii) If $\textit{SMR}_{\mathcal W}(A, B)$ is achieved then $\textit{SMR}_{\mathcal W}(A, B) \ge 0$ if and only if $\mathrm{PO}_{\mathcal W}(A\cup B) \cap B\not =\emptyset$.
(iii) For equivalence-free $A$, and $\alpha \in A$, we have that $\textit{SMR}_{\mathcal W}(A\setminus {\{\alpha \}}, {\{\alpha \}}) > 0$ if and only if $\mathrm{PSO}_{\mathcal W}(A) \ni \alpha$.

Proposition 15 gives a pair of important decomposability properties, with (i) being more useful computationally. We make use of (i) in the methods for computing $\textit{SMR}_{\mathcal W}(A, B)$ in Sects. 10 and 11.2. (ii) is a slight generalisation of the important Observation 4 in [62].

### Proposition 15

Consider $$A,B\in {\mathcal M}$$ and $${\mathcal W}\subseteq {\mathcal U}$$.

(i) $$\textit{SMR}_{\mathcal W}(A, B) = \max _{\beta \in B} \textit{SMR}_{\mathcal W}(A, {\{\beta \}})$$.
(ii) $$\textit{SMR}_{\mathcal W}(A, B) = \max _{\alpha \in \mathrm{PO}_{\mathcal W}(A)} \textit{SMR}_{\mathrm{Opt}_{\mathcal W}^A(\alpha )}({\{\alpha \}}, B)$$.

The following result shows that we can pre-process $A$ and $B$ using $\mathrm{UD}_{\mathcal W}$ and $\succcurlyeq _{\forall \exists \forall }^{{\mathcal W}}$ without changing the value of setwise max regret. We use this property in Sect. 9, and in the algorithmic methods in Sects. 11.3 and 12.2.

### Lemma 8

Consider any $$A,B\in {\mathcal M}$$.

(i) If $A' \equiv _{\forall \forall \exists }^{{\mathcal W}} A$ and $B' \equiv _{\forall \forall \exists }^{{\mathcal W}} B$ then $\textit{SMR}_{\mathcal W}(A', B') = \textit{SMR}_{\mathcal W}(A, B)$.
(ii) $$\textit{SMR}_{\mathcal W}(\mathrm{UD}_{\mathcal W}(A), \mathrm{UD}_{\mathcal W}(B)) = \textit{SMR}_{\mathcal W}(A, B)$$.
(iii) If $B'\subseteq B$ and $A\succcurlyeq _{\forall \exists \forall }^{{\mathcal W}} B\setminus B'$ and $\textit{SMR}_{\mathcal W}(A, B) \ge 0$ then $\textit{SMR}_{\mathcal W}(A, B') = \textit{SMR}_{\mathcal W}(A, B)$.

## Implications for incremental preference elicitation

In recent years there has been considerable focus in the AI preference community on incremental preference elicitation techniques, a form of active learning, see e.g., [6, 10, 11, 14, 20, 62, 64]. We argue that the notion of being possibly strictly optimal is important here.

Let $\alpha$ and $\beta$ be alternatives. Preference model $w$ is said to satisfy a preference statement $\alpha \ge \beta$ if $f_w(\alpha ) \ge f_w(\beta )$, i.e., $\alpha$ is at least as good as $\beta$ given $w$. For set of alternatives $A$ the preference statement $\alpha \ge A$ means $\alpha \ge \beta$ for all $\beta \in A$. Thus, for $\alpha \in A$, a scenario $w$ satisfies the preference statement $\alpha \ge A$ if and only if (given $w$) $\alpha$ is a most preferred element in $A$, $\alpha \in \mathrm{O}_w(A)$, i.e., $w$ makes $\alpha$ optimal in $A$. This holds if and only if $w\in \mathrm{Opt}_{\mathcal W}^A(\alpha )$.

In incremental elicitation a common strategy is to generate a small set of alternatives $A$, and to ask the user which element of $A$ is most preferred. If they reply “$\alpha$” then this is interpreted as $\alpha \ge A$. We will then update ${\mathcal W}$ to the set of all $w\in {\mathcal W}$ such that $\alpha$ is a most preferred option in $A$, i.e., we update ${\mathcal W}$ to $\mathrm{Opt}_{\mathcal W}^A(\alpha )$.

There can be forms of inconsistency, of different kinds, between the user answers and the model we have of the user. We say that, given set of preference models ${\mathcal W}$, alternative $\alpha$ is a *feasible answer to query* $A$ if $\mathrm{Opt}_{\mathcal W}^A(\alpha )$ is non-empty, i.e., there exists some user preference model in ${\mathcal W}$ under which $\alpha$ is optimal in $A$.

For ${\mathcal W}\subseteq {I\!R}^{p}$ we say that $\alpha$ is a *strongly feasible answer to query* $A$ (given ${\mathcal W}$) if $\mathrm{Opt}_{\mathcal W}^A(\alpha )$ has a non-empty interior (with respect to the induced topology for ${\mathcal W}$). For standard cases, e.g., when ${\mathcal W}$ is convex, this holds if and only if $\mathrm{Opt}_{\mathcal W}^A(\alpha )$ has the same dimension as ${\mathcal W}$. In the example in Fig. 1, with the query ${\{(11,1), (10, 4), (7,5), (6,6), (4,7)\}}$, the elements (11, 1) and (7, 5) are infeasible answers; e.g., (10, 4) is strongly feasible because the dimension of $\mathrm{Opt}_{\mathcal W}^A(10,4) = [\frac{1}{3}, \frac{2}{3}]$ is 1, i.e., the same dimension as ${\mathcal W}$. Alternative (6, 6) is feasible but not strongly feasible, because $\mathrm{Opt}_{\mathcal W}^A(6,6)= {\{\frac{1}{3}\}}$ and so has smaller dimension than ${\mathcal W}$.

The following result, which is an immediately consequence of Proposition 11, characterises feasible and strongly feasible answers to queries.

### Proposition 16

Consider $$A\in {\mathcal M}$$ and $${\mathcal W}\subseteq {I\!R}^{p}$$.

(i) $\alpha$ is a feasible answer to query $A$ given ${\mathcal W}$ if and only if $\alpha \in \mathrm{PO}_{\mathcal W}(A)$.
(ii) If the set of functions ${\{f^\alpha \, : \,\alpha \in A\}}$ satisfies the Identity property, we have that $\alpha$ is a strongly feasible answer to query $A$ given ${\mathcal W}$ if and only if $\alpha \in \mathrm{PSO}_{\mathcal W}(A)$.

Thus, the feasible answers are the possibly optimal elements, and the strongly feasible answers are exactly the possibly strictly optimal elements, in cases where the Identity property holds, such as for utility values that are linear or polynomial in $w$.

If the user chooses $\alpha$ from $A$, and $\alpha$ is not a feasible answer to $A$, then we get an inconsistency, since the updated ${\mathcal W}$ will be empty. Suppose now, on the other hand, $\alpha$ is not a strongly feasible answer to $A$. We can still consistently update ${\mathcal W}$, so this is a less strong kind of inconsistency; however, such an answer would be seriously troubling. For instance, suppose ${\mathcal W}\subseteq {I\!R}^{p}$, and consider any probability distribution over ${\mathcal W}$, regarding which is the true user model $w$, such that (as one would expect) the probability distribution is compatible with the measure of the sets. If $\alpha$ is not a strongly feasible answer to query $A$ then the probability that $w$ is such that $\alpha \ge A$ holds would be zero (since $\mathrm{Opt}_{\mathcal W}^A(\alpha )$ has then measure zero in ${\mathcal W}$, being of lower dimension than ${\mathcal W}$). A choice, by the user, of $\alpha$ would hence correspond with an event of probability zero.

To ensure that every answer to a query $A$ is feasible, we thus require that $\mathrm{PO}_{\mathcal W}(A) = A$. And, to ensure that every answer to $A$ is strongly feasible, we require that $\mathrm{PSO}_{\mathcal W}(A) = A$, i.e., that every element of $A$ is strictly possibly optimal in $A$.

We thus argue that the standard methods for generating queries in incremental preference learning should be modified to ensure that every element in the query set is strictly possibly optimal.^3^ Since Theorem 3 implies that $\mathrm{PSO}_{\mathcal W}(A)$ is non-empty, (and indeed equivalent to $A$) we can therefore replace a potential query $A$ by $\mathrm{PSO}_{\mathcal W}(A)$.

It is shown in [62, 63, 65] that choosing a subset $A$, of the set of available alternatives $B$, that maximises setwise regret $\textit{SMR}_{\mathcal W}(A, B)$ (among small subsets) is a desirable and well-founded choice for an informative query. However, it can easily happen that, for such a query $A$, we have $\mathrm{PSO}_{\mathcal W}(A) \not = A$ and even $\mathrm{PO}_{\mathcal W}(A) \not = A$. Such a choice of $A$ is then in danger of leading to an inconsistency, as described above. Fortunately, one can easily solve this problem by replacing $A$ by $\mathrm{PSO}_{\mathcal W}(A)$, since if $A$ maximises setwise regret then $\mathrm{PSO}_{\mathcal W}(A)$ also maximises setwise regret (assuming the Identity property, as holds for linear functions or polynomial functions of $w$) because $\textit{SMR}_{\mathcal W}(\mathrm{PSO}_{\mathcal W}(A), B) = \textit{SMR}_{\mathcal W}(A, B)$, by Theorem 3 and Lemma 8(i).

## Using extreme points of the epigraph of the utility function for testing $A\succcurlyeq _{\forall \forall \exists }^{{\mathcal W}} B$ and computing $\textit{SMR}_{\mathcal W}(A, B)$

This section derives extreme point methods for the related problems of computing utility-dominance and setwise max regret. More precisely, the method involves (what is known as) the epigraph of the utility function, which is defined below. The key result is Theorem 4 which shows that utility dominance and setwise max regret can be computed using the extreme points of the epigraph.

Computing the extreme points of convex ${\mathcal W}$ can lead for the linear case to an easy way of testing if $\alpha \succcurlyeq _{\mathcal W}\beta$ (for $\alpha ,\beta \in {I\!R}^{p}$): it is easy to see that $\alpha \succcurlyeq _{\mathcal W}\beta$ holds if and only if for each extreme point $w$ of ${\mathcal W}$, we have $w\cdot (\hat{\alpha }-\hat{\beta }) \ge 0$ [36]. Similarly, it follows immediately that standard maximum regret over the convex polytope ${\mathcal W}$ can be computed using the extreme points of ${\mathcal W}$, as observed e.g., in [55]. However, for setwise max regret it is not sufficient to consider the extreme points of ${\mathcal W}$. Here we develop a novel extreme points method for testing $A\succcurlyeq _{\forall \forall \exists }^{{\mathcal W}} B$ and computing $\textit{SMR}_{\mathcal W}(A, B)$, by moving to a higher dimensional space.

Given ${\mathcal W}$, the utility function $\textit{Ut}_{A}(w)$ (over $w\in {\mathcal W}$) can be viewed as a subset of ${\mathcal W}\times {I\!R}$, and we can test $A\succcurlyeq _{\forall \forall \exists }^{{\mathcal W}} B$ by considering such subsets. Let us define $\Gamma ({\mathcal W},A) \subseteq {\mathcal W}\times {I\!R}\subseteq {I\!R}^{p}\times {I\!R}$ to be ${\{(w,r) \, : \,w\in {\mathcal W}, r \ge \textit{Ut}_{A}(w)\}}$, i.e., the *epigraph* [15] of the utility function $\textit{Ut}_{A}$ on ${\mathcal W}$. If ${\mathcal W}$ is convex and compact and for all $\alpha \in A$, $f_w(\alpha )$ is a convex and continuous function of $w\in {\mathcal W}$, then $\Gamma ({\mathcal W},A)$ is a closed convex set. We write $\textit{Ext}(\Gamma ({\mathcal W},A))$ for the extreme points of $\Gamma ({\mathcal W},A)$.

The following result leads to two different ways of testing whether the condition $A\succcurlyeq _{\forall \forall \exists }^{{\mathcal W}} B$ holds or not. Firstly, we can compute the extreme points of both $\Gamma ({\mathcal W},A)$ and $\Gamma ({\mathcal W},A\cup B)$; by (ii), these two sets of extreme points are equal if and only if $A\succcurlyeq _{\forall \forall \exists }^{{\mathcal W}} B$. Alternatively, we can test $A\succcurlyeq _{\forall \forall \exists }^{{\mathcal W}} B$, using part (iii), after computing $\textit{Ext}(\Gamma ({\mathcal W},A))$. We can compute the pairwise max regret $\textit{SMR}_{\mathcal W}(A, B)$ as $\max _{\beta \in B} \textit{SMR}_{\mathcal W}(A, {\{\beta \}})$ (see Proposition 15), and use part (iv) below.

### Theorem 4

Consider any finite subsets $A$ and $B$ of ${I\!R}^{p}$, any $\beta \in {I\!R}^{p}$, and any compact and convex subset ${\mathcal W}$ of ${I\!R}^{p}$, and assume that for all $\alpha \in A\cup B\cup {\{\beta \}}$, $f_w(\alpha )$ is a convex and continuous function of $w\in {\mathcal W}$.

(i) $$A\succcurlyeq _{\forall \forall \exists }^{{\mathcal W}} B$$ $$\iff $$ $$\Gamma ({\mathcal W},A) \subseteq \Gamma ({\mathcal W},B)$$ $$\iff $$ $$\Gamma ({\mathcal W},A) = \Gamma ({\mathcal W},A\cup B)$$.
(ii) $A\succcurlyeq _{\forall \forall \exists }^{{\mathcal W}} B$ if and only if $\textit{Ext}(\Gamma ({\mathcal W},A)) = \textit{Ext}(\Gamma ({\mathcal W},A\cup B))$.
(iii) $A\succcurlyeq _{\forall \forall \exists }^{{\mathcal W}} B$ holds if and only if for all $(w,r) \in \textit{Ext}(\Gamma ({\mathcal W},A))$ and for all $\beta \in B$ we have $f_w(\beta ) \le r$.
(iv) $$\textit{SMR}_{\mathcal W}(A, {\{\beta \}}) = \max {\{f_w(\beta ) - r \, : \,(w,r) \in \textit{Ext}(\Gamma ({\mathcal W},A)) \}}$$.

Continuing the running example, it can be seen from Fig. 1 that the set $\textit{Ext}(\Gamma ({\mathcal W},A))$ of the extreme points of the epigraph is equal to $\{(0, 7), (\frac{1}{3}, 6)$ $(\frac{2}{3}, 8)\}$, where we are again abbreviating $w$ to just its first component $w_1$, so that e.g., $(\frac{1}{3}, 6)$ represents the pair $(w, \textit{Ut}_A(w))$ with $w= (\frac{1}{3}, \frac{2}{3})$. Then, using Theorem 4(iv), $\textit{SMR}_{\mathcal W}(A, {\{(5.5, 6.5)\}})$ is equal to $\max (-0.5, \frac{1}{6}, -2\frac{1}{6}) = \frac{1}{6} > 0$; for instance, the middle term in the max equals $f_w((5.5, 6.5)) - 6 = \frac{1}{3} \cdot 5.5 + \frac{2}{3}\cdot 6.5 - 6 =\frac{1}{6}$. Because $\textit{SMR}_{\mathcal W}(A, {\{(5.5, 6.5)\}})$ is strictly positive, we have that $A\not \succcurlyeq _{\forall \forall \exists }^{{\mathcal W}} {\{(5.5, 6.5)\}}$, using Proposition 14(i). Note that the key extreme point $(\frac{1}{3}, 6)$ of the epigraph does not involve any extreme point of ${\mathcal W}$, so this example illustrates the fact that it is not sufficient to just consider the extreme points of ${\mathcal W}$.

## The linear and convex case

Here we focus on the case in which the utility $f_w(\alpha )$ is a linear function of $w$, and when the set of scenarios ${\mathcal W}$ is a compact and convex subset of ${I\!R}^{p}$. The results are of most interest when ${\mathcal W}$ is also a convex polytope, and thus expressible in terms of linear constraints (so is equal to the intersection of closed half-spaces).

Section 11.1 considers the computation of dominance with respect to the relations $\succcurlyeq _{\forall \exists \forall }^{{\mathcal W}}$ and $\succcurlyeq _{\exists \forall \forall }^{{\mathcal W}}$, making use of the extreme points of ${\mathcal W}$. These are useful as sufficient conditions for utility-dominance for the convex polytope case, since then the set of extreme points is finite. In Sect. 11.2 we give a result that, for the convex polytope case, leads to a straightforward linear programming method for computing setwise max regret and hence (because of the relationship between the two shown in Proposition 14(i)) utility-dominance.

In Sect. 10 we showed how the extreme points of the epigraph of the utility function can be used to compute utility-dominance. Section 11.4 shows how the extreme points of the epigraph can be used to compute the minimal equivalent subset. In particular, it is shown how to compute $\mathrm{Opt}_{\mathcal W}^A$-dominance, and thus, by the method of Sect. 7.2, the set of maximally possibly optimal elements; the results of Sect. 6 (Theorem 3, Corollary 2 and Corollary 3) imply that this is equal to the minimal equivalent subset (of an equivalence-free set of alternatives).

We now consider the situation in which we are especially interested, where an alternative $\alpha$ in $\Omega$ corresponds with a multi-attribute utility vector $\hat{\alpha }$, and the utility functions are linear in the parameter $w$, with $f_w(\alpha ) = \hat{\alpha }\cdot w$, i.e., $\sum _{i=1}^{p}w_i \hat{\alpha }_i$, and ${\mathcal W}$ is a compact convex subset of ${I\!R}^{p}$. We therefore have, for $\alpha , \beta \in \Omega$, $\alpha \succcurlyeq _w\beta$ if and only if $(\hat{\alpha }-\hat{\beta })\cdot w\ge 0$. Also, $\textit{Ut}_A(w) = \max _{\alpha \in A} w\cdot \hat{\alpha }$.

Of particular interest in the case in which ${\mathcal W}$ is also a convex polytope, being defined by a finite number of linear inequalities. Given a finite set $\Lambda = {\{\lambda _i \, : \,i=1,\ldots , k\}}$ of vectors in ${I\!R}^{p}$, and corresponding real numbers $r_i$, we can define ${\mathcal W}$ to be the set of $w\in {\mathcal U}$ such that for all $i=1,\ldots , k$, $w\cdot \lambda _i \ge r_i$. In particular, such linear inequalities can arise from input preferences of the form $\alpha$ *is preferred to* $\beta$, leading to the constraint $w\cdot (\hat{\alpha }-\hat{\beta }) \ge 0$.

This form of preferences has been studied a great deal; for instance, $\mathrm{UD}_{\mathcal W}(A)$ consists of the non-dominated alternatives in $A$ for a multiobjective program (MOP) given a cone (with the cone generated as the dual of ${\mathcal W}$) [25, 69, 74]. Often the elements of ${\mathcal W}$ are assumed to be non-negative and normalised, so for each $w\in {\mathcal W}$ we have for all $i=1, \ldots , {p}$, $w_i \ge 0$, and $\sum _{i=1}^{p}w_i = 1$. Then, without any additional preferences (so that ${\mathcal W}$ is just the unit $({p}-1)$-simplex), relation $\succcurlyeq _{\mathcal W}$ is the Pareto ordering on alternatives, and $\mathrm{UD}_{\mathcal W}(A)$ is set of Pareto-optimal alternatives, with the supported alternatives being also in $\mathrm{PO}_{\mathcal W}(A)$. $\mathrm{UD}_{\mathcal W}(A)$ can be computed by discarding the alternatives $\beta \in A$ if there exists $\alpha \in A$ such that $f_w(\alpha ) \ge f_w(\beta )$ for all the extreme points $w$ of ${\mathcal W}$, and $f_w(\alpha ) > f_w(\beta )$ for at least one extreme points $w$ of ${\mathcal W}$ (see, e.g., [36]). $\mathrm{PO}_{\mathcal W}(A)$ can be computed by discarding all alternatives $\beta \in A$ for which there does not exists $w\in {\mathcal W}$ such that $f_w(\beta ) \ge f_w(\alpha )$ for all $\alpha \in A\setminus \{\beta \}$, which can be checked by testing the satisfiability of the constraints with a linear programming solver (see, e.g., [2, 4]).

### Testing $A\succcurlyeq _{\forall \exists \forall }^{{\mathcal W}} B$ and $A\succcurlyeq _{\exists \forall \forall }^{{\mathcal W}} B$

The following simple result is useful for computing relations $\succcurlyeq _{\forall \exists \forall }^{{\mathcal W}}$ and $\succcurlyeq _{\exists \forall \forall }^{{\mathcal W}}$ when ${\mathcal W}$ is a compact convex polytope (when its set of extreme points is finite). For $E\subseteq {I\!R}^{p}$, $\textit{CH}(E)$ is defined to be the convex hull of *E*.

#### Proposition 17

Assume that for $w\in {I\!R}^{p}, \alpha \in {I\!R}^{p}$, $f_w(\alpha ) = w\cdot \hat{\alpha }$. Let ${\mathcal W}, {\mathcal W}' \subseteq {I\!R}^{p}$. If $\textit{CH}({\mathcal W}) = \textit{CH}({\mathcal W}')$ then $\succcurlyeq _{\mathcal W}$ $=$ $\succcurlyeq _{{\mathcal W}'}$. In particular, if ${\mathcal W}$ is a compact convex subset of ${I\!R}^{p}$ and ${\mathcal W}^0 = \textit{Ext}({\mathcal W})$ is the set of extreme points of ${\mathcal W}$ then $\succcurlyeq _{\mathcal W}$ $=$ $\succcurlyeq _{{\mathcal W}^0}$. Furthermore, binary relations $\succcurlyeq _{\forall \exists \forall }^{{\mathcal W}}$ and $\succcurlyeq _{\forall \exists \forall }^{{\mathcal W}^0}$ are equal; and $\succcurlyeq _{\exists \forall \forall }^{{\mathcal W}}$ equals $\succcurlyeq _{\exists \forall \forall }^{{\mathcal W}^0}$, and $A\succcurlyeq _{\exists \forall \forall }^{{\mathcal W}} B$ holds if and only if there exists $\alpha \in A$ such that for all $w\in {\mathcal W}^0$, $f_w(\alpha ) \ge \textit{Ut}_B(w)$.

Because of Proposition 17, there is a simple way of testing if $\alpha \succcurlyeq _{\mathcal W}\beta$ (for $\alpha ,\beta \in {I\!R}^{p}$): $\alpha \succcurlyeq _{\mathcal W}\beta$ holds if and only if for each extreme point $w$ of ${\mathcal W}$, we have $w\cdot (\hat{\alpha }-\hat{\beta }) \ge 0$. This can then be used for the relations $\succcurlyeq _{\forall \exists \forall }^{{\mathcal W}}$ and $\succcurlyeq _{\exists \forall \forall }^{{\mathcal W}}$, using, for example, $A\succcurlyeq _{\forall \exists \forall }^{{\mathcal W}} B$ if and only if for all $\beta \in B$ there exists $\alpha \in A$ such that $\alpha \succcurlyeq _{\mathcal W}\beta$.

In Sect. 10 we gave a method, based on the extreme points of the epigraph of the utility function, for computing $\textit{SMR}_{\mathcal W}$ and testing dominance; in Sect. 11.2 we give a straightforward LP method related to the approaches used in [5, 10, 62]. In Sect. 11.4 we give a result that enables one to compute the minimal equivalent subset using the extreme points of the epigraph.

### Linear programming for computing $\textit{SMR}_{\mathcal W}(A, B)$, and testing $A\succcurlyeq _{\forall \forall \exists }^{{\mathcal W}} B$

The definitions easily imply that $\textit{SMR}_{\mathcal W}(A, {\{\beta \}})$ equals $\max _{w\in {\mathcal W}} f_w(\beta ) - \textit{Ut}_A(w)$. Thus, for real-valued *x*, we have $\textit{SMR}_{\mathcal W}(A, {\{\beta \}}) \ge x$ if and only if there exists $w\in {\mathcal W}$ such that for all $\alpha \in A$, $w\cdot (\hat{\beta }-\hat{\alpha }) \ge x$. This leads to the following characterisation.

#### Proposition 18

Assume that ${\mathcal W}$ is a compact subset of ${I\!R}^{p}$, and that for $w\in {I\!R}^{p}, \alpha \in {I\!R}^{p}$, $f_w(\alpha ) = w\cdot \hat{\alpha }$. Consider $A\in {\mathcal M}$ and $\beta \in {I\!R}^{p}$. Then $\textit{SMR}_{\mathcal W}(A, {\{\beta \}})$ is equal to the maximum value of *x* such that there exists $w\in {I\!R}^{p}$ satisfying the constraints (i) $w\in {\mathcal W}$; and (ii) for all $\alpha \in A$, $w\cdot (\hat{\beta }-\hat{\alpha }) \ge x$.

For the case in which ${\mathcal W}$ is a compact convex polytope, we can use a linear programming solver to compute $\textit{SMR}_{\mathcal W}(A, {\{\beta \}})$. Applying this for each $\beta \in B$ allows us to compute $\textit{SMR}_{\mathcal W}(A, B)$, which, by Proposition 15, equals $\max _{\beta \in B} \textit{SMR}_{\mathcal W}(A, {\{\beta \}})$ (see, e.g., [62, 65]). We can also use this method to test if $A\succcurlyeq _{\forall \forall \exists }^{{\mathcal W}} B$, since, by Proposition 14(i), $A\succcurlyeq _{\forall \forall \exists }^{{\mathcal W}} B$ holds if and only if $\textit{SMR}_{\mathcal W}(A, B) \le 0$, i.e., if and only if for all $\beta \in B$, $\textit{SMR}_{\mathcal W}(A, {\{\beta \}}) \le 0$.

An optimal recommendation set of a given size *k* with respect to $\textit{SMR}$ is also myopically optimal in an elicitation sense [65]. A straightforward approach to compute a subset $A$ of $B$ with $|A|=k$ with minimum setwise max regret is computing $\textit{SMR}_{\mathcal W}(A, B)$ for all the subsets $A$ with $|A|=k$. However, this approach is very computational demanding, and in the literature we can find alternative heuristic strategies based on the max regret to compute queries for elicitation purposes (see, e.g., [14, 62, 65]). In [59], we proposed an efficient branch and bound method to compute the set with minimum setwise max regret, which allows avoiding the computation of the setwise max regret for some subsets. In the latter work, we used the novel algorithm to compute $\textit{SMR}_{\mathcal W}(A, B)$ presented in this paper (see Sect. 12.2) showing better time performance with respect to the standard method based on linear programming for the values *k* and *p* considered. ([62, 65]).

### Linear programming for computing the minimal equivalent subset and the possibly strictly optimal elements

From Theorem 3 and Corollary 2 it follows that $\mathrm{PSO}_{\mathcal W}(A)$ is the minimal equivalent subset for the $\equiv _{\mathcal W}$-free set $A\in {\mathcal M}$. Because of Proposition 12, we can compute $\mathrm{PSO}_{\mathcal W}(A)$ with the method $\textit{Filter}_\sigma (A; \succcurlyeq _{\forall \forall \exists }^{{\mathcal W}})$ defined in Sect. 7.1. This iteratively excludes elements $\alpha$ from $A$ if $A\setminus {\{\alpha \}}\succcurlyeq _{\forall \forall \exists }^{{\mathcal W}} {\{\alpha \}}$, i.e., $\alpha \not \in \mathrm{PSO}_{\mathcal W}(A)$. At each iteration *i* we have then a set $A^i$ such that $\mathrm{PSO}_{\mathcal W}(A)\subseteq A^i\subseteq A$. Thus, since $\mathrm{PSO}_{\mathcal W}(A)\equiv _{\forall \forall \exists }^{{\mathcal W}}A$, we have also that $A^i\equiv _{\forall \forall \exists }^{{\mathcal W}}A$, which implies then $A^i\setminus {\{\alpha \}}\equiv _{\forall \forall \exists }^{{\mathcal W}}A\setminus {\{\alpha \}}$. Thus, using Lemma 8(i) and Proposition 14(iii), we can exclude $\alpha _i$ from $A^i$ at each iteration *i* if $\textit{SMR}_{\mathcal W}(A^i\setminus {\{\alpha _i\}}, {\{\alpha _i\}}) \le 0$, where $\textit{SMR}_{\mathcal W}(A^i\setminus {\{\alpha _i\}}, {\{\alpha _i\}})$ can be computed following the linear programming approach of Proposition 18.

### Using the extreme points of the epigraph to compute the Minimal Equivalent Subset, and the set of possibly optimal elements

We next prove some key properties relating to the sets $\mathrm{Opt}_{\mathcal W}^A(\alpha )$ and their relationship with the extreme points of the epigraph of the utility function. Recall that $\mathrm{Opt}_{\mathcal W}^A(\alpha )$ is the set of scenarios in ${\mathcal W}$ in which $\alpha$ is optimal in $A$ (see Definition 8). It is convenient to have an abbreviation ($E_{\mathcal W}^A(\alpha )$) for the set $\textit{Ext}(\mathrm{Opt}_{\mathcal W}^A(\alpha ))$.

#### Definition 16

($E_{\mathcal W}^A(\alpha )$.) For $\alpha \in A\in {\mathcal M}$ and ${\mathcal W}\subseteq {I\!R}^{p}$ we define $E_{\mathcal W}^A(\alpha )$ to be $\textit{Ext}(\mathrm{Opt}_{\mathcal W}^A(\alpha ))$, the set of extreme points of $\mathrm{Opt}_{\mathcal W}^A(\alpha )$ (see Definition 8).

For the linear case with convex ${\mathcal W}$, for $\alpha \in A$, the set $\mathrm{Opt}_{\mathcal W}^A(\alpha )$ is convex. Theorem 3 implies that $\mathrm{PSO}_{\mathcal W}(A)$ is the unique minimal equivalent subset of an (equivalence-free) set $A\in {\mathcal M}$. Corollary 3 implies that $\mathrm{PSO}_{\mathcal W}(A) = \mathrm{MPO}_{\mathcal W}(A)$, and, by definition, $\mathrm{MPO}_{\mathcal W}(A)$ consists of all $\alpha \in A$ such that there does not exist $\beta \in A$ such that $\mathrm{Opt}_{\mathcal W}^A(\beta ) \supsetneqq \mathrm{Opt}_{\mathcal W}^A(\alpha )$, i.e., such that $\beta$ $\mathrm{Opt}_{\mathcal W}^A$-dominates $\alpha$ (see Definition 14 in Sect. 7.2). Hence, $\mathrm{PSO}_{\mathcal W}(A)$ is equal to the set of $\mathrm{Opt}_{\mathcal W}^A$-undominated elements of $A$.

It turns out (see part (ii) of Proposition 19 below) that the condition $\mathrm{Opt}_{\mathcal W}^A(\beta ) \supsetneqq \mathrm{Opt}_{\mathcal W}^A(\alpha )$ is equivalent to $E_{\mathcal W}^A(\beta ) \supsetneqq E_{\mathcal W}^A(\alpha )$. This is the basis of our method, described in Sect. 12.1 below, for efficiently computing the minimal equivalent set $\mathrm{PSO}_{\mathcal W}(A)$ using the extreme points of the epigraph.

#### Proposition 19

Assume that ${\mathcal W}$ is a convex subset of ${I\!R}^{p}$, and that for $w\in {I\!R}^{p}, \alpha \in {I\!R}^{p}$, $f_w(\alpha ) = w\cdot \hat{\alpha }$. Consider $A\in {\mathcal M}$, $\alpha \in A$, $w\in {\mathcal W}$.

(i) For any $\alpha \in A$, $\mathrm{Opt}_{\mathcal W}^A(\alpha )$ is a convex subset of ${\mathcal W}$.
(ii) If ${\mathcal W}$ is compact and $\alpha ,\beta \in A$, then $\mathrm{Opt}_{\mathcal W}^A(\alpha ) \subseteq \mathrm{Opt}_{\mathcal W}^A(\beta )$ $\iff$ $E_{\mathcal W}^A(\alpha ) \subseteq E_{\mathcal W}^A(\beta )$, i.e., $\textit{Ext}(\mathrm{Opt}_{\mathcal W}^A(\alpha )) \subseteq \textit{Ext}(\mathrm{Opt}_{\mathcal W}^A(\beta ))$.

The second part of Proposition 19 may seem surprising, since it certainly does not generally hold for convex sets (for instance, consider a convex set $C_1$ that contains another convex set $C_2$, but where their boundaries are disjoint; then $\textit{Ext}(C_1) \cap \textit{Ext}(C_2) = \emptyset$).^4^

Part (i) of the following result gives a way of computing $E_{\mathcal W}^A(\alpha )$: by projecting the extreme points of the epigraph, $\textit{Ext}(\Gamma ({\mathcal W},A))$ onto their ${\mathcal W}$ component. Part (ii) leads to a useful sufficient conditions for an element $\alpha$ to be $\mathrm{Opt}_{\mathcal W}^A$-dominated in Proposition 21(iii) below.

#### Proposition 20

Assume that ${\mathcal W}$ is a convex subset of ${I\!R}^{p}$, and that for $w\in {I\!R}^{p}, \alpha \in {I\!R}^{p}$, $f_w(\alpha ) = w\cdot \hat{\alpha }$. Consider $A\in {\mathcal M}$, $w\in {\mathcal W}$, and $\alpha ,\beta \in A$.

(i) $$E_{\mathcal W}^A(\alpha ) = {\{w\in {I\!R}^{p}\, : \,(w,w\cdot \hat{\alpha }) \in \textit{Ext}(\Gamma ({\mathcal W},A)) \}}$$.
(ii) If ${\mathcal W}$ is compact then $\textit{dim}(\mathrm{Opt}_{\mathcal W}^A(\alpha )) < |E_{\mathcal W}^A(\alpha )|$.

The next proposition gives properties that are the basis for the extreme points method for computing the unique minimal equivalent subset (which equals $\mathrm{PSO}_{\mathcal W}(A) = \mathrm{MPO}_{\mathcal W}(A)$) for the linear convex case. Recall that $\beta$ $\mathrm{Opt}_{\mathcal W}^A$-dominates $\alpha$ if and only if $\mathrm{Opt}_{\mathcal W}^A(\alpha ) \subsetneqq \mathrm{Opt}_{\mathcal W}^A(\beta )$ (see Definition 14).

#### Proposition 21

Assume that ${\mathcal W}$ is a compact convex subset of ${I\!R}^{p}$, and that for $w\in {I\!R}^{p}, \alpha \in {I\!R}^{p}$, $f_w(\alpha ) = w\cdot \hat{\alpha }$. Consider $A\in {\mathcal M}$ and $\alpha ,\beta \in A$.

(i) $\beta$ $\mathrm{Opt}_{\mathcal W}^A$-dominates $\alpha$ if and only if $E_{\mathcal W}^A(\alpha ) \subsetneqq E_{\mathcal W}^A(\beta )$,
(ii) If $E_{\mathcal W}^A(\alpha ) =E_{\mathcal W}^A(\beta )$ and $\alpha \not \equiv _{\mathcal W}\beta$ then both $\alpha$ and $\beta$ are $\mathrm{Opt}_{\mathcal W}^A$-dominated.
(iii) If $|E_{\mathcal W}^A(\alpha )| \le \textit{dim}({\mathcal W})$ then $\alpha$ is $\mathrm{Opt}_{\mathcal W}^A$-dominated.

Because of Proposition 13(i) we can compute $\mathrm{MPO}_{\mathcal W}(A)$ (which, by Corollary 3 equals $\mathrm{PSO}_{\mathcal W}(A)$) by incrementally deleting $\mathrm{Opt}_{\mathcal W}^A$-dominated elements from $A$ (and redefining $A$ to be the reduced set). By Proposition 21, $\beta$ $\mathrm{Opt}_{\mathcal W}^A$-dominates $\alpha$ if and only if $E_{\mathcal W}^A(\alpha ) \subsetneqq E_{\mathcal W}^A(\beta )$, where the sets $E_{\mathcal W}^A(\alpha )$ for $\alpha \in A$ can be computed using Proposition 20.

Proposition 21 gives also extra useful sufficient conditions for an element $\alpha$ to be $\mathrm{Opt}_{\mathcal W}^A$-dominated. In those cases, the proofs that $\alpha$ is $\mathrm{Opt}_{\mathcal W}^A$-dominated makes use of the equality between $\mathrm{MPO}_{\mathcal W}(A)$ and $\mathrm{PSO}_{\mathcal W}(A)$. (To use these sufficient conditions, we do not, of course, need to find any $\mathrm{Opt}_{\mathcal W}^A$-dominating element $\beta$ of $\alpha$—it is sufficient to know implicitly that such an element $\beta$ has to exist.)

## The structure of the algorithms

In this section we make use of mathematical results in previous sections in developing computational methods for computing the minimal equivalent set $\mathrm{PSO}_{\mathcal W}(A)$ and testing dominance between sets, for the case of multi-attribute utility vectors, with the set of scenarios ${\mathcal W}$ being a convex polytope, and with linear utility functions.

### Computing the minimal equivalent set and the set of Possibly Optimal elements

Given $A\in {\mathcal M}$, we aim to generate a subset $A' \subseteq A$ with $A' \equiv _{\forall \forall \exists }^{{\mathcal W}} A$, and such that for any strict subset $A''$ of $A'$, $A'' \not \equiv _{\forall \forall \exists }^{{\mathcal W}} A$. Theorem 3 implies that there exists a unique minimal equivalent set, i.e., $\text {SME}_{\mathcal W}(A)$ has a unique element, say, $A'$, and this equals $\mathrm{PSO}_{\mathcal W}(A)$. To compute $\mathrm{PSO}_{\mathcal W}(A)$, first we pre-process by eliminating elements of $A$ not in $\mathrm{UD}_{\mathcal W}(A)$ since from Proposition 7 it follows that $\mathrm{PSO}_{\mathcal W}(A)\subseteq \mathrm{UD}_{\mathcal W}(A)$. At the same time we can make $A$ equivalence-free. Then we have two different methods for computing $\mathrm{PSO}_{\mathcal W}(A)$.

- Multiple (i.e., $|A|$) tests of the form $A\setminus {\{\alpha \}} \succcurlyeq _{\forall \forall \exists }^{{\mathcal W}} {\{\alpha \}}$, which can be achieved using a linear programming approach as described in Sect. 11.3.
- For each $\alpha \in A$ we compute $E_{\mathcal W}^A(\alpha )$ using Proposition 20(i), by computing the extreme points of the epigraph. We eliminate any element $\alpha$ such that $|E_{\mathcal W}^A(\alpha )| \le \textit{dim}({\mathcal W})$, by Proposition 21(iii) of Sect. 11.4. For each pair $\alpha ,\beta$ of the remaining elements, we eliminate $\alpha$ if $E_{\mathcal W}^A(\alpha )\subsetneqq E_{\mathcal W}^A(\beta )$; we eliminate $\beta$ if $E_{\mathcal W}^A(\beta )\subsetneqq E_{\mathcal W}^A(\alpha )$; and we eliminate both if $E_{\mathcal W}^A(\beta )=E_{\mathcal W}^A(\alpha )$, which is justified by Proposition 21(i) and Proposition 21(ii) of Sect. 11.4. Finally we arrive at a set of $\mathrm{Opt}_{\mathcal W}^A$-undominated alternatives, which by Proposition 13 is equal to $\mathrm{MPO}_{\mathcal W}(A)$, and thus equal to $\mathrm{PSO}_{\mathcal W}(A)$ by Corollary 3.

Regarding the computation of the set $\mathrm{PO}_{\mathcal W}(A)$ of possibly optimal elements of $A$ in ${\mathcal W}$, also in this case we can first compute $\mathrm{UD}_{\mathcal W}(A)$ and make $A$ equivalence free. Then we can use one of the following two methods.

(i) A well-known approach in the literature (see, e.g., [4]) consists into iteratively excluding elements $\alpha$ from $A$ if $\alpha \not \in \mathrm{PO}_{\mathcal W}(A)$. This iterative procedure is similar to that described in Sect. 7.1. The difference is regarding the method used to evaluate the exclusion of the elements of $A$ since in this case we want to compute $\mathrm{PO}_{\mathcal W}(A)$. At each iteration *i* we have then a set $A^i$ such that $\mathrm{PO}_{\mathcal W}(A)\subseteq A^i\subseteq A$, and we have that $\alpha _i\in \mathrm{PO}_{\mathcal W}(A)$ (and then $\alpha _i\in A^{i+1}$) if and only if there exists $w\in {\mathcal W}$ such that $(\hat{\alpha }_i-\hat{\alpha })\cdot w\ge 0$ for all $\alpha \in A^i {\setminus } \{\alpha _i\}$, which can be evaluated with a LP solver.
(ii) We can use Proposition 20(i) to compute $E_{\mathcal W}^A(\alpha )$ for each $\alpha \in A$ evaluating the extreme points of the epigraph of $A$ over ${\mathcal W}$. Then, since $\alpha \in \mathrm{PO}_{\mathcal W}(A)$ if and only if $\mathrm{Opt}_{\mathcal W}^A(\alpha )\ne \emptyset$, which is if and only if $|E_{\mathcal W}^A(\alpha )|>0$, we can eliminate $\alpha$ from $A$ if $|E_{\mathcal W}^A(\alpha )|=0$. The resulting set equals $\mathrm{PO}_{\mathcal W}(A)$.

### Testing dominance between sets $A\succcurlyeq _{\forall \forall \exists }^{{\mathcal W}} B$ and computing $\textit{SMR}_{\mathcal W}(A, B)$

We focus first on testing the dominance condition $A\succcurlyeq _{\forall \forall \exists }^{{\mathcal W}} B$, for given finite sets $A,B$ of alternatives, i.e., $A,B\in {\mathcal M}$. Our algorithm includes three steps of increasing complexity, with the first two steps being pre-processing that helps the efficiency of the algorithm.

The first stage of the algorithm is a pre-processing step, using a necessary and a sufficient condition for dominance between sets. Proposition 22(i) below shows that $A\succcurlyeq _{\forall \forall \exists }^{{\mathcal W}^0} B$ is a necessary condition for $A\succcurlyeq _{\forall \forall \exists }^{{\mathcal W}} B$, and $A\succcurlyeq _{\exists \forall \forall }^{{\mathcal W}^0} B$ is a sufficient condition. Part (i) follows using Proposition 17 and nestedness (Proposition 1), and monotonicity with respect to ${\mathcal W}$ (Proposition 4). Parts (ii) and (iii), which follow from Proposition 3, give efficient methods of computing the necessary condition and the sufficient condition.

#### Proposition 22

Assume that for $w\in {I\!R}^{p}, \alpha \in {I\!R}^{p}$, $f_w(\alpha ) = w\cdot \hat{\alpha }$. Let ${\mathcal W}$ be a compact and convex subset of ${I\!R}^{p}$ and let ${\mathcal W}^0 = \textit{Ext}({\mathcal W})$ be the set of extreme points of ${\mathcal W}$. Then

(i) $$A\succcurlyeq _{\exists \forall \forall }^{{\mathcal W}^0} B\ \Rightarrow \ A\succcurlyeq _{\forall \forall \exists }^{{\mathcal W}} B\ \Rightarrow \ A\succcurlyeq _{\forall \forall \exists }^{{\mathcal W}^0} B$$.
(ii) $A\succcurlyeq _{\exists \forall \forall }^{{\mathcal W}^0} B$ holds iff there exists $\alpha \in A$ such that for all $w\in {\mathcal W}^0$, $f_w(\alpha ) \ge \textit{Ut}_B(w)$.
(iii) $A\succcurlyeq _{\forall \forall \exists }^{{\mathcal W}^0} B$ holds iff for each $w\in {\mathcal W}^0$ there exists $\alpha \in A$ such that $f_w(\alpha ) \ge \textit{Ut}_B(w)$.

The second stage of the algorithm is another pre-processing step, whose correctness is shown by the following result, which easily follows using Proposition 5 and the definitions.

#### Lemma 9

(i) $A\succcurlyeq _{\forall \forall \exists }^{{\mathcal W}} B$ if and only if $\mathrm{UD}_{\mathcal W}(A) \succcurlyeq _{\forall \forall \exists }^{{\mathcal W}} \mathrm{UD}_{\mathcal W}(B)$.
(ii) Suppose that $A\succcurlyeq _{\forall \exists \forall }^{{\mathcal W}} C$. Then $A\succcurlyeq _{\forall \forall \exists }^{{\mathcal W}} B$ if and only if $A\succcurlyeq _{\forall \forall \exists }^{{\mathcal W}} B\setminus C$.

We describe below the three stages of our method for testing whether or not $A\succcurlyeq _{\forall \forall \exists }^{{\mathcal W}} B$ holds.

1. We use Proposition 22 to efficiently test (a) a necessary condition $A\succcurlyeq _{\forall \forall \exists }^{{\mathcal W}^0} B$, where ${\mathcal W}^0 = \textit{Ext}({\mathcal W})$ is the set of extreme points of ${\mathcal W}$; and (b) a sufficient condition, whether there exists $\alpha \in A$ such that for all $w\in {\mathcal W}^0$, $f_w(\alpha ) \ge \textit{Ut}_B(w)$; (the conditions can be tested together, by first computing $\textit{Ut}_B(w)$ for each $w\in {\mathcal W}^0$). If (a) is false then we know that $A\not \succcurlyeq _{\forall \forall \exists }^{{\mathcal W}} B$ (because of monotonicity with respect to ${\mathcal W}$); if (b) is true then we know that $A\succcurlyeq _{\forall \forall \exists }^{{\mathcal W}} B$ holds. If the necessary condition is false, or the sufficient condition is true, then we need go no further.
2. We perform a second pre-processing stage involving reducing the sets $A$ and $B$; this step has complexity proportional to $|A||B|$. We replace $A$ by $\mathrm{UD}_{\mathcal W}(A)$ and $B$ by $\mathrm{UD}_{\mathcal W}(B)$. We then eliminate all elements $\beta$ from $B$ such that for some $\alpha \in A$, $\alpha \succcurlyeq _{\mathcal W}\beta$. If $B$ becomes empty then we can stop, since we then have $A\succcurlyeq _{\forall \forall \exists }^{{\mathcal W}} B$.
3. We determine whether $A\succcurlyeq _{\forall \forall \exists }^{{\mathcal W}} B$ holds using one of the methods in Sects. 11.2 and 10, i.e., doing either (a), (b) or (c) below:
  - We check if $\textit{SMR}_{\mathcal W}(A, \{\beta \})\le 0$ for all $\beta \in B$ using linear programming, as described in Proposition 18 of Sect. 11.2.
  - Using Theorem 4(ii) of Sect. 10, i.e., testing if $\textit{Ext}(\Gamma ({\mathcal W},A))$ equals $\textit{Ext}(\Gamma ({\mathcal W},A\cup B))$.
  - Using Theorem 4(iii) of Sect. 10, i.e., testing if $f_w(\beta ) \le r$ for all $\beta \in B$ and for all $(w,r) \in \textit{Ext}(\Gamma ({\mathcal W},A))$.

The method can be easily adapted to compute $\textit{SMR}_{\mathcal W}(A, B)$. We cannot use the Stage (1) pre-processing, but the first part of Stage (2) can be used as pre-processing for the computation of $\textit{SMR}_{\mathcal W}(A, B)$, because $\textit{SMR}_{\mathcal W}(A, B) = \textit{SMR}_{\mathcal W}(\mathrm{UD}_{\mathcal W}(A), \mathrm{UD}_{\mathcal W}(B))$ by Lemma 8(ii). By Theorem 4(iv) we have that $\textit{SMR}_{\mathcal W}(A, {\{\beta \}})$ $= \max \{f_w(\beta ) - r$ $\, : \,(w,r) \in \textit{Ext}(\Gamma ({\mathcal W},A)) \}$. Thus, method (c) can be adapted to also compute $\textit{SMR}_{\mathcal W}(A, {\{\beta \}})$. Similarly method (a) can be adapted to compute $\textit{SMR}_{\mathcal W}(A, {\{\beta \}})$ for each $\beta \in B$. $\textit{SMR}_{\mathcal W}(A, B)$ can then be computed as $\max _{\beta \in B} \textit{SMR}_{\mathcal W}(A, {\{\beta \}})$ by Proposition 15. However, the implementation of the methods (a) and (c) for testing the dominance is slightly more efficient than the implementation for computing $\textit{SMR}_{\mathcal W}(A, B)$ since we can stop the execution as soon as we find a lower bound for $\textit{SMR}_{\mathcal W}(A, B)$ greater than zero, i.e., when we find $\beta \in B$ such that $\textit{SMR}_{\mathcal W}(A, \{\beta \})>0$ for method (a), and $\beta \in B$ and $(w,r) \in \textit{Ext}(\Gamma ({\mathcal W},A))$ such that $f_w(\beta ) > r$ for method (c).

Although we focus on non-strict dominance, the same algorithms can also be used to test the strong strict dominance $A\gg _{\forall \forall \exists }^{{\mathcal W}} B$ given as for all $w\in {\mathcal W}$, $\textit{Ut}_A(w) > \textit{Ut}_B(w)$. In particular, under the conditions of Theorem 4, we have $A\gg _{\forall \forall \exists }^{{\mathcal W}} B$ $\iff$ $\textit{SMR}_{\mathcal W}(A, B) < 0$, which is if and only if for all $(w,r) \in \textit{Ext}(\Gamma ({\mathcal W},A))$ and for all $\beta \in B$ we have $f_w(\beta ) < r$.

## Experimental testing

In this section we show some experimental results, and we analyse the computational cost of the procedures presented in Sect. 12 for filtering a set of alternatives maintaining equivalence and for testing the dominance between sets. We considered linear utility functions $f_w(\alpha ) = w\cdot \hat{\alpha }$ in all our experiments, with the set ${\mathcal W}$ of feasible scenarios $w$ being a subset of the unit $({p}-1)$-simplex defined by the intersection of $\rho$ randomly generated half-spaces representing input user preferences. Specifically, we choose $\rho$ (consistent) random user preferences of the form $aw_i+bw_j\ge cw_k$ (meaning that the user prefers *a* units of $w_i$ and *b* units of $w_j$ to *c* units of $w_k$), like in [41]. The sets $A$ and $B$ of utility vectors used in our experiments are randomly generated. See Appendix B for details about our random problem generator. All experiments were performed on computer facilitated by an Intel(R) Xeon(R) E5620 2.40 GHz processor with 32 GB of RAM. We used CPLEX 12.8 [35] as the linear programming solver, and we used the Python library pycddlib [61] for computing the extreme points of a convex polytope. CPLEX is an industrial tool highly optimised which has been commercialised for the first time more than 30 years ago and continuously improved. Pycddlib is a wrapper for the Komei Fukuda’s cddlib library [26] based on the *double description method* [27, 44]. It is worth noticing that the comparison of our algorithms may not be fair since pycddlib may not be as highly optimised as CPLEX. For example, we noticed that we could incur runtime exceptions related to precision issues if we do not approximate real numbers with fractions, and the use of fractions slowed down our algorithms up to 5 times for the CPLEX-based implementations and up to 15 times for the pycddlib-based implementations. We first show the experimental results for our main procedures as a whole, i.e., including the preliminary steps such us the $\mathrm{UD}_{\mathcal W}$ filtering. After that, we will show the performances of some specific operations.

For convenience of notation, denote $\mathrm{PSO}_{\mathcal W}(A)$ by $\mathrm{PSO}(A,{\mathcal W})$, $\mathrm{PO}_{\mathcal W}(A)$ by $\mathrm{PO}(A,{\mathcal W})$ and $\textit{SMR}_{\mathcal W}(A,B)$ by $\textit{SMR}(A,B,{\mathcal W})$.

### Comparison of algorithms for computing $\mathrm{PSO}(A,{\mathcal W})$

Here we describe some experimental results for the two methods presented in Sect. 12.1 to compute $\mathrm{PSO}(A,{\mathcal W})$; the first based on a linear programming solver ($\mathrm{PSO_{LP}}$) (see Sect. 12.1(a)), and the second based on the evaluation of the extreme points of the epigraph $\Gamma ({\mathcal W},A)$ ($\mathrm{PSO_{EP}}$) (see Sect. 12.1(b)). The results are an average over 100 experiments.

**Table 1:** Average execution time of the methods $\mathrm{PSO_{LP}}$ and $\mathrm{PSO_{EP}}$ for computing the minimal equivalent subset with respect to $\textit{dim}({\mathcal W})$. The last column shows the average size of the sets filtered with the $\mathrm{PSO}$ operator The experiments relates to randomly generated sets $A$ with size $|A|=100$ and $\rho =3$ randomly generated user preferences. The timings include the $\mathrm{UD}$ filtering on the input set

$\ DimW$	$\mathrm{PSO_{LP}}(A,{\mathcal W})$[s]	$\mathrm{PSO_{EP}}(A,{\mathcal W})$[s]	$|\mathrm{PSO}(A,{\mathcal W})|$
2	0.083	**0**.**045**	4.27
3	0.238	**0**.**164**	9.15
4	0.645	**0**.**641**	14.95
5	**1**.**109**	2.419	22.92
6	**1**.**964**	10.786	31.76
7	**2**.**726**	43.379	39.98

In Table 1 we show the performances of the methods $\mathrm{PSO_{LP}}$ and $\mathrm{PSO_{EP}}$ with respect to $\textit{dim}({\mathcal W})$ and $\rho =3$ randomly generated user preferences. As expected, increasing $\textit{dim}({\mathcal W})$ the computation time and the size of the output set increased. $\mathrm{PSO_{EP}}$ was faster than $\mathrm{PSO_{LP}}$ for $\textit{dim}({\mathcal W})\le 4$. However, $\mathrm{PSO_{LP}}$ scaled better with respect to $\textit{dim}({\mathcal W})$; in fact, with $\textit{dim}({\mathcal W})=7$, $\mathrm{PSO_{LP}}$ was around 1.5 times slower than its average execution time with $\textit{dim}({\mathcal W})=6$, on the other hand, $\mathrm{PSO_{EP}}$ was 4 times slower. This is presumably related to the exponential growth of the number of extreme points of the epigraph with respect to $\textit{dim}({\mathcal W})$ (see Table 5).

**Table 2:** Average execution time of the methods $\mathrm{PSO_{LP}}$ and $\mathrm{PSO_{EP}}$ for computing the minimal equivalent subset with respect to $\rho$. The last column shows the average size of the filtered sets. The experiments relates to randomly generated sets $A$ with size $|A|=100$ and $\dim ({\mathcal W})=4$. The timings include the $\mathrm{UD}$ filtering on the input set

$\rho$	$\mathrm{PSO_{LP}}(A,{\mathcal W})$	$\mathrm{PSO_{EP}}(A,{\mathcal W})$	$|\mathrm{PSO}(A,{\mathcal W})|$
0	**0**.**438**	0.743	22.86
3	0.648	**0**.**644**	14.98
6	0.543	**0**.**531**	10.02
9	**0**.**48**	0.484	7.92
12	**0**.**479**	0.502	6.89
15	**0**.**444**	0.465	4.97

In Table 2 we show the performances of the methods $\mathrm{PSO_{LP}}$ and $\mathrm{PSO_{EP}}$ with respect to the number of user preferences $\rho$ and considering fixed $\textit{dim}({\mathcal W})=4$. The two methods performed similarly under this configuration. As expected, by increasing the number of user preferences, the size of $\mathrm{PSO}(A,{\mathcal W})$ decreases, and the execution time tends to reduce.

**Fig. 2:**
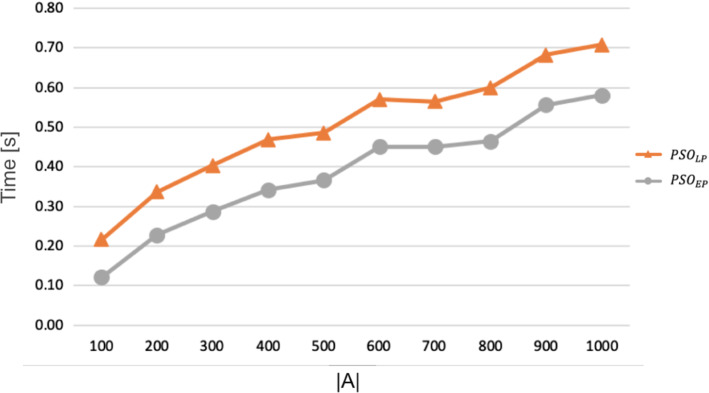
Average execution time in seconds of the methods $\mathrm{PSO_{LP}}$ (orange triangle) and $\mathrm{PSO_{EP}}$ (grey circle) to compute $\mathrm{PSO}(A,{\mathcal W})$ with respect to the initial set size of $A$, with $\textit{dim}({\mathcal W})=3$ and $\rho =0$. The timings include the $\mathrm{UD}$ filtering

In Fig. 2 we can see the execution time with $\textit{dim}({\mathcal W})=3$ and $\rho =0$ of the methods $\mathrm{PSO_{LP}}$ and $\mathrm{PSO_{EP}}$ with respect to the size of the input set $A$. As we can see, in our experiments the two methods scaled very roughly linearly in this setting.

#### Computational cost

Let $n=|A|$ be the size of the input set and $\rho$ the number of inequalities representing user preferences. The computational cost of $\mathrm{PSO_{LP}}$ is then $O(nC_{LP}(n+\rho ))$, where $C_{LP}(n+\rho )$ is the computational cost of the linear programming solver with $n+\rho$ constraints.

Let ${p}=\textit{dim}({\mathcal W})+1$ be the size of the utility vectors in $A$, $m=|\Gamma ^0({\mathcal W},A)|$ the number of extreme points of $\Gamma ({\mathcal W},A)$, and $C_{EP}(\Gamma ({\mathcal W},A))$ the computational cost of computing the extreme points of $\Gamma ({\mathcal W},A)$. For details regarding the computational cost of computing the extreme points of a convex polytope see, e.g., [24]. $\mathrm{PSO_{EP}}$ computes $E_{\mathcal W}^A(\alpha )$ for each $\alpha \in A$ that has a computational cost of $O(C_{EP}(\Gamma ({\mathcal W},A))+n m {p})$ since we first need to compute the extreme points of $\Gamma ({\mathcal W},A)$, and then for each $(w,r) \in \textit{Ext}(\Gamma ({\mathcal W},A)$ we need to check if we have that $w\in E_{\mathcal W}^A(\alpha )$ for each $\alpha \in A$, which can be done by checking if $w\cdot \alpha =r$. Once we have computed $E_{\mathcal W}^A(\alpha )$ for each $\alpha \in A$, we have to check if $E_{\mathcal W}^A(\alpha _1)\subseteq E_{\mathcal W}^A(\alpha _2)$ or $E_{\mathcal W}^A(\alpha _2)\subseteq E_{\mathcal W}^A(\alpha _1)$ for each couple $\alpha _1,\alpha _2\in A$. Assuming that $E_{\mathcal W}^A(\alpha _i)$ is an ordered set, this operation has a computational cost of $O(n^2 m {p})$, since $|E_{\mathcal W}^A(\alpha )|$ is *O*(*m*); however, if we suppose that the number $|E_{\mathcal W}^A(\alpha )|$ of extreme points associated with an element $\alpha$ is $\frac{1}{n}$ the number of extreme points of $\Gamma ({\mathcal W},A)$, i.e., $O(\frac{m}{n})$, the computational cost of this operation is reduced to $O(n m {p})$. Thus, the whole computational cost of $\mathrm{PSO_{EP}}$ can then be approximated as $O(C_{EP}(\Gamma ({\mathcal W},A))+n m {p})$.

### Algorithms comparison for testing $A\succcurlyeq _{\forall \forall \exists }^{{\mathcal W}} B$

In this section we show some experimental results for the three methods presented in Sect. 12.2 to determine whether the condition $A\succcurlyeq _{\forall \forall \exists }^{{\mathcal W}} B$ holds, for given sets $A$ and $B$ of alternatives. The first makes use of a linear programming solver ($\mathrm{T_{LP}}$) (Sect. 12.2(a)), the second compares the extreme point sets of the two epigraph $\Gamma ({\mathcal W},A)$ and $\Gamma ({\mathcal W},A\cup B)$ ($\mathrm{T_{EU}}$) (Sect. 12.2(b)), and the third evaluates the value function $f_w(\beta )=w\cdot \beta$ for all $\beta \in B$ and the the extreme points $w$ of the epigraph $\Gamma ({\mathcal W},A)$ ($\mathrm{T_{EE}}$) (Sect. 12.2(b)). In our experimental results, testing the necessary condition and the sufficient condition (Sect. 12.2(1)) and filtering out elements of $B$ dominated by elements of $A$ (Sect. 12.2(2)) was enough to evaluate $A\succcurlyeq _{\forall \forall \exists }^{{\mathcal W}} B$ for the majority of our experiments (see Sect. 13.4). Thus, to assess the performances of the methods (a), (b) and (c) of Sect. 12.2, we considered an average of 100 experiments where the necessary condition succeeded, the sufficient condition failed and the filtering of $B$ didn’t reduce its size to zero.

**Table 3:** Average execution time of the methods $\mathrm{T_{LP}}$, $\mathrm{T_{EU}}$ and $\mathrm{T_{EE}}$ for testing $A\succcurlyeq _{\forall \forall \exists }^{{\mathcal W}} B$ with respect to $\textit{dim}({\mathcal W})$. The last column shows the percentage of times that $A\succcurlyeq _{\forall \forall \exists }^{{\mathcal W}} B$ was true. The results relate to experiments with input set size $|A|=|B|=100$ and $\rho =3$ user preferences, where the necessary condition was true and the sufficient condition was false, and where the filtering did not reduce the size of $B$ to zero. The reported timings include testing the sufficient and the necessary condition, and the filtering of the input sets

$\textit{dim}({\mathcal W})$	$\mathrm{T_{LP}}$[s]	$\mathrm{T_{EU}}$[s]	$\mathrm{T_{EE}}$[s]	Dominance[%]
2	0.118	0.118	**0**.**112**	48%
3	**0**.**284**	0.306	0.287	60%
4	**0**.**794**	0.943	0.873	61%
5	**1**.**38**	2.176	1.936	64%
6	**1**.**922**	5.649	4.661	60%
7	**2**.**878**	18.194	16.579	71%

**Table 4:** Average execution time of the methods $\mathrm{T_{LP}}$, $\mathrm{T_{EU}}$ and $\mathrm{T_{EE}}$ for testing $A\succcurlyeq _{\forall \forall \exists }^{{\mathcal W}} B$ with respect to $\rho$. The last column shows the percentage of times that $A\succcurlyeq _{\forall \forall \exists }^{{\mathcal W}} B$ was true. The results relate to experiments with input set size $|A|=|B|=100$ and $\textit{dim}({\mathcal W})=4$, where necessary condition was true and the sufficient condition was false, and where the filtering did not reduce the size of $B$ to zero. The timings include testing the sufficient and the necessary condition, and the filtering of the input sets

$\rho$	$\mathrm{T_{LP}}$[s]	$\mathrm{T_{EU}}$[s]	$\mathrm{T_{EE}}$[s]	Dominance[%]
0	**0**.**172**	0.358	0.313	66%
3	**0**.**872**	1.015	0.956	70%
6	**0**.**945**	1.051	1.001	56%
9	**0**.**918**	1.029	0.973	64%
12	**0**.**96**	1.063	1.01	66%
15	**1**.**052**	1.162	1.102	60%

As we can see from Table 3 and Table 4, $\mathrm{T_{LP}}$ was in general the fastest method and scaled better, and $\mathrm{T_{EE}}$ performed slightly better than $\mathrm{T_{EU}}$. The percentage of dominance is on average above $50\%$. This means that given two input sets $A$ and $B$ generated with our random problem generator, $B$ is likely to be dominated by $A$ once the necessary condition is satisfied. This may not be surprising since the necessary condition is satisfied if and only if $\textit{Ut}_A(w)\ge \textit{Ut}_B(w)$ for all $w\in {\mathcal W}^0$.

**Fig. 3:**
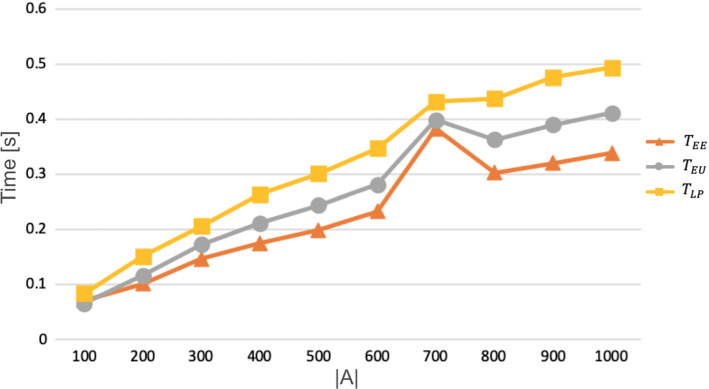
Average execution time in seconds of the methods $\mathrm{T_{LP}}$ (yellow square), $\mathrm{T_{EU}}$ (grey circle) and $\mathrm{T_{EE}}$ (orange triangle) to evaluate $A\succcurlyeq _{\forall \forall \exists }^{{\mathcal W}} B$ with respect to the initial set size of $A$, with $\textit{dim}({\mathcal W})=3$ and $\rho =0$. The timings include the $\mathrm{UD}$ filtering

**Fig. 4:**
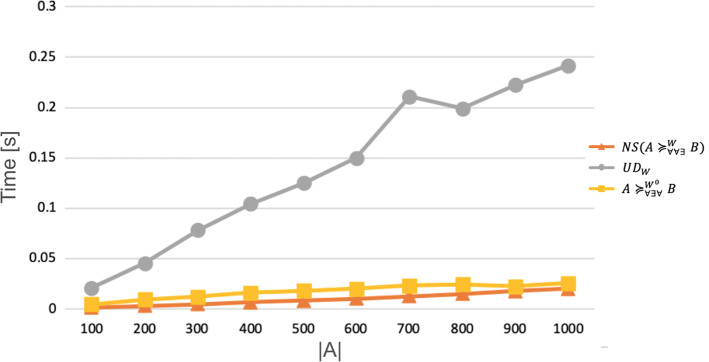
Average execution time in seconds of the $\mathrm{UD}_{{\mathcal W}}$ filtering (grey circle), the $A\succcurlyeq _{\forall \exists \forall }^{{\mathcal W}^0} B$ filtering (yellow square), and testing the necessary condition and sufficient condition of $A\succcurlyeq _{\forall \forall \exists }^{{\mathcal W}} B$ (orange triangle) with $\rho =0$ and $\textit{dim}({\mathcal W})=4$ with respect to the size of the input sets $A$ and $B$

In Fig. 3 we can see the execution time with $\textit{dim}({\mathcal W})=3$ and $\rho =0$ of the methods $\mathrm{T_{LP}}$, $\mathrm{T_{EU}}$ and $\mathrm{T_{EE}}$ with respect to the size of the input set $A$. As we can see, in our experiments the two methods scaled roughly linearly when fixing $\textit{dim}({\mathcal W})$ and $\rho$. The peak for $|A|=700$ is due to the randomness of the experiments; in this case, the $\mathrm{UD}$ filtering was on average less effective in reducing the size of $A$. We can see the same anomaly in Fig. 4 which reports the timing of the $\mathrm{UD}$ filtering.

#### Computational cost

The computational cost of $\mathrm{T_{LP}}$ is $O(nC_{LP}(T+n))$, where $C_{LP}(T+n)$ is the computational cost of the linear programming solver with $T+n$ constraints. Let $m_A=|\Gamma ^0({\mathcal W},A)|$ and $m_{A\cup B}=|\Gamma ^0({\mathcal W},A\cup B)|$ be the number of extreme points of $\Gamma ({\mathcal W},A)$ and $\Gamma ({\mathcal W},A\cup B)$, and let $C_{EP}(\Gamma ({\mathcal W},A))$ and $C_{EP}(\Gamma ({\mathcal W},A\cup B))$ be the computational cost of computing the extreme points of $\Gamma ({\mathcal W},A)$ and $\Gamma ({\mathcal W},A\cup B)$. The computational cost of $\mathrm{T_{EE}}$ is then $O(C_{EP}(\Gamma ({\mathcal W},A))+n m_Ap)$, and supposing that the sets $\textit{Ext}(\Gamma ({\mathcal W},A))$ and $\textit{Ext}(\Gamma ({\mathcal W},A\cup B))$ are ordered, the computational cost of $\mathrm{T_{EU}}$ is $O(C_{EP}(\Gamma ({\mathcal W},$ $A\cup B))+m_{A\cup B} p)$. For a generic evaluation of the computational cost of the above methods, we are supposing $A=B=n$, but the size of $B$ can be much smaller than the size of $A$ (see Table 7 and Table 8).

### Computation of the extreme points

For the preliminaries step we need first to compute the extreme points ${\mathcal W}^0$ of the convex polytope ${\mathcal W}$. The extreme points of the epigraph $\Gamma ({\mathcal W},A)$ and $\Gamma ({\mathcal W},B)$ are instead computed after the preliminaries step for the methods $\mathrm{T_{EU}}(A, B, {\mathcal W})$ and $\mathrm{T_{EE}}(A, B, {\mathcal W})$ to test the dominance and the method $\mathrm{PSO_{EP}}(A,{\mathcal W})$ to compute the minimal equivalent subset.

**Table 5:** Average number of extreme points ($\text {EP}$) of ${\mathcal W}$ and of the epigraph $\Gamma ({\mathcal W},A)$ and corresponding computational time with respect to $\textit{dim}({\mathcal W})$, with $|A|=100$ and $\pi =3$

$\textit{dim}({\mathcal W})$	$\#\text {EP}$ ${\mathcal W}$	$\#\text {EP}$ $\Gamma ({\mathcal W},A)$	time[s] $\text {EP}$ ${\mathcal W}$	time[s] $\text {EP}$ $\Gamma ({\mathcal W},A)$
2	4.34	10.62	0.0015	0.004
3	6.88	41.01	0.0021	0.0165
4	8.89	132.35	0.0026	0.0674
5	10.23	448.42	0.003	0.3127
6	11.34	1464.34	0.0037	1.495
7	12.63	4532.4	0.0043	6.5583

**Table 6:** Average number of extreme points ($\text {EP}$) of ${\mathcal W}$ and of the epigraph $\Gamma ({\mathcal W},A)$ and corresponding computational time with respect to the number of user preferences $\pi$, with $|A|=100$ and $\textit{dim}({\mathcal W})=4$

$\rho$	$\#\text {EP}$ ${\mathcal W}$	$\#\text {EP}$ $\Gamma ({\mathcal W},A)$	time[s] $\text {EP}$ ${\mathcal W}$	time[s] $\text {EP}$ $\Gamma ({\mathcal W},A)$
0	5.0	198.55	0.0012	0.1028
3	8.77	129.9	0.0026	0.0642
6	16.08	99.95	0.0052	0.0496
9	19.87	85.78	0.008	0.0444
12	23.19	81.39	0.0117	0.0471
15	25.94	64.67	0.015	0.0406

Tables 5 and 6 show the number of extreme points of ${\mathcal W}$, the number of extreme points of the epigraph $\Gamma ({\mathcal W},A)$ and the corresponding computational time with respect to the dimension of ${\mathcal W}$ (Table 5) and the number of user preferences (Table 6). The results are an average of 100 instances with three randomly generated user preferences for the results in Table 5, and $\textit{dim}({\mathcal W})=5$ for Table 6. The number of extreme points and the computational time with respect to $\textit{dim}({\mathcal W})$ increase roughly linearly for ${\mathcal W}$ and exponentially for $\Gamma ({\mathcal W},A)$. Each user preference is a half-space with a corresponding hyper-plane that may redefine the boundaries of the convex polytope ${\mathcal W}$, thus increasing the number of extreme points of ${\mathcal W}$ and then the corresponding computational time. On the other hand, the number of extreme points of the epigraph and the corresponding computational time both decrease, as one increases the number of user preferences (Table 6). This is because the user preferences reduce the hyper-volume of ${\mathcal W}$; therefore, since the epigraph is built over ${\mathcal W}$, the intersection of ${\mathcal W}$ with the half-spaces representing the user preferences can exclude some of the extreme points of the epigraph.

For details regarding the computational cost of computing the extreme points of a convex polytope see, e.g., [24].

### Preliminaries step

**Table 7:** Average execution time in seconds for testing the necessary condition and the sufficient condition of $A\succcurlyeq _{\forall \forall \exists }^{{\mathcal W}} B$ ($\mathrm{NSc}$), the $\mathrm{UD}_{{\mathcal W}}$ filtering and the $A\succcurlyeq _{\forall \exists \forall }^{{\mathcal W}^0} B$ filtering ($\mathrm{Filt_{\forall \exists \forall }}$), and number of elements of $A'=\mathrm{UD}_{{\mathcal W}}(A)$, $B'=\mathrm{UD}_{{\mathcal W}}(B)$ and $B''= {\{\beta \in B': \forall \alpha \in A, \alpha \not \succcurlyeq _{\mathcal W}\beta \}}$ with $\rho =3$ and $|A|=|B|=100$ with respect to $\textit{dim}({\mathcal W})$

$$\textit{dim}({\mathcal W})$$	$$\mathrm{NSc}$$[s]	$$\mathrm{UD}_{{\mathcal W}}$$[s]	$$\mathrm{Filt_{\forall \exists \forall }}$$[s]	$$ |A'|$$	$$ |B'|$$	$$|B''|$$
2	0.013	0.084	0.007	8.0	6.56	2.37
3	0.018	0.202	0.041	16.0	14.03	4.01
4	0.025	0.559	0.161	28.05	23.33	8.89
5	0.025	0.889	0.357	41.28	36.21	14.8
6	0.024	1.189	0.52	55.3	46.72	24.28
7	0.026	1.614	0.901	67.35	57.43	28.66

**Table 8:** Average execution time in seconds for testing the necessary condition and the sufficient condition of $A\succcurlyeq _{\forall \forall \exists }^{{\mathcal W}} B$ ($\mathrm{NSc}$), the $\mathrm{UD}_{{\mathcal W}}$ filtering and the $A\succcurlyeq _{\forall \exists \forall }^{{\mathcal W}^0} B$ filtering ($\mathrm{Filt_{\forall \exists \forall }}$), and number of elements of $A'=\mathrm{UD}_{{\mathcal W}}(A)$, $B'=\mathrm{UD}_{{\mathcal W}}(B)$ and $B''= {\{\beta \in B': \forall \alpha \in A, \alpha \not \succcurlyeq _{\mathcal W}\beta \}}$ with $\textit{dim}({\mathcal W})=4$ and $|A|=|B|=100$ with respect to the number of user preferences

$$\rho $$	$$\mathrm{NSc}$$[s]	$$\mathrm{UD}_{{\mathcal W}}$$[s]	$$\mathrm{Filt_{\forall \exists \forall }}$$[s]	$$ |A'|$$	$$ |B'|$$	$$|B''|$$
0	0.002	0.044	0.014	44.07	35.93	15.91
3	0.026	0.616	0.173	28.77	24.63	7.86
6	0.05	0.694	0.161	19.02	16.82	5.69
9	0.076	0.7	0.112	14.29	10.94	3.39
12	0.095	0.752	0.08	11.44	8.05	3.08
15	0.114	0.822	0.079	10.14	7.79	3.0

The preliminary steps are the operations executed on the input sets before testing the dominance $A\succcurlyeq _{\forall \forall \exists }^{{\mathcal W}} B$ or computing the $\text {SME}$, i.e., testing the necessary and the sufficient condition (Sect. 12.2(1)), and the $\succcurlyeq _{\forall \exists \forall }^{{\mathcal W}^0}$ filtering (Sect. 12.2(2)) for the dominance, and the $\mathrm{UD}_{{\mathcal W}}$ filtering for both dominance and $\text {SME}$. In Table 8, Table 7 and Fig. 4, we show some experimental results of the preliminary steps. The results are an average of 100 experiments in which the necessary condition is true, the sufficient condition is False, and the $\succcurlyeq _{\forall \exists \forall }^{{\mathcal W}^0}$ filtering did not reduce the size of $B$ to zero.

Testing the necessary and the sufficient condition was the fastest operation performed before evaluating $A\succcurlyeq _{\forall \forall \exists }^{{\mathcal W}}B$. The computational cost of this operation is $O(n|{\mathcal W}^0|{p})$. The necessary condition failed or the sufficient condition succeeded in most experiments, allowing the algorithm to stop early. This happened from $98.5\%$ to $94\%$ (depending on $\textit{dim}({\mathcal W})$) of the random problems generated with the set-up of the experiments of Table 7, and from $96\%$ to $99.2\%$ (depending on the number of user preferences) of the random problems generated with the set-up of the experiments of Table 8. Therefore, since the execution time was a small fraction of the time spent by $\mathrm{UD}_{{\mathcal W}}$ (see Table 7 and Table 8), it looks like that this is a very worthwhile check for testing $A\succcurlyeq _{\forall \forall \exists }^{{\mathcal W}} B$.

We used the $\mathrm{UD}_{{\mathcal W}}$ filtering to reduce the size of the input sets $A$ and $B$ before evaluating $\mathrm{UD}_{{\mathcal W}}(A) \succcurlyeq _{\forall \exists \forall }^{{\mathcal W}^0} \mathrm{UD}_{{\mathcal W}}(B)$ when testing the dominance, and to reduce the size of the input set $A$ before computing $\mathrm{PSO}_{{\mathcal W}}(A)$. The computational cost of this operation is $O(n^2|{\mathcal W}^0|{p})$ and it seems to speed up the overall execution for both testing $A\succcurlyeq _{\forall \forall \exists }^{{\mathcal W}} B$ and computing $\mathrm{PSO}_{{\mathcal W}}(A)$. For example, the $\mathrm{UD}_{{\mathcal W}}$ filtering on 100 randomly generated input set $A$ with $|A|=100$, $\textit{dim}({\mathcal W})=4$ and three user preferences, reduced the input set size to an average of 24 elements with an average execution time of 0.3 s. The execution time of $\mathrm{PSO}_{{\mathcal W}}(A)$ over the same experimental set-up with and without the input set $A$ filtered by $\mathrm{UD}_{{\mathcal W}}$ was on average 0.37 and 0.89 s respectively for $\mathrm{PSO_{LP}}$ and 0.38 and 1.1 s for $\mathrm{PSO_{EP}}$.

The $\succcurlyeq _{\forall \exists \forall }^{{\mathcal W}^0}$ filtering is part of the pre-processing to further reduce the size of $B$ after the $\mathrm{UD}_{{\mathcal W}}$ filtering and before evaluating the dominance. The computational cost of this operation is $O(n^2|{\mathcal W}^0|{p})$. This filtering improved the overall execution time of our experiments since it reduced the size of the set $\mathrm{UD}_{{\mathcal W}}(B)$ by more than half in average (see $|B'|$ and $|B''|$ of Table 7 and Table 8). For example, the $\succcurlyeq _{\forall \exists \forall }^{{\mathcal W}^0}$ filtering on 100 randomly generated input set filtered by $\mathrm{UD}_{{\mathcal W}}$ filtering, with initial input sets size $|A|=|B|=100$, $\textit{dim}({\mathcal W})=4$ and three user preferences, reduced the average size of $\mathrm{UD}_{{\mathcal W}}(B)$ from 23.46 to 8.8 elements with an average execution time of 0.17 s. The execution time of testing testing $A\succcurlyeq _{\forall \forall \exists }^{{\mathcal W}} B$ over the same experimental set-up with and without the set $\mathrm{UD}_{{\mathcal W}}(B)$ filtered by the $\succcurlyeq _{\forall \exists \forall }^{{\mathcal W}^0}$ filtering was on average 0.04 and 0.17 s respectively for $\mathrm{T_{LP}}$, 0.18 and 0.21 s for $\mathrm{T_{EU}}$, and 0.12 and 0.24 s for $\mathrm{T_{EE}}$. In some of the experiments with the necessary condition true and the sufficient condition false, the $\succcurlyeq _{\forall \exists \forall }^{{\mathcal W}^0}$ filtering has been enough for testing $A\succcurlyeq _{\forall \forall \exists }^{{\mathcal W}} B$ since it reduced the size of $B$ to zero. This happened from $22.5\%$ to $1\%$ (depending on $\textit{dim}({\mathcal W})$) of the random problems generated for the results in Table 8, and from $18\%$ to $11.5\%$ (depending on the number of user preferences) of the random problems generated for the results in Table 7.

The $\mathrm{UD}_{{\mathcal W}}$ filtering and the $\succcurlyeq _{\forall \exists \forall }^{{\mathcal W}^0}$ filtering can be executed in any order. However, the overall execution time of our experiments was faster executing first the $\mathrm{UD}_{{\mathcal W}}$ filtering. This may be because the $A\succcurlyeq _{\forall \exists \forall }^{{\mathcal W}^0}B$ filtering compares every element of $B$ with every element of $A$ in the worst case, and thus there may be several redundant comparisons if $A\ne \mathrm{UD}_{{\mathcal W}}(A)$ since for $\alpha ,\beta \in A$ with $\alpha \succcurlyeq _{\forall \exists \forall }^{{\mathcal W}^0}\beta$ we have that if $\beta \succcurlyeq _{\forall \exists \forall }^{{\mathcal W}^0}\gamma$ with $\gamma \in B$, then $\alpha \succcurlyeq _{\forall \exists \forall }^{{\mathcal W}^0}\gamma$. As we can see in Fig. 3, the $\succcurlyeq _{\forall \exists \forall }^{{\mathcal W}^0}$ filtering seems to scale better and be faster than the $\mathrm{UD}_{{\mathcal W}}$ filtering, but this is because it is executed after the $\mathrm{UD}_{{\mathcal W}}$ filtering. In fact, the $\mathrm{UD}_{{\mathcal W}}$ filtering would be faster than the $\succcurlyeq _{\forall \exists \forall }^{{\mathcal W}^0}$ filtering if the filtering order was inverted.

### $\mathrm{PO}(A,{\mathcal W})$ and $\textit{SMR}(A,B,{\mathcal W})$

Here we describe some experimental results for the computation of the set of possibly optimal alternatives $\mathrm{PO}(A,{\mathcal W})$ and the setwise max regret $\textit{SMR}_{\mathcal W}(A,$ $B,{\mathcal W})$ using well-known methods based on linear programming ($\mathrm{PO_{LP}}(A,{\mathcal W})$, see 12.1(a), and $\textit{SMR}_{LP}(A,B,{\mathcal W})$, see 12.2(3)(a)) and our novel methods based on the extreme points of the epigraph ($\mathrm{PO_{EP}}(A,{\mathcal W})$, see 12.1(b), and $\textit{SMR}_{EP}(A,B,{\mathcal W})$, see 12.2(3)(c)).

In Tables 9 and 10 we show some experimental results for the computation of $\mathrm{PO}(A,{\mathcal W})$ with respect to $\textit{dim}({\mathcal W})$ and the number $\rho$ of user preferences. Our method $\mathrm{PO_{EP}}(A,{\mathcal W})$ performed better for $\textit{dim}({\mathcal W})\le 4$. However, $\mathrm{PO_{LP}}(A,{\mathcal W})$ scaled better with respect to $\textit{dim}({\mathcal W})$. This may be because of the exponential grow of the number of extreme points of the epigraph with respect to $\textit{dim}({\mathcal W})$.

**Table 9:** Average execution time of the methods $\mathrm{PO_{LP}}$ and $\mathrm{PO_{EP}}$ for computing the minimal equivalent subset with respect to $\textit{dim}({\mathcal W})$. The last column shows the average size of the filtered sets. The experiments relates to randomly generated sets $A$ with size $|A|=100$ and $\rho =3$ randomly generated user preferences. The timings include the $\mathrm{UD}$ filtering on the input set

$\ DimW$	$\mathrm{PO_{LP}}(A,{\mathcal W})$[s]	$\mathrm{PO_{EP}}(A,{\mathcal W})$[s]	$|\mathrm{PO}(A,{\mathcal W})|$
2	0.084	**0**.**042**	4.37
3	0.243	**0**.**128**	9.23
4	0.67	**0**.**439**	15.16
5	**1**.**177**	1.309	23.49
6	**2**.**067**	5.148	32.57
7	**2**.**889**	18.927	41.27

Bold values highlight the best average timings

**Table 10:** Average execution time of the methods $\mathrm{PO_{LP}}$ and $\mathrm{PO_{EP}}$ with respect to the number of user preferences $\rho$. The last column shows the average size of the filtered sets. The experiments relates to randomly generated sets $A$ with size $|A|=100$ and $\textit{dim}({\mathcal W})=4$. The timings include the $\mathrm{UD}$ filtering on the input set

$\rho$	$\mathrm{PO_{LP}}(A,{\mathcal W})$	$\mathrm{PO_{EP}}(A,{\mathcal W})$	$|\mathrm{PO}(A,{\mathcal W})|$
0	0.428	**0**.**358**	23.36
3	0.675	**0**.**436**	15.32
6	0.557	**0**.**412**	10.14
9	0.492	**0**.**395**	7.97
12	0.487	**0**.**422**	7.0
15	0.448	**0**.**415**	4.99

Bold values highlight the best average timings

**Fig. 5:**
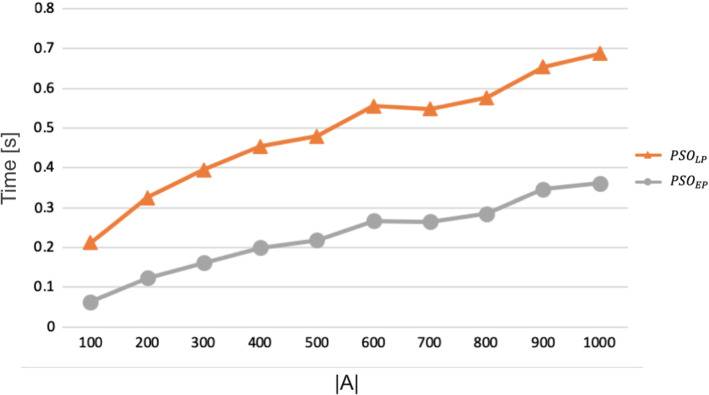
Average execution time in seconds of the methods $\mathrm{PO_{LP}}$ (orange triangle) and $\mathrm{PO_{EP}}$ (grey circle) to compute $\mathrm{PO}(A,{\mathcal W}$ with respect to the initial set size of $A$, with $\textit{dim}({\mathcal W})=3$ and $\rho =0$. The timings include the $\mathrm{UD}$ filtering

In Fig. 5 we show how $\mathrm{PO_{LP}}(A,{\mathcal W})$ and $\mathrm{PO_{EP}}(A,{\mathcal W})$ scaled with respect to the size of the input sets. The timings include the $\mathrm{UD}$ filtering and it seems that the overall execution time scaled very roughly linearly.

Table 11 and Table 12 show the execution time of our experiments for the computation of $\textit{SMR}(A,B,{\mathcal W})$ with $B\subseteq A$ with respect to $\textit{dim}({\mathcal W})$ and the size of $B$. Table 11 shows the timings of $\textit{SMR}_{LP}(A,B,{\mathcal W})$ and Table 12 those of $\textit{SMR}_{EP}(A,B,{\mathcal W})$. Also in this case the method based on linear programming scaled better. However, $\textit{SMR}_{EP}(A,B,{\mathcal W})$ was in average the fastest.

**Table 11:** Average execution time in seconds to compute $\textit{SMR}_{LP}(A,B,{\mathcal W})$ using the linear programming solver, varying $\textit{dim}({\mathcal W})$ and $|B|$. $A$ is a set of 100 undominated elements and $B\subset A$. $A$ and $B$ are randomly generated for each repetition of the experiment

	$|B|=2$	$|B|=3$	$|B|=4$	$|B|=5$	$|B|=6$
$\textit{dim}({\mathcal W})=1$	0.671	0.680	0.708	0.699	0.737
$\textit{dim}({\mathcal W})=2$	0.707	0.705	0.719	0.718	0.730
$\textit{dim}({\mathcal W})=3$	0.688	0.697	0.713	0.736	0.753
$\textit{dim}({\mathcal W})=4$	0.714	0.726	0.773	0.760	0.771
$\textit{dim}({\mathcal W})=5$	0.713	0.742	0.752	0.764	0.782
$\textit{dim}({\mathcal W})=6$	0.729	0.739	0.765	0.794	**0**.**806**

Bold values highlight better average timings with respect to the results of Table 12

**Table 12:** Average execution time in seconds to compute $\textit{SMR}_{EP}(A,B,{\mathcal W})$ using the extreme points of the epigraph, varying $\textit{dim}({\mathcal W})$ and $|B|$. $A$ is a set of 100 undominated elements and $B\subset A$. $A$ and $B$ are randomly generated for each repetition of the experiment

	$|B|=2$	$|B|=3$	$|B|=4$	$|B|=5$	$|B|=6$
$\textit{dim}({\mathcal W})=1$	**0**.**012**	**0**.**014**	**0**.**016**	**0**.**016**	**0**.**018**
$\textit{dim}({\mathcal W})=2$	**0**.**025**	**0**.**034**	**0**.**042**	**0**.**048**	**0**.**054**
$\textit{dim}({\mathcal W})=3$	**0**.**042**	**0**.**066**	**0**.**092**	**0**.**117**	**0**.**141**
$\textit{dim}({\mathcal W})=4$	**0**.**072**	**0**.**121**	**0**.**177**	**0**.**236**	**0**.**299**
$\textit{dim}({\mathcal W})=5$	**0**.**105**	**0**.**194**	**0**.**317**	**0**.**427**	**0**.**602**
$\textit{dim}({\mathcal W})=6$	**0**.**153**	**0**.**290**	**0**.**511**	**0**.**788**	1.085

Bold values highlight better average timings with respect to the results of Table 11

## Discussion

We defined natural notions of equivalence and dominance for a general model of sets of multi-attribute utility, and proved general properties. Computationally we focused especially on the linear (weighted sum) case and we proved that there is a unique setwise-minimal equivalent subset of any (equivalence-free) set of utility vectors $A$. This set then equals the set of possibly strictly optimal alternatives $\mathrm{PSO}(A)$, and is a compact representation of the utility function for $A$, giving the utility achievable with $A$ for each scenario. We show that filtering a query with the $\mathrm{PSO}$ operator avoids the potential of inconsistency in the user response. Along with pre-processing techniques we developed a linear programming method for generating $\mathrm{PSO}(A)$, and a method based on computing the extreme points of the epigraph of the utility function (EEU), as well as related methods for testing dominance. We implemented the approaches and our testing on random problems showed that both methods scaled to substantially sized problems, with the EEU method being better for lower dimensions. Our methods can be directly applied to reduce the set of utility vectors derived for a multi-objective influence diagram [41] or a multi-objective optimisation problem [42].

Our experimental testing assumed inputs in which the sets $A$ are represented explicitly. In some situations, the sets are more naturally represented combinatorially, as a set of constraints or a SAT formula. However, the fundamental properties that are the bases of our algorithms still apply, and these can enable the methods to be adapted for the linear convex case. Specifically, the minimal equivalent set corresponds with the set of possibly strictly optimal elements, and $\mathrm{PSO}_{\mathcal W}$ is equal to $\mathrm{MPO}_{\mathcal W}$ and satisfies Path Independence (by Proposition 8 and Corollaries 2 and 3). In particular, it would interesting to explore the use of AND/OR Branch-and-Bound algorithms, similar to those used for calculating the possibly optimal alternatives in [72]. The fact that $\mathrm{PSO}_{\mathcal W}$ satisfies Path Independence and translation invariance means that it satisfies the additive decomposition property (see Proposition 1 of [72]), which is fundamental for the AND/OR B &B algorithms.

A further natural application of our model and methods is for computing the Value of Information [23] for a multi-objective influence diagram. Each observable variable generates a Value of Information function which is a utility function $\textit{Ut}_A$, so different observable variables can be compared using the relation $\succcurlyeq _{\forall \forall \exists }^{{\mathcal W}}$.

Although we focus especially on the case where $f_w(\alpha )$ is linear in $w$, which covers a wide range of important preference models, it would also be interesting to develop computational procedures for non-linear cases (such as quadratic utility models) based on our more general characterisation results, such as Theorems 3 and 4.

## A proofs appendix

This appendix includes all the proofs of the results in the paper that do not appear in the main body of the paper, and includes also auxiliary lemmas that are used to prove these results.

### Results in Sect. 3

For the convenience of the reader, we recall the definitions of the three dominance relations from Definition 3. Consider any ${\mathcal W}\subseteq {\mathcal U}$ and $A, B\in {\mathcal M}$.

- $A\succcurlyeq _{\forall \forall \exists }^{{\mathcal W}} B$ holds if and only if for all $\beta \in B$ and for all $w\in {\mathcal W}$ there exists $\alpha \in A$ such that $\alpha \succcurlyeq _w\beta$.
- $A\succcurlyeq _{\forall \exists \forall }^{{\mathcal W}} B$ holds if and only if for all $\beta \in B$ there exists $\alpha \in A$ such that for all $w\in {\mathcal W}$, $\alpha \succcurlyeq _w\beta$.
- $A\succcurlyeq _{\exists \forall \forall }^{{\mathcal W}} B$ holds if and only if there exists $\alpha \in A$ such that for all $\beta \in B$ and for all $w\in {\mathcal W}$, $\alpha \succcurlyeq _w\beta$.

#### Proposition 1

For any ${\mathcal W}\subseteq {\mathcal U}$ we have $\succcurlyeq _{\exists \forall \forall }^{{\mathcal W}}$ $\subseteq$ $\succcurlyeq _{\forall \exists \forall }^{{\mathcal W}}$ $\subseteq$ $\succcurlyeq _{\forall \forall \exists }^{{\mathcal W}}$. Also, $\succcurlyeq _{\mathcal W}$ and each of the relations, $\succcurlyeq _{\forall \forall \exists }^{{\mathcal W}}$,

$\succcurlyeq _{\forall \exists \forall }^{{\mathcal W}}$ and $\succcurlyeq _{\exists \forall \forall }^{{\mathcal W}}$, is transitive. Furthermore, we have the following chaining properties:

(i) If $A\succcurlyeq _{\forall \exists \forall }^{{\mathcal W}} B$ and $B\succcurlyeq _{\exists \forall \forall }^{{\mathcal W}} C$ then $A\succcurlyeq _{\exists \forall \forall }^{{\mathcal W}} C$.
(ii) If $\succcurlyeq _{*}^{{\mathcal W}}$ is any of the relations $\succcurlyeq _{\forall \forall \exists }^{{\mathcal W}}$, $\succcurlyeq _{\forall \exists \forall }^{{\mathcal W}}$ or $\succcurlyeq _{\exists \forall \forall }^{{\mathcal W}}$ then $A\succcurlyeq _{\exists \forall \forall }^{{\mathcal W}} B$ and $B\succcurlyeq _{*}^{{\mathcal W}} C$ implies $A\succcurlyeq _{\exists \forall \forall }^{{\mathcal W}} C$.

#### Proof

The nesting properties $\succcurlyeq _{\exists \forall \forall }^{{\mathcal W}}$ $\subseteq$ $\succcurlyeq _{\forall \exists \forall }^{{\mathcal W}}$ $\subseteq$ $\succcurlyeq _{\forall \forall \exists }^{{\mathcal W}}$ follow easily from the definitions.

(i): Assume that $A\succcurlyeq _{\forall \exists \forall }^{{\mathcal W}}B$ and $B\succcurlyeq _{\exists \forall \forall }^{{\mathcal W}}C$. Since $B\succcurlyeq _{\exists \forall \forall }^{{\mathcal W}}C$, there exists an alternative $\beta$ in B such that for all $\gamma \in C$, $\beta \succcurlyeq _{\mathcal W}\gamma$. Since $A\succcurlyeq _{\forall \exists \forall }^{{\mathcal W}} B$, there exists $\alpha \in A$ such that $\alpha \succcurlyeq _{\mathcal W}\beta$. Since $\succcurlyeq _{\mathcal W}$ is transitive and $\alpha \succcurlyeq _{\mathcal W}\beta$ and $\beta \succcurlyeq _{\mathcal W}\gamma$ for all $\gamma \in C$, then $\alpha \succcurlyeq _{\mathcal W}\gamma$ for all $\gamma \in C$, which implies $A\succcurlyeq _{\exists \forall \forall }^{{\mathcal W}} C$.

(ii): By the nesting properties it follows that $B\succcurlyeq _{\forall \exists \forall }^{{\mathcal W}} C$ implies $B\succcurlyeq _{\forall \forall \exists }^{{\mathcal W}} C$ and $B\succcurlyeq _{\exists \forall \forall }^{{\mathcal W}} C$ implies $B\succcurlyeq _{\forall \forall \exists }^{{\mathcal W}} C$. Thus, if (ii) is true when $\succcurlyeq$ equals $\succcurlyeq _{\forall \forall \exists }^{{\mathcal W}}$, then it is true also for the other two cases. We then need to prove that $A\succcurlyeq _{\exists \forall \forall }^{{\mathcal W}} B$ and $B\succcurlyeq _{\forall \forall \exists }^{{\mathcal W}} C$ implies $A\succcurlyeq _{\exists \forall \forall }^{{\mathcal W}} C$: since there exists $\alpha \in A$ such that for all $\beta \in B$ $\alpha \succcurlyeq _{\mathcal W}\beta$ ($A\succcurlyeq _{\exists \forall \forall }^{{\mathcal W}} B$), and for all $\gamma \in C$ there exists $\beta \in B$ such that $\beta \succcurlyeq _{\mathcal W}\gamma$ ($B\succcurlyeq _{\forall \forall \exists }^{{\mathcal W}}C$), then, by the transitivity of $\succcurlyeq _{\mathcal W}$, there exists $\alpha \in A$ such that such that $\alpha \succcurlyeq _{\mathcal W}\gamma$ for all $\gamma \in C$, i.e., $A\succcurlyeq _{\exists \forall \forall }^{{\mathcal W}} C$.

Transitivity of $\succcurlyeq _{\exists \forall \forall }^{{\mathcal W}}$ is implied by (ii).

Transitivity of $\succcurlyeq _{\forall \forall \exists }^{{\mathcal W}}$: we have transitivity of $\succcurlyeq _{\exists \forall \forall }^{{\{w\}}}$, which is the same as $\succcurlyeq _{\forall \forall \exists }^{{\{w\}}}$, and thus transitivity of $\succcurlyeq _{\forall \forall \exists }^{{\mathcal W}}$, as the intersection of an arbitrary set of transitive relations is transitive.

Transitivity of $\succcurlyeq _{\forall \exists \forall }^{{\mathcal W}}$: Suppose $A\succcurlyeq _{\forall \exists \forall }^{{\mathcal W}} B$ and $B\succcurlyeq _{\forall \exists \forall }^{{\mathcal W}} C$ and consider any $\gamma \in C$. Because $B\succcurlyeq _{\forall \exists \forall }^{{\mathcal W}} C$, there exists $\beta \in B$ such $\beta \succcurlyeq _{\mathcal W}\gamma$. $A\succcurlyeq _{\forall \exists \forall }^{{\mathcal W}} B$ implies there exists $\alpha \in A$ such that $\alpha \succcurlyeq _{\mathcal W}\beta$, and thus for the transitivity of $\succcurlyeq _{\mathcal W}$, $\alpha \succcurlyeq _{\mathcal W}\gamma$ for all $\gamma \in C$, proving that $A\succcurlyeq _{\forall \exists \forall }^{{\mathcal W}}C$. $\square$

#### Proposition 2

For all $A,B\in {\mathcal M}$, $A\succcurlyeq _{\forall \forall \exists }^{{\mathcal W}} B$ $\iff$ $A\equiv _{\forall \forall \exists }^{{\mathcal W}} A\cup B$; and $A\succcurlyeq _{\forall \exists \forall }^{{\mathcal W}} B$ $\iff$ $A\equiv _{\forall \exists \forall }^{{\mathcal W}} A\cup B$.

#### Proof

We prove the result for $\succcurlyeq _{\forall \forall \exists }^{{\mathcal W}}$; the result for $\succcurlyeq _{\forall \exists \forall }^{{\mathcal W}}$ follows in exactly the same way. The definition easily implies that $A\cup B\succcurlyeq _{\forall \forall \exists }^{{\mathcal W}} A$ holds for any $A,B\in {\mathcal M}$. It is then sufficient to show $A\succcurlyeq _{\forall \forall \exists }^{{\mathcal W}} B$ $\iff$ $A\succcurlyeq _{\forall \forall \exists }^{{\mathcal W}} A\cup B$, which again easily follows by the definition. $\square$

#### Proposition 3

Consider any ${\mathcal W}\subseteq {\mathcal U}$ and $A, B\in {\mathcal M}$.

(i) $$A\succcurlyeq _{\forall \forall \exists }^{{\mathcal W}} B$$ $$\iff $$ for all $$w\in {\mathcal W}$$, $$\textit{Ut}_A(w) \ge \textit{Ut}_B(w)$$.
(ii) $A\equiv _{\forall \forall \exists }^{{\mathcal W}} B$ $\iff$ for all $w\in {\mathcal W}$, $\textit{Ut}_A(w) = \textit{Ut}_B(w)$.
(iii) $A\succcurlyeq _{\exists \forall \forall }^{{\mathcal W}} B$ if and only if there exists $\alpha \in A$ such that for all $w\in {\mathcal W}$, $f_w(\alpha ) \ge \textit{Ut}_B(w)$.

#### Proof

(i): [For all $w\in {\mathcal W}$, $\textit{Ut}_{A}(w) \ge \textit{Ut}_{B}(w)$] if and only if [for all $w\in {\mathcal W}$, $\max _{\beta \in B} f_w(\beta ) \le \max _{\alpha \in A} f_w(\alpha )$], which is if and only if for all $w\in {\mathcal W}$, for all $\beta \in B$, $f_w(\beta ) \le \max _{\alpha \in A} f_w(\alpha )$. This holds if and only if for all $w\in {\mathcal W}$ and $\beta \in B$ there exists $\alpha \in A$ such that $f_w(\alpha ) \ge f_w(\beta )$, which is if and only if $A\succcurlyeq _{\forall \forall \exists }^{{\mathcal W}} B$.

(ii): $A\equiv _{\forall \forall \exists }^{{\mathcal W}} B$ holds if and only if $A\succcurlyeq _{\forall \forall \exists }^{{\mathcal W}} B$ and $B\succcurlyeq _{\forall \forall \exists }^{{\mathcal W}} A$, which by (i) is if and only if for all $w\in {\mathcal W}$, $\textit{Ut}_B(w) \le \textit{Ut}_A(w)$ and $\textit{Ut}_B(w) \ge \textit{Ut}_A(w)$, which is if and only if $\textit{Ut}^{\mathcal W}_{B} =\textit{Ut}^{\mathcal W}_{A}$.

(iii): For $\alpha \in \Omega$, ${\{\alpha \}} \succcurlyeq _{\exists \forall \forall }^{{\mathcal W}} B$ $\iff$ ${\{\alpha \}} \succcurlyeq _{\forall \forall \exists }^{{\mathcal W}} B$. Then, $A\succcurlyeq _{\exists \forall \forall }^{{\mathcal W}} B$ if and only if there exists $\alpha \in A$ such that ${\{\alpha \}} \succcurlyeq _{\exists \forall \forall }^{{\mathcal W}} B$, which is if and only if there exists $\alpha \in A$ such that for all $w\in {\mathcal W}$, $f_w(\alpha ) \ge \textit{Ut}_B(w)$, using part (i). $\square$

### Results in Sect. 4

We give a simple fundamental property of the set $\mathrm{UD}_{\mathcal W}(A)$, which is used to prove e.g., Proposition 5 below.

#### Auxiliary Lemma 1

Consider any $$A\in {\mathcal M}$$.

(i) If $\alpha \in A\setminus \mathrm{UD}_{\mathcal W}(A)$ then there exists $\gamma \in \mathrm{UD}_{\mathcal W}(A)$ such that $\gamma \succ _{\mathcal W}\alpha$.
(ii) If $\alpha \in A$ then there exists $\gamma \in \mathrm{UD}_{\mathcal W}(A)$ such that $\gamma \succcurlyeq _{\mathcal W}\alpha$.

#### Proof

(i): Consider any $\alpha \in A\setminus \mathrm{UD}_{\mathcal W}(A)$. By the definition of $\mathrm{UD}_{\mathcal W}(A)$, for any $\beta \in A\setminus \mathrm{UD}_{\mathcal W}(A)$ there exists $\beta '\in A$ such that $\beta ' \succ _{\mathcal W}\beta$. Let $\alpha _1 = \alpha$. We construct a sequence $\alpha _1, \alpha _2, \ldots$, where for each $i = 1, 2,\ldots$, we have $\alpha _{i+1} \succ _{\mathcal W}\alpha _i$, where we stop the sequence when we reach an element $\alpha _i$ such that either (a) $\alpha _i$ has appeared earlier in the sequence, or (b) $\alpha _i \in \mathrm{UD}_{\mathcal W}(A)$. Because $A$ is finite, there must be a last element $\alpha _k$ in the sequence. Transitivity of $\succ _{\mathcal W}$ implies that if $1 \le i < k$ then $\alpha _k \succ _{\mathcal W}\alpha _i$, so, in particular, $\alpha _k \succ _{\mathcal W}\alpha$. If (a) $\alpha _k = \alpha _i$ for some $i < k$ then $\alpha _k \succ _{\mathcal W}\alpha _k$ which contradicts the fact that $\succ _{\mathcal W}$ is irreflexive. Thus, we have (b) $\alpha _k \in \mathrm{UD}_{\mathcal W}(A)$ and $\alpha _k \succ _{\mathcal W}\alpha$, showing part (i).

(ii): Consider any $\alpha \in A$. If $\alpha \in A\setminus \mathrm{UD}_{\mathcal W}(A)$ then, by part (i), there exists $\gamma \in \mathrm{UD}_{\mathcal W}(A)$ such that $\gamma \succcurlyeq _{\mathcal W}\alpha$. Otherwise, $\alpha \in \mathrm{UD}_{\mathcal W}(A)$, and, by reflexivity of $\succcurlyeq _{\mathcal W}$ we have $\alpha \succcurlyeq _{\mathcal W}\alpha$, so we can let $\gamma = \alpha$. $\square$

#### Proposition 5

Assume that ${\mathcal W}\subseteq {\mathcal U}$ and $A\in {\mathcal M}$. Then, $\mathrm{UD}_{\mathcal W}(A)$ is non-empty and the following hold.

(i) For $B\in {\mathcal M}$ and, for $\succcurlyeq$ being any of $\succcurlyeq _{\forall \forall \exists }^{{\mathcal W}}$, $\succcurlyeq _{\forall \exists \forall }^{{\mathcal W}}$ or $\succcurlyeq _{\exists \forall \forall }^{{\mathcal W}}$, we have $A\succcurlyeq B$ $\iff$ $\mathrm{UD}_{\mathcal W}(A) \succcurlyeq \mathrm{UD}_{\mathcal W}(B)$.
(ii) $\mathrm{UD}_{\mathcal W}(A) \equiv _{\forall \exists \forall }^{{\mathcal W}} A$ and $\mathrm{UD}_{\mathcal W}(A) \equiv _{\forall \forall \exists }^{{\mathcal W}} A$. Hence, if $A$ is non-empty then $\mathrm{UD}_{\mathcal W}(A)$ is also.

#### Proof

Auxiliary Lemma 1(ii) immediately implies that $\mathrm{UD}_{\mathcal W}(A)$ is non-empty. We next prove part (ii). Auxiliary Lemma 1(ii) implies that for all $\alpha \in A$ there exists $\gamma \in \mathrm{UD}_{\mathcal W}(A)$ such that $\gamma \succcurlyeq _{\mathcal W}\alpha$, and thus, $\mathrm{UD}_{\mathcal W}(A) \succcurlyeq _{\forall \exists \forall }^{{\mathcal W}} A$. Since $\mathrm{UD}_{\mathcal W}(A) \subseteq A$, by Proposition 4, we have $A\succcurlyeq _{\forall \exists \forall }^{{\mathcal W}} \mathrm{UD}_{\mathcal W}(A)$, and hence, $\mathrm{UD}_{\mathcal W}(A) \equiv _{\forall \exists \forall }^{{\mathcal W}} A$. Proposition 1 implies that $\succcurlyeq _{\forall \exists \forall }^{{\mathcal W}}$ $\subseteq$ $\succcurlyeq _{\forall \forall \exists }^{{\mathcal W}}$ and hence $\equiv _{\forall \exists \forall }^{{\mathcal W}}$ $\subseteq$ $\equiv _{\forall \forall \exists }^{{\mathcal W}}$. Thus we also have $\mathrm{UD}_{\mathcal W}(A) \equiv _{\forall \forall \exists }^{{\mathcal W}} A$.

Part (ii) implies part (i) when $\succcurlyeq$ is either $\succcurlyeq _{\forall \forall \exists }^{{\mathcal W}}$ or $\succcurlyeq _{\forall \exists \forall }^{{\mathcal W}}$. This is because, if $\equiv$ is the corresponding equivalence relation, then $\mathrm{UD}_{\mathcal W}(A) \equiv A$ and $\mathrm{UD}_{\mathcal W}(B) \equiv B$. Then, $A\succcurlyeq B$ implies $\mathrm{UD}_{\mathcal W}(A) \equiv A\succcurlyeq B\equiv \mathrm{UD}_{\mathcal W}(B)$, and thus, $\mathrm{UD}_{\mathcal W}(A) \succcurlyeq \mathrm{UD}_{\mathcal W}(B)$ by transitivity of $\succcurlyeq$. Similarly, $\mathrm{UD}_{\mathcal W}(A) \succcurlyeq \mathrm{UD}_{\mathcal W}(B)$ implies $A\succcurlyeq \mathrm{UD}_{\mathcal W}(A) \succcurlyeq \mathrm{UD}_{\mathcal W}(B) \succcurlyeq B$ and thus, $A\succcurlyeq B$.

Finally, we will prove that $A\succcurlyeq _{\exists \forall \forall }^{{\mathcal W}} B$ $\iff$ $\mathrm{UD}_{\mathcal W}(A) \succcurlyeq _{\exists \forall \forall }^{{\mathcal W}} \mathrm{UD}_{\mathcal W}(B)$. We have, by part (ii), $A\succcurlyeq _{\forall \exists \forall }^{{\mathcal W}} \mathrm{UD}_{\mathcal W}(A)$ and $B\succcurlyeq _{\forall \exists \forall }^{{\mathcal W}} \mathrm{UD}_{\mathcal W}(B)$. First suppose, $A\succcurlyeq _{\exists \forall \forall }^{{\mathcal W}} B$. We have $\mathrm{UD}_{\mathcal W}(A) \succcurlyeq _{\forall \exists \forall }^{{\mathcal W}} A\succcurlyeq _{\exists \forall \forall }^{{\mathcal W}} B\succcurlyeq _{\forall \exists \forall }^{{\mathcal W}} \mathrm{UD}_{\mathcal W}(B)$. Applying parts (a) and (b) of Proposition 1 implies $\mathrm{UD}_{\mathcal W}(A) \succcurlyeq _{\exists \forall \forall }^{{\mathcal W}} \mathrm{UD}_{\mathcal W}(B)$.

Now, assume that $\mathrm{UD}_{\mathcal W}(A) \succcurlyeq _{\exists \forall \forall }^{{\mathcal W}} \mathrm{UD}_{\mathcal W}(B)$. We have $A\succcurlyeq _{\forall \exists \forall }^{{\mathcal W}} \mathrm{UD}_{\mathcal W}(A) \succcurlyeq _{\exists \forall \forall }^{{\mathcal W}} \mathrm{UD}_{\mathcal W}(B)$ $\succcurlyeq _{\forall \exists \forall }^{{\mathcal W}} B$. Applying again parts (a) and (b) of Proposition 1 we obtain $A\succcurlyeq _{\exists \forall \forall }^{{\mathcal W}} B$. $\square$

*Equivalence relation* $\equiv _{\mathcal W}$ *on* ${\mathcal M}$: We extend the notation $\equiv _{\mathcal W}$ as an equivalence relation on ${\mathcal M}$ as follows: $A\equiv _{\mathcal W}B$ $\iff$ for all $\alpha \in A$ there exists $\beta \in B$ such that $\alpha \equiv _{\mathcal W}\beta$, and vice versa, i.e., for all $\beta \in B$ there exists $\alpha \in A$ such that $\alpha \equiv _{\mathcal W}\beta$. We then have, $A\equiv _{\forall \exists \forall }^{{\mathcal W}} B$ if and only if for every undominated element of $A$ there exists an equivalent element in $B$, and vice versa.

#### Lemma 1

Let ${\mathcal W}\subseteq {\mathcal U}$ and let $A\in {\mathcal M}$. For $B\subseteq A$, $B\equiv _{\forall \forall \exists }^{{\mathcal W}} A$ if and only if $\bigcup _{\beta \in B} \mathrm{Opt}_{\mathcal W}^A(\beta ) = {\mathcal W}$. In particular, $\bigcup _{\alpha \in A} \mathrm{Opt}_{\mathcal W}^A(\alpha ) = {\mathcal W}$.

#### Proof

We first prove that $\bigcup _{\alpha \in A} \mathrm{Opt}_{\mathcal W}^A(\alpha ) = {\mathcal W}$. By definition we have $\bigcup _{\alpha \in A} \mathrm{Opt}_{\mathcal W}^A(\alpha ) \subseteq {\mathcal W}$. Now, consider any $w\in {\mathcal W}$. $\mathrm{O}_w(A)$ is clearly non-empty (since $A$ is finite and $\succcurlyeq _w$ is a total pre-order), so let $\alpha$ be some element of it. Then $\mathrm{Opt}_{\mathcal W}^A(\alpha ) \ni w$, so $\bigcup _{\alpha \in A} \mathrm{Opt}_{\mathcal W}^A(\alpha ) \supseteq {\mathcal W}$.

Since $B\subseteq A$, we have $B\equiv _{\forall \forall \exists }^{{\mathcal W}} A$ $\iff$ $B\succcurlyeq _{\forall \forall \exists }^{{\mathcal W}} A$ $\iff$ for all $w\in {\mathcal W}$ and for all $\alpha \in A$ there exists $\beta \in B$ such that $\beta \succcurlyeq _w\alpha$. This holds if and only if for all $w\in {\mathcal W}$ there exists $\beta \in B$ such that for all $\alpha \in A$, $\beta \succcurlyeq _w\alpha$, i.e., for all $w\in {\mathcal W}$ there exists $\beta \in B$ such that $\mathrm{Opt}_{\mathcal W}^A(\beta ) \ni w$, which is equivalent to $\bigcup _{\beta \in B} \mathrm{Opt}_{\mathcal W}^A(\beta ) \supseteq {\mathcal W}$, i.e., $\bigcup _{\beta \in B} \mathrm{Opt}_{\mathcal W}^A(\beta ) = {\mathcal W}$. $\square$

In the following lemma we give some basic properties of the operator $\mathrm{PO}_{\mathcal W}$ which can be easily proved.

#### Auxiliary Lemma 2

Assume that ${\mathcal W}\subseteq {\mathcal U}$ and $A\in {\mathcal M}$. Then, the following all hold.

(i) $\mathrm{PO}_{\mathcal W}(A)$ is non-empty, and for all $w\in {\mathcal W}$, $\mathrm{O}_w(A)$ is non-empty.
(ii) For all $w\in {\mathcal W}$ and for all $\alpha \in A\setminus \mathrm{O}_w(A)$ there exists $\gamma \in \mathrm{O}_w(A)$ such that $\gamma \succ _w\alpha$, and thus, there exists $\gamma \in \mathrm{PO}_{\mathcal W}(A)$ such that $\gamma \succ _w\alpha$.
(iii) For $B\subseteq \Omega$ we have $A\succcurlyeq _{\forall \forall \exists }^{{\mathcal W}} B$ $\iff$ $\mathrm{PO}_{\mathcal W}(A) \succcurlyeq _{\forall \forall \exists }^{{\mathcal W}} B$; and $B\succcurlyeq _{\forall \forall \exists }^{{\mathcal W}} A$ $\iff$ $B\succcurlyeq _{\forall \forall \exists }^{{\mathcal W}} \mathrm{PO}_{\mathcal W}(A)$. Thus, if $B$ is also finite then $A\succcurlyeq _{\forall \forall \exists }^{{\mathcal W}} B$ $\iff$ $\mathrm{PO}_{\mathcal W}(A) \succcurlyeq _{\forall \forall \exists }^{{\mathcal W}} \mathrm{PO}_{\mathcal W}(B)$.
(iv) $$\mathrm{PO}_{\mathcal W}(A) \equiv _{\forall \forall \exists }^{{\mathcal W}} A$$.

#### Proposition 6

Consider any ${\mathcal W}\subseteq {\mathcal U}$ and a set $A\in {\mathcal M}$.

(i) For $B\subseteq A$, if $B\equiv _{\forall \forall \exists }^{{\mathcal W}} A$ then for all $\alpha \in \mathrm{PSO}_{\mathcal W}(A)$ there exists $\beta \in B$ with $\beta \equiv _{\mathcal W}\alpha$. If, in addition, $A$ is $\equiv _{\mathcal W}$-free then $B\supseteq \mathrm{PSO}_{\mathcal W}(A)$.
(ii) If $\alpha \in A\setminus \mathrm{PSO}_{\mathcal W}(A)$ then $\bigcup _{\beta \in A\setminus {\{\alpha \}}} \mathrm{Opt}_{\mathcal W}^A(\beta ) = {\mathcal W}$ and so $A\setminus {\{\alpha \}} \equiv _{\forall \forall \exists }^{{\mathcal W}} A$.
(iii) For $\equiv _{\mathcal W}$-free $A$, $\mathrm{PSO}_{\mathcal W}(A)$ is the set of all $\alpha \in A$ such that $A\setminus {\{\alpha \}} \not \succcurlyeq _{\forall \forall \exists }^{{\mathcal W}} {\{\alpha \}}$.

#### Proof

(i) Assume that $B\equiv _{\forall \forall \exists }^{{\mathcal W}} A$. By Lemma 1, $\bigcup _{\beta \in B} \mathrm{Opt}_{\mathcal W}^A(\beta ) = {\mathcal W}$. Consider any $\alpha \in \mathrm{PSO}_{\mathcal W}(A)$. Then there exists $w\in {\mathcal W}$ such that $\mathrm{SO}_w^{\mathcal W}(A) \ni \alpha$. Also, there exists $\beta \in B$ such that $\mathrm{Opt}_{\mathcal W}^A(\beta ) \ni w$, and so, $\mathrm{O}_w(A) \ni \beta$. The definition of $\mathrm{SO}_w^{\mathcal W}(A)$ implies that $\beta \equiv _{\mathcal W}\alpha$. In particular, if $A$ is $\equiv _{\mathcal W}$-free then for all $\alpha \in \mathrm{PSO}_{\mathcal W}(A)$, $\alpha \in B$, so $B\supseteq \mathrm{PSO}_{\mathcal W}(A)$.

(ii): Suppose that $\alpha \in A\setminus \mathrm{PSO}_{\mathcal W}(A)$ and consider any $w\in {\mathcal W}$. Then $\mathrm{O}_w(A) \not ={\{\alpha \}}$ so there exists $\beta \in \mathrm{O}_w(A)\setminus {\{\alpha \}}$, and thus, $\mathrm{Opt}_{\mathcal W}^A(\beta ) \ni w$. This shows that $\bigcup _{\beta \in A\setminus {\{\alpha \}}} \mathrm{Opt}_{\mathcal W}^A(\beta ) = {\mathcal W}$, and thus, by Lemma 1, $A\setminus {\{\alpha \}} \equiv _{\forall \forall \exists }^{{\mathcal W}} A$.

(iii): Consider arbitrary $\alpha \in A$. We will prove part (iii) by showing that $\alpha \notin \mathrm{PSO}_{\mathcal W}(A)$ $\iff$ $A\setminus {\{\alpha \}} \succcurlyeq _{\forall \forall \exists }^{{\mathcal W}} {\{\alpha \}}$.

First suppose that $\alpha \notin \mathrm{PSO}_{\mathcal W}(A)$; then by Proposition 6(ii), $A\setminus {\{\alpha \}} \equiv _{\forall \forall \exists }^{{\mathcal W}} A$, and thus, $A\setminus {\{\alpha \}} \succcurlyeq _{\forall \forall \exists }^{{\mathcal W}} {\{\alpha \}}$.

Now assume that $A\setminus {\{\alpha \}} \succcurlyeq _{\forall \forall \exists }^{{\mathcal W}} {\{\alpha \}}$, which implies, using Proposition 2, that $A\setminus {\{\alpha \}} \equiv _{\forall \forall \exists }^{{\mathcal W}} A$. Since $A$ is $\equiv _{\mathcal W}$-free, we have by part (i) that $A\setminus {\{\alpha \}} \supseteq \mathrm{PSO}_{\mathcal W}(A)$, showing that $\alpha \notin \mathrm{PSO}_{\mathcal W}(A)$. $\square$

#### Proposition 7

Assume that ${\mathcal W}\subseteq {\mathcal U}$ and $A\in {\mathcal M}$. Then the following hold:

(i) $$\mathrm{PSO}_{\mathcal W}(A) \subseteq \mathrm{MPO}_{\mathcal W}(A) \cap \mathrm{UD}_{\mathcal W}(A) \subseteq \mathrm{MPO}_{\mathcal W}(A) \subseteq \mathrm{PO}_{\mathcal W}(A) \subseteq A$$.
(ii) $\mathrm{OP}_{\mathcal W}(A)\equiv _{\forall \forall \exists }^{{\mathcal W}} A$ if $\mathrm{OP}_{\mathcal W}(A)$ is any of the following: $\mathrm{UD}_{\mathcal W}(A)$, $\mathrm{PO}_{\mathcal W}(A)$, $\mathrm{MPO}_{\mathcal W}(A)$, $\mathrm{PO}_{\mathcal W}(A) \cap \mathrm{UD}_{\mathcal W}(A)$, or $\mathrm{MPO}_{\mathcal W}(A) \cap \mathrm{UD}_{\mathcal W}(A)$. Thus, $A\succcurlyeq _{\forall \forall \exists }^{{\mathcal W}} B$ $\iff$ $\mathrm{OP}_{\mathcal W}(A) \succcurlyeq _{\forall \forall \exists }^{{\mathcal W}} \mathrm{OP}_{\mathcal W}(B)$.

#### Proof

(i) follows easily from the definitions. Regarding (ii): we first show that $\mathrm{MPO}_{\mathcal W}(A) \cap \mathrm{UD}_{\mathcal W}(A) \equiv _{\forall \forall \exists }^{{\mathcal W}} A$. Since $\mathrm{MPO}_{\mathcal W}(A) \cap \mathrm{UD}_{\mathcal W}(A) \subseteq A$ we have $A\succcurlyeq _{\forall \forall \exists }^{{\mathcal W}} \mathrm{MPO}_{\mathcal W}(A) \cap \mathrm{UD}_{\mathcal W}(A)$. We need to show the converse, that $\mathrm{MPO}_{\mathcal W}(A) \cap \mathrm{UD}_{\mathcal W}(A) \succcurlyeq _{\forall \forall \exists }^{{\mathcal W}} A$. Consider any $w\in {\mathcal W}$ and $\alpha \in A$; it is sufficient to show that there exists $\beta \in \mathrm{MPO}_{\mathcal W}(A) \cap \mathrm{UD}_{\mathcal W}(A)$ with $\beta \succcurlyeq _w\alpha$. Finiteness of $A$ implies that there exists $\gamma \in A$ such that (a) $\gamma \in \mathrm{O}_w(A)$, i.e., $w\in \mathrm{Opt}_{\mathcal W}^A(\gamma )$, and (b) there does not exist $\delta \in A$ with $\mathrm{Opt}_{\mathcal W}^A(\delta ) \supsetneqq \mathrm{Opt}_{\mathcal W}^A(\gamma )$, and thus, $\gamma \in \mathrm{MPO}_{\mathcal W}(A)$. There exists $\beta \in \mathrm{UD}_{\mathcal W}(A)$ with $\beta \succcurlyeq _{\mathcal W}\gamma$, which implies that $\beta \in \mathrm{MPO}_{\mathcal W}(A) \cap \mathrm{UD}_{\mathcal W}(A)$ and $\beta \succcurlyeq _w\alpha$, as required.

Now, if $\mathrm{MPO}_{\mathcal W}(A) \cap \mathrm{UD}_{\mathcal W}(A) \subseteq B\subseteq A$ then $A\succcurlyeq _{\forall \forall \exists }^{{\mathcal W}} B\succcurlyeq _{\forall \forall \exists }^{{\mathcal W}} \mathrm{MPO}_{\mathcal W}(A) \cap \mathrm{UD}_{\mathcal W}(A) \succcurlyeq _{\forall \forall \exists }^{{\mathcal W}} A$ and so $A\equiv _{\forall \forall \exists }^{{\mathcal W}} B$. Using part (i), we thus have $\mathrm{OP}_{\mathcal W}(A)\equiv _{\forall \forall \exists }^{{\mathcal W}} A$ if $\mathrm{OP}_{\mathcal W}(A)$ is any of the following: $\mathrm{UD}_{\mathcal W}(A)$, $\mathrm{PO}_{\mathcal W}(A)$, $\mathrm{MPO}_{\mathcal W}(A)$, $\mathrm{PO}_{\mathcal W}(A) \cap \mathrm{UD}_{\mathcal W}(A)$, or $\mathrm{MPO}_{\mathcal W}(A) \cap \mathrm{UD}_{\mathcal W}(A)$. $\square$

### Results in Sect. 5

#### Proposition 8

Consider an arbitrary set of scenarios ${\mathcal W}$ ($\subseteq {\mathcal U}$) and suppose $A,B\in {\mathcal M}$ with $B\subseteq A$.

(i) $$\mathrm{PSO}_{\mathcal W}(A) \cap B\subseteq \mathrm{PSO}_{\mathcal W}(B)$$; and
(ii) if $\mathrm{PSO}_{\mathcal W}(A) \equiv _{\forall \forall \exists }^{{\mathcal W}} A$, and $\mathrm{PSO}_{\mathcal W}(A) \subseteq B$ then $\mathrm{PSO}_{\mathcal W}(B) = \mathrm{PSO}_{\mathcal W}(A)$;
(iii) if $\mathrm{PSO}_{\mathcal W}(B) \equiv _{\forall \forall \exists }^{{\mathcal W}} B$ for all $B\subseteq A$ then $\mathrm{PSO}_{\mathcal W}$ satisfies path independence on $2^A$, i.e., for any subsets $B$ and $C$ of $A$, $\mathrm{PSO}_{{\mathcal W}}(B\cup C) = \mathrm{PSO}_{{\mathcal W}}(\mathrm{PSO}_{{\mathcal W}}(B) \cup C)$.

#### Proof

(i) Consider any $\alpha \in \mathrm{PSO}_{\mathcal W}(A) \cap B$. Then there exists $w\in {\mathcal W}$ such that $\mathrm{SO}_w^{\mathcal W}(A)$ contains $\alpha$. If $\beta \in B$ is such that $\alpha \not \equiv _{\mathcal W}\beta$ then $\beta \in A$ and so, since $\alpha$ is strictly optimal within $A$ in scenario $w$, we have $\alpha \succ _w\beta$, which shows that $\alpha \in \mathrm{SO}_w^{\mathcal W}(B)$ and hence, $\alpha \in \mathrm{PSO}_{\mathcal W}(B)$.

(ii) Now assume that $\mathrm{PSO}_{\mathcal W}(A) \equiv _{\forall \forall \exists }^{{\mathcal W}} A$ and $\mathrm{PSO}_{\mathcal W}(A) \subseteq B$ ($\subseteq A$). Part (i) implies $\mathrm{PSO}_{\mathcal W}(A) \subseteq \mathrm{PSO}_{\mathcal W}(B)$. Consider now any element $\beta \in \mathrm{PSO}_{\mathcal W}(B)$; it is sufficient to show that $\beta \in \mathrm{PSO}_{\mathcal W}(A)$. So, to prove a contradiction, let us assume that $\beta \notin \mathrm{PSO}_{\mathcal W}(A)$. Let $C= {\{\gamma \in \mathrm{PSO}_{\mathcal W}(B) \, : \,\gamma \equiv _{\mathcal W}\beta \}}$. We have $C\cap \mathrm{PSO}_{\mathcal W}(A) = \emptyset$, so $\mathrm{PSO}_{\mathcal W}(A) \subseteq \mathrm{PSO}_{\mathcal W}(B)\setminus C\subseteq \mathrm{PSO}_{\mathcal W}(B)\subseteq A$. Since $\mathrm{PSO}_{\mathcal W}(A) \equiv _{\forall \forall \exists }^{{\mathcal W}} A$ we have $\mathrm{PSO}_{\mathcal W}(B)\setminus C\equiv _{\forall \forall \exists }^{{\mathcal W}} \mathrm{PSO}_{\mathcal W}(B)$. Since $\beta \in \mathrm{PSO}_{\mathcal W}(B)$ there exists $w\in {\mathcal W}$ that makes $\beta$ strictly optimal in $B$, i.e., such that $\beta \in \mathrm{SO}_w^{\mathcal W}(B)$. If $\gamma \in B{\setminus }C$ then $f^\gamma (w) < f^\beta (w)$. In particular, this holds for $\gamma \in \mathrm{PSO}_{\mathcal W}(B)\setminus C$, and thus, $\mathrm{PSO}_{\mathcal W}(B)\setminus C\not \equiv _{\forall \forall \exists }^{{\mathcal W}} \mathrm{PSO}_{\mathcal W}(B)$, which is the required contradiction.

(iii) It is well known (see e.g., Proposition 1 of [21]) that path independence is equivalent to the pair of properties expressed by (i) and (ii), so (iii) follows from (i) and (ii). $\square$

#### Lemma 2

Suppose that subset ${\mathcal T}$ of ${\mathcal W}$ only contains $w$ that are total over $A$ given ${\mathcal W}$. Then for all $B\subseteq A$, $\mathrm{PSO}_{{\mathcal T}}(B) = \mathrm{PO}_{{\mathcal T}}(B)$.

#### Proof

For any $w$ that is total over $A$ given ${\mathcal W}$, we have $\mathrm{O}_w(B) = \mathrm{SO}_w^{\mathcal W}(B)$, which implies the result, by definitions of $\mathrm{PSO}_{{\mathcal T}}(B)$ and $\mathrm{PO}_{{\mathcal T}}(B)$. $\square$

#### Lemma 4

Suppose that ${\mathcal W}$ is $A$-Extendable. Let ${\mathcal V}= {\mathcal W}_A^{\not =}$. Then, for any $B,C\subseteq A$, we have

(i) $$B\succcurlyeq _{\forall \forall \exists }^{{\mathcal V}} C$$ $$\iff $$ $$B\succcurlyeq _{\forall \forall \exists }^{{\mathcal W}} C$$.
(ii) $$\mathrm{PSO}_{{\mathcal V}}(B) = \mathrm{PSO}_{{\mathcal W}}(B)$$.

#### Proof

(i) $\Rightarrow$: Assume $B\succcurlyeq _{\forall \forall \exists }^{{\mathcal V}} C$, and consider arbitrary $\gamma \in C$ and $w\in {\mathcal W}$. Since ${\mathcal W}$ is $A$-Extendable, there exists $w'$ that is total over $A$ given ${\mathcal W}$ (so $w'\in {\mathcal V}$) that extends $w$ over $A$. Since we have $B\succcurlyeq _{\forall \forall \exists }^{{\mathcal V}} C$, there exists $\beta \in B$ such that $\beta \succcurlyeq _{w'} \gamma$. But, $\beta \succcurlyeq _{w'} \gamma$ implies that $\beta \succcurlyeq _{w} \gamma$, showing that $B\succcurlyeq _{\forall \forall \exists }^{{\mathcal W}} C$.

The $\Leftarrow$ part of (i) is immediate from monotonicity with respect to ${\mathcal W}$ (see Proposition 4).

(ii): First assume that $\beta \in \mathrm{PSO}_{{\mathcal V}}(B)$, so there exists $w\in {\mathcal V}$ such that $\beta \in \mathrm{SO}_w^{{\mathcal V}}(B)$. But since, by Lemma 3, $\beta \equiv _{{\mathcal V}} \gamma$ $\iff$ $\beta \equiv _{{\mathcal W}} \gamma$ for $\beta ,\gamma \in A$, we have $\mathrm{SO}_w^{{\mathcal V}}(B) = \mathrm{SO}_w^{{\mathcal W}}(B)$, and thus, $\beta \in \mathrm{SO}_w^{{\mathcal W}}(B)$, so $\beta \in \mathrm{PSO}_{{\mathcal W}}(B)$.

Conversely, assume that $\beta \in \mathrm{PSO}_{{\mathcal W}}(B)$, so there exists $w\in {\mathcal W}$ such that $\beta \in \mathrm{SO}_w^{{\mathcal W}}(B)$. Since ${\mathcal W}$ is $A$-Extendable, there exists $w' \in {\mathcal V}$ that extends $w$ over $A$. Consider any $\gamma \in B$ such that $\gamma \not \equiv _{{\mathcal V}} \beta$, and so $\gamma \not \equiv _{{\mathcal W}} \beta$. Since $\beta \in \mathrm{SO}_w^{{\mathcal W}}(B)$, $\beta \succ _w\gamma$, and thus, $\beta \succ _{w'} \gamma$, since $w'$ extends $w$. This shows that $\beta \in \mathrm{SO}_{w'}^{{\mathcal V}}(B)$, and thus, $\beta \in \mathrm{PSO}_{{\mathcal V}}(B)$, as required. $\square$

### Results in Sect. 6

We define for ${\mathcal W}\subseteq {\mathcal U}$ and $\alpha ,\beta \in \Omega$,

- $${\mathcal W}_{\alpha \ge \beta } = {\{w\in {\mathcal W}\, : \,f^\alpha (w) \ge f^\beta (w) \}} $$;
- $${\mathcal W}_{\alpha>\beta } = {\{w\in {\mathcal W}\, : \,f^\alpha (w) > f^\beta (w) \}} $$;
- $${\mathcal W}_{\alpha =\beta } = {\{w\in {\mathcal W}\, : \,f^\alpha (w) = f^\beta (w) \}} $$.

For instance, ${\mathcal W}_{\alpha \ge \beta }$ is the set of scenarios $w\in {\mathcal W}$ in which $\alpha$ is at least as good as $\beta$.

#### Auxiliary Lemma 3

Assume that $f^\alpha$ and $f^\beta$ are real-valued continuous functions on the metric space ${\mathcal W}$. Then ${\mathcal W}_{\alpha >\beta }$ and ${\mathcal W}_{\alpha \not =\beta }$ are open subsets of ${\mathcal W}$.

#### Proof

Consider any $\alpha ,\beta \in \Omega$. The function $G: {\mathcal W}\rightarrow {I\!R}$, given by $G(w) = f_w(\alpha ) - f_w(\beta )$, is continuous. Then ${\mathcal W}_{\alpha >\beta } =G^{-1}((0, \infty ))$, and ${\mathcal W}_{\alpha \not =\beta } =G^{-1}({I\!R}\setminus {\{0\}}))$. Since $(0, \infty )$ and ${I\!R}{\setminus }{\{0\}}$ are open subsets of the reals, $G^{-1}((0, \infty ))$ and $G^{-1}({I\!R}{\setminus }{\{0\}}))$ are open, so ${\mathcal W}_{\alpha >\beta }$ and ${\mathcal W}_{\alpha \not =\beta }$ are open. $\square$

Recall that $\mathrm{Opt}_{\mathcal W}^A(\alpha )$ is the set of elements in ${\mathcal W}$ that make $\alpha$ optimal in $A$. Similarly, we define $\mathrm{SOpt}_{\mathcal W}^A(\alpha )$ to be the set of elements in ${\mathcal W}$ that make $\alpha$ strictly optimal in $A$.

*Definition of* $$\mathrm{SOpt}_{\mathcal W}^A(\alpha )$$:

We define, for $\alpha \in A$, $\mathrm{SOpt}_{\mathcal W}^A(\alpha )$ to consist of all scenarios $w\in {\mathcal W}$ in which $\alpha$ is strictly optimal, i.e., $\alpha \in \mathrm{SO}_w^{\mathcal W}(A)$. Thus, $\alpha \in \mathrm{PSO}_{\mathcal W}(A)$ $\iff$ $\mathrm{SOpt}_{\mathcal W}^A(\alpha ) \not =\emptyset$. Also, $w\in \mathrm{SOpt}_{\mathcal W}^A(\alpha )$ if and only if $w\in {\mathcal W}$ and $f^\alpha (w) > f^\beta (w)$ for all $\beta \in A$ such that $\alpha \not \equiv _{\mathcal W}\beta$.

The next two results give some basic properties.

#### Auxiliary Lemma 4

Let ${\mathcal W}\subseteq {\mathcal U}$ and let $A\in {\mathcal M}$, and let $\alpha \in A$.

(i) $$\mathrm{Opt}_{\mathcal W}^A(\alpha ) = \bigcap _{\beta \in A} {\mathcal W}_{\alpha \ge \beta }$$ and $$\mathrm{SOpt}_{\mathcal W}^A(\alpha ) = \bigcap _{\beta \in A: \alpha \not \equiv _{\mathcal W}\beta } {\mathcal W}_{\alpha >\beta }$$.
(ii) $${\mathcal W}_A^{\not =}\subseteq \bigcup _{\gamma \in A} \mathrm{SOpt}_{\mathcal W}^A(\gamma )$$.

#### Proof

(i): $w\in \mathrm{Opt}_{\mathcal W}^A(\alpha )$ if and only if for all $\beta \in A$, $f^\alpha (w) \ge f^\beta (w)$, which is if and only if for all $\beta \in A$, $w\in {\mathcal W}_{\alpha \ge \beta }$, i.e., $w\in \bigcap _{\beta \in A} {\mathcal W}_{\alpha \ge \beta }$.

Similarly, $w\in \mathrm{SOpt}_{\mathcal W}^A(\alpha )$ if and only if $w\in {\mathcal W}$ and $f^\alpha (w) > f^\beta (w)$ for all $\beta \in A$ such that $\alpha \not \equiv _{\mathcal W}\beta$, which is if and only if $w\in \bigcap _{\beta \in A: \alpha \not \equiv _{\mathcal W}\beta } {\mathcal W}_{\alpha >\beta }$.

(ii): Consider any $w\in {\mathcal W}_A^{\not =}$, so that $f^\alpha (w) \not = f^\beta (w)$ holds for all $\alpha , \beta \in A$ with $\alpha \not \equiv _{\mathcal W}\beta$. Let $\alpha$ be an element of $A$ with maximal value of $f^\alpha (w)$. Then, for all $\beta \in A$ such that $\alpha \not \equiv _{\mathcal W}\beta$, we have $f^\alpha (w) >f^\beta (w)$; thus, $w\in \mathrm{SOpt}_{\mathcal W}^A(\alpha )$, proving that $w\in \bigcup _{\gamma \in A} \mathrm{SOpt}_{\mathcal W}^A(\gamma )$. $\square$

#### Auxiliary Lemma 5

Let $A\in {\mathcal M}$ and assume, for each $\alpha \in A$, that the function $f^\alpha$ is a real-valued continuous function on the metric space ${\mathcal W}$. Then $\mathrm{SOpt}_{\mathcal W}^A(\alpha )$ is an open subset of ${\mathcal W}$ and $\mathrm{Opt}_{\mathcal W}^A(\alpha )$ is a closed subset of ${\mathcal W}$.

#### Proof

By Auxiliary Lemmas 4 and 3, $\mathrm{SOpt}_{\mathcal W}^A(\alpha )$ is a finite intersection of open subsets of ${\mathcal W}$, and thus is open, and $\mathrm{Opt}_{\mathcal W}^A(\alpha )$ is an intersection of closed subsets of ${\mathcal W}$, and thus is closed. $\square$

The small result below regarding closure of topological spaces will be useful in proving the equivalence expressed in Lemma 5.

#### Auxiliary Lemma 6

Let $(X,\tau )$ be a topological space and for $i=1,\ldots , m$, let $T_i$ be subsets of *X*. If each $T_i$ is an open subset of *X* (i.e., $T_i \in \tau$) then $\mathrm{Cl}(T_1 \cap \cdots \cap T_m) = X$ if and only if for all $i=1,\ldots ,m$, $\mathrm{Cl}(T_i) = X$.

#### Proof

For any $i=1,\ldots ,m$, $\mathrm{Cl}(T_i)$ is a closed set containing $T_1 \cap \cdots \cap T_m$, so $\mathrm{Cl}(T_1 \cap \cdots \cap T_m) \subseteq \mathrm{Cl}(T_i) \subseteq X$. Hence, if $\mathrm{Cl}(T_1 \cap \cdots \cap T_m) = X$ then for all $i=1,\ldots ,m$, $\mathrm{Cl}(T_i) = X$.

For the converse, we need to show that for all $i=1,\ldots ,m$, $\mathrm{Cl}(T_i) = X$ implies that $\mathrm{Cl}(T_1 \cap \cdots \cap T_m) = X$. It is sufficient to prove this for the case when $m=2$, since we can then apply this iteratively, to show that first $\mathrm{Cl}(T_1 \cap T_2) = X$ and then $\mathrm{Cl}((T_1 \cap T_2) \cap T_3) = X$, using the fact that $T_1 \cap T_2$ is an open set, and so on, to prove that $\mathrm{Cl}((T_1 \cap \cdots \cap T_{m-1}) \cap T_m) = X$.

So, assume that $\mathrm{Cl}(T_1) = \mathrm{Cl}(T_2) = X$; we need to show that $\mathrm{Cl}(T_1 \cap T_2) = X$. Let $S = X \setminus \mathrm{Cl}(T_1 \cap T_2)$, which is an open set. We have $(S \cap T_1) \cap T_2 = \emptyset$, and $S \cap T_1$ is an open set, so $\mathrm{Cl}(T_2) = X$ implies that $S \cap T_1 = \emptyset$ (else $X\setminus (S\cap T_1)$ is a closed set between $T_2$ and *X*). Similarly, $\mathrm{Cl}(T_1) = X$ then implies that $S = \emptyset$, so $\mathrm{Cl}(T_1 \cap T_2) = X$. $\square$

#### Proposition 9

Assume that each $\alpha$ in finite set $A$ is associated with a vector $\hat{\alpha }\in {I\!R}^{p}$. Let ${\mathcal W}$ be a convex subset of ${I\!R}^{p}$, and define, for each $\alpha \in A$, the function $f^\alpha$ by $f^\alpha (w) = \hat{\alpha }\cdot w$, representing the utility of alternative $\alpha$ in scenario $w$. Then the set of functions ${\{f^\alpha \, : \,\alpha \in A\}}$ satisfies the Identity Property.

#### Proof

Consider any $\alpha ,\beta \in A$ and any non-empty open subset *T* of ${\mathcal W}$, and suppose that $f^\alpha (w) = f^\beta (w)$ for all $w\in T$. To prove the Identity property it is sufficient to show that $f^\alpha (w) = f^\beta (w)$ for all $w\in {\mathcal W}$. Consider an arbitrary element $w$ of ${\mathcal W}$, and any element $u$ of *T*. Because *T* is an open subset of ${\mathcal W}$, it contains some open ball around $u$, and so there exists some $\epsilon > 0$ such that $\epsilon w+ (1-\epsilon ) u$ is in *T*. Let $v= \epsilon w+ (1-\epsilon ) u$. Since $u\in T$ we have $\hat{\alpha }\cdot u= \hat{\beta }\cdot u$, and hence $(\hat{\alpha }- \hat{\beta }) \cdot u= 0$. Since $v\in T$ we have $(\hat{\alpha }- \hat{\beta }) \cdot v= 0$. Thus, $0 = (\hat{\alpha }- \hat{\beta }) \cdot v= \epsilon (\hat{\alpha }- \hat{\beta }) \cdot w+ (1-\epsilon ) (\hat{\alpha }- \hat{\beta }) \cdot u= \epsilon (\hat{\alpha }- \hat{\beta }) \cdot w$, which implies that $(\hat{\alpha }- \hat{\beta }) \cdot w= 0$, and therefore, $f^\alpha (w) = f^\beta (w)$. $\square$

#### Lemma 5

Let $A\in {\mathcal M}$ and assume, for each $\alpha \in A$, that the function $f^\alpha$ is a real-valued continuous function on the metric space ${\mathcal W}$. The three conditions below are equivalent, i.e., if one holds then the other two also hold.

- The set of functions ${\{f^\alpha \, : \,\alpha \in A\}}$ on ${\mathcal W}$ satisfies the Identity property.
- For all $\alpha , \beta \in A$ with $\alpha \not \equiv _{\mathcal W}\beta$, we have $\mathrm{Cl}({\mathcal W}_{\alpha \not =\beta }) = {\mathcal W}$.
- $$\mathrm{Cl}({\mathcal W}_A^{\not =}) = {\mathcal W}$$.

#### Proof

Note that, for $\alpha ,\beta \in A$ we have $\alpha \equiv _{\mathcal W}\beta$ if and only if $f^\alpha$ and $f^\beta$ agree on ${\mathcal W}$. Consider an arbitrary pair of elements $\alpha ,\beta \in A$ such that $\alpha \not \equiv _{\mathcal W}\beta$. To prove the equivalence of (a) and (b) it is sufficient to show that (I) $f^\alpha$ and $f^\beta$ agree on some non-empty open subset *T* of ${\mathcal W}$ if and only if (II) $\mathrm{Cl}({\mathcal W}_{\alpha \not =\beta }) \not = {\mathcal W}$. (This is because (a) fails to hold if and only if there exists $\alpha$ and $\beta$ with $\alpha \not \equiv _{\mathcal W}\beta$ such that (I) holds; and (b) fails to hold if and only there exists $\alpha$ and $\beta$ with $\alpha \not \equiv _{\mathcal W}\beta$ and (II).) But (I) implies that ${\mathcal W}_{\alpha \not =\beta } \subseteq {\mathcal W}\setminus T$, where ${\mathcal W}\setminus T$ is closed, and thus, $\mathrm{Cl}({\mathcal W}_{\alpha \not =\beta }) \subseteq {\mathcal W}\setminus T \not = {\mathcal W}$, showing (II). Conversely, (II) implies that ${\mathcal W}{\setminus } \mathrm{Cl}({\mathcal W}_{\alpha \not =\beta })$ is a non-empty open subset on which $f^\alpha$ and $f^\beta$ agree, showing (I).

Now, using Auxiliary Lemma 3, ${\mathcal W}_A^{\not =}$ is the intersection of a finite number of open sets, i.e., $\bigcap _{\alpha ,\beta \in A: \alpha \not \equiv _{\mathcal W}\beta } {\mathcal W}_{\alpha \not =\beta }$, and thus, the equivalence of (b) and (c) follows immediately from Auxiliary Lemma 6. $\square$

#### Lemma 6

Let $A\in {\mathcal M}$ and assume, for each $\alpha \in A$, that the function $f^\alpha$ is a real-valued continuous function on the metric space ${\mathcal W}$. If ${\mathcal W}$ equals the topological closure $\mathrm{Cl}({\mathcal W}_A^{\not =})$ of ${\mathcal W}_A^{\not =}$ then ${\mathcal W}$ is $A$-Extendable.

#### Proof

Assume that $\mathrm{Cl}({\mathcal W}_A^{\not =}) = {\mathcal W}$, and consider any $w\in {\mathcal W}$. To show that ${\mathcal W}$ is $A$-Extendable, we need to show that there exists $w'$ that is total over $A$ given ${\mathcal W}$ that extends $w$ over $A$.

Let $\delta$ be the minimum value of $|f_w(\alpha ) - f_w(\beta )|$ over all $\alpha , \beta \in A$ such that $f_w(\alpha ) \not = f_w(\beta )$. Because $A$ is finite, we have $\delta > 0$. Since the topological of closure of ${\mathcal W}_A^{\not =}$ equals ${\mathcal W}$, there exists a sequence of elements of ${\mathcal W}_A^{\not =}$ that tend to $w$. In particular, there exists some $w' \in {\mathcal W}_A^{\not =}$ such that $|f_w(\alpha ) - f_{w'}(\alpha )| < \delta /3$ for all $\alpha \in A$. By definition of $\delta$, we have, for any $\alpha ,\beta \in A$, that $f_w(\alpha ) - f_w(\beta ) > 0$ implies $f_w(\alpha ) - f_w(\beta ) \ge \delta$. Then, $f_{w'}(\alpha ) - f_{w'}(\beta ) = (f_{w'}(\alpha ) - f_w(\alpha )) + (f_w(\beta ) - f_{w'}(\beta )) + (f_w(\alpha ) - f_w(\beta ))> - \frac{\delta }{3} - \frac{\delta }{3} + \delta = \frac{\delta }{3} > 0$. Thus, $f_{w'}(\alpha ) > f_{w'}(\beta )$. We have shown that, for any $\alpha ,\beta \in A$ if $\alpha \succ _w\beta$ then $\alpha \succ _{w'} \beta$; this implies that $w'$ extends $w$ over $A$, where $w'$ is total over $A$ given ${\mathcal W}$. $\square$

#### Proposition 11

Let $C\in {\mathcal M}$ and assume, for each $\alpha \in C$, that the function $f^\alpha$ is a real-valued continuous function on the metric space ${\mathcal W}$, and that the set of functions ${\{f^\alpha \, : \,\alpha \in C\}}$ satisfies the Identity property. Suppose that $A\subseteq C$. For $\alpha \in A$, we have $\alpha \in \mathrm{PSO}_{\mathcal W}(A)$ if and only if $\mathrm{Opt}_{\mathcal W}^A(\alpha )$ contains a non-empty open set, i.e., has a non-empty interior.

#### Proof

We first show that $\mathrm{Cl}({\mathcal W}') = {\mathcal W}$, where ${\mathcal W}' = \bigcup _{\gamma \in A} \mathrm{SOpt}_{\mathcal W}^A(\gamma )$, which is equal to $\bigcup _{\gamma \in \mathrm{PSO}_{\mathcal W}(A)} \mathrm{SOpt}_{\mathcal W}^A(\gamma )$, since $\mathrm{SOpt}_{\mathcal W}^A(\gamma )$ is empty if $\gamma \notin \mathrm{PSO}_{\mathcal W}(A)$. By Auxiliary Lemma 4, ${\mathcal W}_A^{\not =}\subseteq {\mathcal W}' \subseteq {\mathcal W}$, and thus, since $\mathrm{Cl}({\mathcal W}_A^{\not =}) = {\mathcal W}$ (by Lemma 5), we have $\mathrm{Cl}({\mathcal W}') = {\mathcal W}$.

Now suppose that $\alpha \in \mathrm{PSO}_{\mathcal W}(A)$. Then $\mathrm{SOpt}_{\mathcal W}^A(\alpha )$ is a non-empty subset of $\mathrm{Opt}_{\mathcal W}^A(\alpha )$, and it is open by Auxiliary Lemma 5. Conversely, suppose that $\mathrm{Opt}_{\mathcal W}^A(\alpha )$ contains a non-empty open set *T*. If $\mathrm{SOpt}_{\mathcal W}^A(\alpha )$ is non-empty then $\alpha \in \mathrm{PSO}_{\mathcal W}(A)$ and we’re done; so, to prove a contradiction, assume that $\mathrm{SOpt}_{\mathcal W}^A(\alpha )$ is empty. For $\beta$ with $\alpha \not \equiv _{\mathcal W}\beta$ we have $\mathrm{SOpt}_{\mathcal W}^A(\beta ) \cap \mathrm{Opt}_{\mathcal W}^A(\alpha ) = \emptyset$ so $\mathrm{SOpt}_{\mathcal W}^A(\beta ) \cap T = \emptyset$; this also holds for all $\beta \in A$ with $\alpha \equiv _{\mathcal W}\beta$, since for such $\beta$ we have $\mathrm{SOpt}_{\mathcal W}^A(\beta ) = \mathrm{SOpt}_{\mathcal W}^A(\alpha ) = \emptyset$. Hence, ${\mathcal W}' \cap T = \emptyset$ (recall that ${\mathcal W}' = \bigcup _{\gamma \in A} \mathrm{SOpt}_{\mathcal W}^A(\gamma )$). This implies that ${\mathcal W}\setminus T$ is a closed set containing ${\mathcal W}'$ and thus, $\mathrm{Cl}({\mathcal W}') \subseteq {\mathcal W}\setminus T$; but $\mathrm{Cl}({\mathcal W}') = {\mathcal W}$, which contradicts the assumption that *T* is non-empty. $\square$

#### Corollary 3

Assume that ${\mathcal W}$ is a convex subset of ${I\!R}^{p}$, and consider $A\in {\mathcal M}$ and assume that for each $\alpha \in A$ there exists $\hat{\alpha }\in {I\!R}^{p}$ such that for all $w\in {I\!R}^{p}$, $f_w(\alpha ) = w\cdot \hat{\alpha }$. Then $\mathrm{MPO}_{\mathcal W}(A) =\mathrm{PSO}_{\mathcal W}(A)$.

#### Proof

We always have $\mathrm{MPO}_{\mathcal W}(A) \supseteq \mathrm{PSO}_{\mathcal W}(A)$ (see Proposition 7) so it is sufficient to show that $\mathrm{MPO}_{\mathcal W}(A) \subseteq \mathrm{PSO}_{\mathcal W}(A)$, by showing that an arbitrary element $\gamma$ of $\mathrm{MPO}_{\mathcal W}(A)$ is in $\mathrm{PSO}_{\mathcal W}(A)$.

Let $A'$ consist of all $\alpha \in \mathrm{PSO}_{\mathcal W}(A)$ such that $\alpha \not \equiv _{\mathcal W}\gamma$. Corollary 2 implies that $\mathrm{PSO}_{\mathcal W}(A) \equiv _{\forall \forall \exists }^{{\mathcal W}} A$ and thus, that $\mathrm{PSO}_{\mathcal W}(A)$ is non-empty. If $A'$ were empty then we would have $\gamma \in \mathrm{PSO}_{\mathcal W}(A)$, as required. Let us now consider the case in which $A'$ is non-empty. For any $\alpha \in A'$, by definition of $\mathrm{PSO}_{\mathcal W}(A)$, we do not have $\mathrm{Opt}_{\mathcal W}^A(\alpha ) = \mathrm{Opt}_{\mathcal W}^A(\gamma )$, so, because $\gamma \in \mathrm{MPO}_{\mathcal W}(A)$, $\mathrm{Opt}_{\mathcal W}^A(\gamma ) \not \subseteq \mathrm{Opt}_{\mathcal W}^A(\alpha )$, and hence there exists $w_\alpha \in {\mathcal W}$ such that $w_\alpha \in \mathrm{Opt}_{\mathcal W}^A(\gamma ) \setminus \mathrm{Opt}_{\mathcal W}^A(\alpha )$. Let $w$ equal $\frac{1}{|A'|} \sum _{\alpha \in A'} w_\alpha$. Convexity of ${\mathcal W}$ implies that $w\in {\mathcal W}$. Consider any $\beta \in A'$. Since, for any $\alpha \in A'$, $w_\alpha \in \mathrm{Opt}_{\mathcal W}^A(\gamma )$, $w_\alpha \cdot \hat{\gamma }- w_\alpha \cdot \hat{\beta }\ge 0$, and this is strictly positive if $\alpha = \beta$, since $w_\alpha \notin \mathrm{Opt}_{\mathcal W}^A(\alpha )$. Thus, $w\cdot \hat{\gamma }- w\cdot \hat{\beta }= \frac{1}{|A'|} \sum _{\alpha \in A'} (w_\alpha \cdot \hat{\gamma }- w_\alpha \cdot \hat{\beta }) > 0$. This shows that $w\notin \mathrm{Opt}_{\mathcal W}^A(\beta )$ for all $\beta \in A'$, and $w\in \mathrm{Opt}_{\mathcal W}^A(\gamma )$, which implies that $\gamma \in \mathrm{PSO}_{\mathcal W}(B)$ where $B= \mathrm{PSO}_{\mathcal W}(A)\cup {\{\gamma \}}$. Corollary 2 implies that $\mathrm{PSO}_{\mathcal W}(A) \equiv _{\forall \forall \exists }^{{\mathcal W}} A$. Since, $\mathrm{PSO}_{\mathcal W}(A) \subseteq B\subseteq A$, Proposition 8 implies that $\mathrm{PSO}_{\mathcal W}(B) = \mathrm{PSO}_{\mathcal W}(A)$, and hence, $\gamma \in \mathrm{PSO}_{\mathcal W}(A)$, as required. $\square$

### Results in Sect. 7

We define $\textit{Filter}(A,\alpha ; \succcurlyeq _{\forall \exists \forall }^{{\mathcal W}})$ and $\textit{Filter}_\sigma (A; \succcurlyeq _{\forall \exists \forall }^{{\mathcal W}})$ analogously to $\textit{Filter}(A,\alpha ; \succcurlyeq _{\forall \forall \exists }^{{\mathcal W}})$ and $\textit{Filter}_\sigma (A; \succcurlyeq _{\forall \forall \exists }^{{\mathcal W}})$, respectively.

#### Auxiliary Lemma 7

Let $\succcurlyeq$ be either $\succcurlyeq _{\forall \forall \exists }^{{\mathcal W}}$ or $\succcurlyeq _{\forall \exists \forall }^{{\mathcal W}}$, and let $\equiv$ be the corresponding equivalence relation. Let $A, A' \in {\mathcal M}$ and let $\sigma$ be any labelling of $A$. Then we have:

(i) If $A'\subseteq A$ and $A' \succcurlyeq A\setminus A'$ then $A\equiv A'$;
(ii) for $$B\subseteq A$$, $$\textit{Filter}(A,B; \succcurlyeq ) \equiv A$$.
(iii) $$A\equiv \textit{Filter}_\sigma (A; \succcurlyeq ) \subseteq A$$.
(iv) $$\textit{Filter}_\sigma (A; \succcurlyeq _{\forall \exists \forall }^{{\mathcal W}}) \subseteq \mathrm{UD}_{\mathcal W}(A)$$.
(v) $$\textit{Filter}_\sigma (A; \succcurlyeq _{\forall \exists \forall }^{{\mathcal W}}) \equiv _{\mathcal W}\mathrm{UD}_{\mathcal W}(A)$$.
(vi) $$\text {SME}_{\mathcal W}(A) = {\{\textit{Filter}_\sigma (A; \succcurlyeq _{\forall \forall \exists }^{{\mathcal W}}) \, : \,\sigma \in \Lambda \}}$$.
(vii) $$\textit{Filter}_\sigma (A; \succcurlyeq _{\forall \forall \exists }^{{\mathcal W}}) \subseteq \mathrm{PO}_{\mathcal W}(A)$$.

#### Proof

Part (i) follows immediately using Proposition 2. (ii) follows from (i). (iii) follows from iterative application of (ii).

Regarding (iv) and (v): suppose that $\alpha _i \in A\setminus \textit{Filter}_\sigma (A; \succcurlyeq _{\forall \exists \forall }^{{\mathcal W}})$. Then, using the notation above, $A^i \not \ni \alpha _i$, i.e., $\alpha _i\notin \textit{Filter}(A^{i-1},\alpha _i; \succcurlyeq _{\forall \exists \forall }^{{\mathcal W}})$, and thus, there exists $\gamma \in A^{i-1}$ with $\gamma \succcurlyeq _{\mathcal W}\alpha$. If $\alpha _i \in \mathrm{UD}_{\mathcal W}(A)$ this implies that $\gamma \equiv _{\mathcal W}\alpha$. Applying this iteratively, we see that if $\alpha _i \in \mathrm{UD}_{\mathcal W}(A)\setminus \textit{Filter}_\sigma (A; \succcurlyeq _{\forall \exists \forall }^{{\mathcal W}})$ then there exists $\gamma \in \textit{Filter}_\sigma (A; \succcurlyeq _{\forall \exists \forall }^{{\mathcal W}})$ with $\gamma \equiv _{\mathcal W}\alpha _i$.

Now assume that $\alpha _i \in A\setminus \mathrm{UD}_{\mathcal W}(A)$; then, by Auxiliary Lemma 1, there exists $\beta \in \mathrm{UD}_{\mathcal W}(A)$ with $\beta \succ _{\mathcal W}\alpha _i$; by the above argument, there exists $\gamma \in \textit{Filter}_\sigma (A; \succcurlyeq _{\forall \exists \forall }^{{\mathcal W}})$ with $\gamma \equiv _{\mathcal W}\beta$ and thus, $\gamma \succ _{\mathcal W}\alpha _i$. We have $\gamma \in \textit{Filter}_\sigma (A; \succcurlyeq _{\forall \exists \forall }^{{\mathcal W}}) \subseteq A^{i-1}$, which implies that $\alpha _i \notin A^i$, and thus, $\alpha _i \notin \textit{Filter}_\sigma (A; \succcurlyeq _{\forall \exists \forall }^{{\mathcal W}})$. This proves (iv) $\textit{Filter}_\sigma (A; \succcurlyeq _{\forall \exists \forall }^{{\mathcal W}}) \subseteq \mathrm{UD}_{\mathcal W}(A)$.

We showed that for $\alpha \in \mathrm{UD}_{\mathcal W}(A)$ there exists $\gamma \in \textit{Filter}_\sigma (A; \succcurlyeq _{\forall \exists \forall }^{{\mathcal W}})$ with $\gamma \equiv _{\mathcal W}\alpha$; and also, $\textit{Filter}_\sigma (A; \succcurlyeq _{\forall \exists \forall }^{{\mathcal W}}) \subseteq \mathrm{UD}_{\mathcal W}(A)$. Together these imply (v): $\textit{Filter}_\sigma (A; \succcurlyeq _{\forall \exists \forall }^{{\mathcal W}}) \equiv _{\mathcal W}\mathrm{UD}_{\mathcal W}(A)$.

(vi) Firstly, we observe that if $\alpha \in \textit{Filter}_\sigma (A; \succcurlyeq )$ then $\textit{Filter}_\sigma (A; \succcurlyeq )\setminus {\{\alpha \}} \not \succcurlyeq {\{\alpha \}}$. (This follows using the fact that if $\alpha _i = \alpha$ then $A^i = \textit{Filter}(A^{i-1},{\{\alpha _i\}}; \succcurlyeq ) \ni \alpha _i$ in the sequence of sets, i.e., $A^{i-1}\setminus {\{\alpha _i\}} \not \succcurlyeq {\{\alpha _i\}}$, which, by monotonicity, implies $\textit{Filter}_\sigma (A; \succcurlyeq )\setminus {\{\alpha _i\}} \not \succcurlyeq {\{\alpha _i\}}$, since $\textit{Filter}_\sigma (A; \succcurlyeq )\subseteq A^{i-1}$.) This implies, using Proposition 2, that no strict subset of $\textit{Filter}_\sigma (A; \succcurlyeq )$ is equivalent to $A$. In particular, for the case in which $\succcurlyeq$ equals $\succcurlyeq _{\forall \forall \exists }^{{\mathcal W}}$, we obtain that $\textit{Filter}_\sigma (A; \succcurlyeq _{\forall \forall \exists }^{{\mathcal W}}) \in \text {SME}_{\mathcal W}(A)$.

Conversely, for $B\in \text {SME}_{\mathcal W}(A)$; to complete the proof of (vi) we will show that there exists $\sigma \in \Lambda$ such that $\textit{Filter}_\sigma (A; \succcurlyeq _{\forall \forall \exists }^{{\mathcal W}}) = B$. We choose $\sigma$ to list the elements of $B$ last. Now, $B\succcurlyeq _{\forall \forall \exists }^{{\mathcal W}} A$, and so, $B\succcurlyeq _{\forall \forall \exists }^{{\mathcal W}} {\{\alpha \}}$ for each $\alpha \in A\setminus B$. This implies that $\textit{Filter}_\sigma (A; \succcurlyeq _{\forall \forall \exists }^{{\mathcal W}}) \subseteq B$. Since, we have $\textit{Filter}_\sigma (A; \succcurlyeq _{\forall \forall \exists }^{{\mathcal W}}) \equiv _{\forall \forall \exists }^{{\mathcal W}} A$, and $B\in \text {SME}_{\mathcal W}(A)$, by definition of $\text {SME}_{\mathcal W}(A)$ we have $\textit{Filter}_\sigma (A; \succcurlyeq _{\forall \forall \exists }^{{\mathcal W}}) = B$.

(vii): By part (vi) it is sufficient to prove that if $B\in \text {SME}_{\mathcal W}(A)$ then $B\subseteq \mathrm{PO}_{\mathcal W}(A)$. We will show that if $\alpha \in A\setminus \mathrm{PO}_{\mathcal W}(A)$ then $B\setminus {\{\alpha \}} \equiv _{\forall \forall \exists }^{{\mathcal W}} B$ which implies $\alpha \notin B$, by definition of $\text {SME}_{\mathcal W}(A)$.

So, assume that $\alpha \notin \mathrm{PO}_{\mathcal W}(A)$. Then $\mathrm{Opt}_{\mathcal W}^A(\alpha ) = \emptyset$ so $\bigcup _{\beta \in B{\setminus }{\{\alpha \}}} \mathrm{Opt}_{\mathcal W}^A(\beta ) = \bigcup _{\beta \in B}$ $\mathrm{Opt}_{\mathcal W}^A(\beta )$, which equals ${\mathcal W}$ using Lemma 1, using the fact that $B\equiv _{\forall \forall \exists }^{{\mathcal W}} A$ (since $B\in \text {SME}_{\mathcal W}(A)$). Applying Lemma 1 again, we have that $B\setminus {\{\alpha \}} \equiv _{\forall \forall \exists }^{{\mathcal W}} B$. $\square$

Proposition 12 immediately follows, using Auxiliary Lemma 7, and with part (iii) also using Theorem 1. More specifically, part (i) is implied by Auxiliary Lemma 7(iii); part (ii) is Auxiliary Lemma 7(vi); and, under the assumptions of part (iii), Theorem 1 implies that $\text {SME}_{\mathcal W}(A)= {\{\mathrm{PSO}_{\mathcal W}(A)\}}$, so part (ii) then implies that $\textit{Filter}_\sigma (A; \succcurlyeq _{\forall \forall \exists }^{{\mathcal W}}) = \mathrm{PSO}_{\mathcal W}(A)$ for every labelling $\sigma$.

#### Lemma 7

Let ${\mathcal W}$ ($\subseteq {\mathcal U}$) be a set of scenarios and let $A\in {\mathcal M}$. $\mathrm{MPO}_{\mathcal W}(A)$ is the set of $\mathrm{Opt}_{\mathcal W}^A$-undominated elements (of $A$). $\mathrm{Opt}_{\mathcal W}^A$-dominance is an irreflexive transitive relation on $A$, and if $\alpha$ is $\mathrm{Opt}_{\mathcal W}^A$-dominated then there exists $\mathrm{Opt}_{\mathcal W}^A$-undominated $\beta$ that $\mathrm{Opt}_{\mathcal W}^A$-dominates $\alpha$.

#### Proof

$\alpha \in \mathrm{MPO}_{\mathcal W}(A)$ if and only if there does not exist $\beta \in A$ with $\mathrm{Opt}_{\mathcal W}^A(\beta ) \supsetneqq \mathrm{Opt}_{\mathcal W}^A(\alpha )$, i.e., $\alpha$ is $\mathrm{Opt}_{\mathcal W}^A$-undominated. The definition immediately implies that $\mathrm{Opt}_{\mathcal W}^A$-dominance is an irreflexive transitive relation. Together with finiteness of $A$ this implies that if $\alpha$ is $\mathrm{Opt}_{\mathcal W}^A$-dominated then there exists $\mathrm{Opt}_{\mathcal W}^A$-undominated $\beta$ that $\mathrm{Opt}_{\mathcal W}^A$-dominates $\alpha$. $\square$

### Results in Sect. 8

Proposition 14 and Proposition 15 follow immediately from parts of the following lemma.

#### Auxiliary Lemma 8

(i) $\textit{SMR}_{\mathcal W}(A, B)$ is monotonically decreasing in $A$, monotonically increasing in $B$ and monotonically increasing in ${\mathcal W}$, i.e., if $A' \supseteq A$, and $B' \subseteq B$, and ${\mathcal W}' \subseteq {\mathcal W}$ then $\textit{SMR}_{{\mathcal W}'}(A', B') \le \textit{SMR}_{\mathcal W}(A, B)$.
(ii) $$\textit{SMR}_{\mathcal W}(A, B) = \max _{\beta \in B} \textit{SMR}_{\mathcal W}(A, {\{\beta \}})$$
(iii) $\textit{SMR}_{\mathcal W}(A, B) \le 0$ if and only if $A\succcurlyeq _{\forall \forall \exists }^{{\mathcal W}} B$.
(iv) If $\textit{SMR}_{\mathcal W}(A, B)$ is achieved then $\textit{SMR}_{\mathcal W}(A, B) \ge 0$ if and only if $\mathrm{PO}_{\mathcal W}(A\cup B) \cap B\not =\emptyset$.
(v) If $A' \equiv _{\forall \forall \exists }^{{\mathcal W}} A$ and $B' \equiv _{\forall \forall \exists }^{{\mathcal W}} B$ then $\textit{SMR}_{\mathcal W}(A', B') = \textit{SMR}_{\mathcal W}(A, B)$.
(vi) If $A'\subseteq A$ and $B'\subseteq B$ and $A' \succcurlyeq _{\forall \forall \exists }^{{\mathcal W}} A\setminus A'$ and $B' \succcurlyeq _{\forall \forall \exists }^{{\mathcal W}} B\setminus B'$ then $\textit{SMR}_{\mathcal W}(A', B') = \textit{SMR}_{\mathcal W}(A, B)$.
(vii) If $B'\subseteq B$ and $A\succcurlyeq _{\forall \forall \exists }^{{\mathcal W}} B\setminus B'$ and $\textit{SMR}_{\mathcal W}(A, B) \ge 0$ then $\textit{SMR}_{\mathcal W}(A, B') = \textit{SMR}_{\mathcal W}(A, B)$.
(viii) $$\textit{SMR}_{\mathcal W}(A, B) = \max _{\alpha \in \mathrm{PO}_{\mathcal W}(A)} \textit{SMR}_{\mathrm{Opt}_{\mathcal W}^A(\alpha )}({\{\alpha \}}, B)$$.
(ix) For equivalence-free $A$, and $\alpha \in A$, $\textit{SMR}_{\mathcal W}(A\setminus {\{\alpha \}}, {\{\alpha \}}) > 0$ if and only if $\alpha \in \mathrm{PSO}_{\mathcal W}(A)$.

#### Proof

It is convenient to note that $\textit{SMR}_{\mathcal W}(A, B)$ can be written as $\sup _{w\in {\mathcal W}} \textit{Reg}_w(A,B)$, where $\textit{Reg}_w(A,B)$ is defined to be $\max _{\beta \in B} f_w(\beta ) - \max _{\alpha \in A} f_w(\alpha )$.

(i): The fact that $A' \supseteq A$ and $B' \subseteq B$ implies that for all $w\in {\mathcal U}$, $\textit{Reg}_w(A',B') \le \textit{Reg}_w(A,B)$. Then $\textit{SMR}_{{\mathcal W}'}(A, B) = \sup _{w\in {\mathcal W}'} \textit{Reg}_w(A',B') \le \sup _{w\in {\mathcal W}} \textit{Reg}_w(A',B') \le \sup _{w\in {\mathcal W}} \textit{Reg}_w(A,B) = \textit{SMR}_{\mathcal W}(A, B)$.

(ii): $\max _{\beta \in B} \textit{SMR}_{\mathcal W}(A, {\{\beta \}}) = \max _{\beta \in B} \sup _{w\in {\mathcal W}} \textit{Reg}_w(A,{\{\beta \}})$, which equals $\sup _{w\in {\mathcal W}}$ $\max _{\beta \in B}$ $\textit{Reg}_w(A,{\{\beta \}}) = \sup _{w\in {\mathcal W}} \textit{Reg}_w(A,B) = \textit{SMR}_{\mathcal W}(A, B)$.

(iii): $\textit{SMR}_{\mathcal W}(A, B) \le 0$, i.e., $\sup _{w\in {\mathcal W}} \textit{Ut}_B(w) - \textit{Ut}_A(w) \le 0$, if and only if $\textit{Ut}_B(w) \le \textit{Ut}_A(w)$ for all $w\in {\mathcal W}$, which is if and only if $A\succcurlyeq _{\forall \forall \exists }^{{\mathcal W}} B$, by Proposition 3.

Regarding (iv): Assume that $\textit{SMR}_{\mathcal W}(A, B)$ is achieved; then $\textit{SMR}_{\mathcal W}(A, B) \ge 0$ if and only if there exists $w\in {\mathcal W}$ such that $\textit{Reg}_w(A,B) \ge 0$. Now, $\textit{Reg}_w(A,B) \ge 0$ if and only if $\max _{\beta \in B} f_w(\beta ) - \max _{\alpha \in A} f_w(\alpha ) \ge 0$, which is if and only if there exists $\beta \in B$ such that for all $\gamma \in A\cup B$, $f_w(\beta ) \ge f_w(\gamma )$. This implies that $\textit{Reg}_w(A,B) \ge 0$ if and only if i.e., $B\cap \mathrm{O}_w(A\cup B)$ is non-empty. Thus, $\textit{SMR}_{\mathcal W}(A, B) \ge 0$ if and only if $B\cap \bigcup _{w\in {\mathcal W}} \mathrm{O}_w(A\cup B)$ is non-empty, i.e., $\mathrm{PO}_{\mathcal W}(A\cup B) \cap B\not =\emptyset$.

(v) follows because $\textit{SMR}_{\mathcal W}$ just depends on the utility function. This then implies (vi), using Proposition 2. Regarding (vii): Assume that $B'\subseteq B$ and $A\succcurlyeq _{\forall \forall \exists }^{{\mathcal W}} B\setminus B'$ and $\textit{SMR}_{\mathcal W}(A, B) \ge 0$. (ii) implies that $\textit{SMR}_{\mathcal W}(A, B) = \max (\textit{SMR}_{\mathcal W}(A, B'), \textit{SMR}_{\mathcal W}(A, B\setminus B'))$, and (iii) implies that $\textit{SMR}_{\mathcal W}(A, B\setminus B') \le 0$. Thus, $\textit{SMR}_{\mathcal W}(A, B') \ge \textit{SMR}_{\mathcal W}(A, B\setminus B')$ and so $\textit{SMR}_{\mathcal W}(A, B') = \textit{SMR}_{\mathcal W}(A, B)$.

(viii): $\textit{SMR}_{\mathcal W}(A, B) = \sup _{w\in {\mathcal W}} \textit{Ut}_B(w) - \textit{Ut}_A(w)$, which can be written as $\max _{\alpha \in \mathrm{PO}_{\mathcal W}(A)}$ $\sup _{w\in \mathrm{Opt}_{\mathcal W}^A(\alpha )} \textit{Ut}_B(w) - \textit{Ut}_A(w)$, since $\bigcup _{\alpha \in \mathrm{PO}_{\mathcal W}(A)} \mathrm{Opt}_{\mathcal W}^A(\alpha ) = \bigcup _{\alpha \in A} \mathrm{Opt}_{\mathcal W}^A(\alpha ) = {\mathcal W}$, using Lemma 1. For $w\in \mathrm{Opt}_{\mathcal W}^A(\alpha )$, $\textit{Ut}_A(w) = f_w(\alpha )$, so $\sup _{w\in \mathrm{Opt}_{\mathcal W}^A(\alpha )} \textit{Ut}_B(w) - \textit{Ut}_A(w) = \sup _{w\in \mathrm{Opt}_{\mathcal W}^A(\alpha )} \textit{Ut}_B(w) - f_w(\alpha )$, which equals $\textit{SMR}_{\mathrm{Opt}_{\mathcal W}^A(\alpha )}({\{\alpha \}}, B)$, showing the result.

(ix): Consider equivalence-free $A$, and $\alpha \in A$. Then, $\textit{SMR}_{\mathcal W}(A\setminus {\{\alpha \}}, {\{\alpha \}}) > 0$ $\iff$ $\sup _{w\in {\mathcal W}} f_w(\alpha ) - \textit{Ut}_{A\setminus {\{\alpha \}}}(w) > 0$, which is if and only if there exists $w\in {\mathcal W}$ such that for all $\gamma \in A\setminus {\{\alpha \}}$, $f_w(\alpha ) > f_w(\gamma )$, which, since $A$ is equivalence-free, holds if and only if $\mathrm{PSO}_{\mathcal W}(A) \ni \alpha$. $\square$

#### Lemma 8

Consider any $$A,B\in {\mathcal M}$$.

(i) If $A' \equiv _{\forall \forall \exists }^{{\mathcal W}} A$ and $B' \equiv _{\forall \forall \exists }^{{\mathcal W}} B$ then $\textit{SMR}_{\mathcal W}(A', B') = \textit{SMR}_{\mathcal W}(A, B)$.
(ii) $$\textit{SMR}_{\mathcal W}(\mathrm{UD}_{\mathcal W}(A), \mathrm{UD}_{\mathcal W}(B)) = \textit{SMR}_{\mathcal W}(A, B)$$.
(iii) If $B'\subseteq B$ and $A\succcurlyeq _{\forall \exists \forall }^{{\mathcal W}} B\setminus B'$ and $\textit{SMR}_{\mathcal W}(A, B) \ge 0$ then $\textit{SMR}_{\mathcal W}(A, B') = \textit{SMR}_{\mathcal W}(A, B)$.

#### Proof

(i) follows immediately from Auxiliary Lemma 8(v).

(ii): By Proposition 5, $\mathrm{UD}_{\mathcal W}(A) \equiv _{\forall \forall \exists }^{{\mathcal W}} A$ and $\mathrm{UD}_{\mathcal W}(B) \equiv _{\forall \forall \exists }^{{\mathcal W}} B$. Part (i) then implies the result.

(iii): Assume $B'\subseteq B$ and $A\succcurlyeq _{\forall \exists \forall }^{{\mathcal W}} B\setminus B'$ and $\textit{SMR}_{\mathcal W}(A, B) \ge 0$. Auxiliary Lemma 8(ii) implies $\textit{SMR}_{\mathcal W}(A, B) = \max (\textit{SMR}_{\mathcal W}(A, B'), \textit{SMR}_{\mathcal W}(A, B\setminus B'))$. Because $A\succcurlyeq _{\forall \exists \forall }^{{\mathcal W}} B\setminus B'$, we have by Auxiliary Lemma 8(iii) that $\textit{SMR}_{\mathcal W}(A, B\setminus B') \le 0$. Thus, $\textit{SMR}_{\mathcal W}(A, B\setminus B') \le \textit{SMR}_{\mathcal W}(A, B')$ and therefore, $\textit{SMR}_{\mathcal W}(A, B') = \textit{SMR}_{\mathcal W}(A, B)$. $\square$

### Results in Sect. 9

#### Proposition 16

Consider $$A\in {\mathcal M}$$ and $${\mathcal W}\subseteq {I\!R}^{p}$$.

(i) $\alpha$ is a feasible answer to query $A$ given ${\mathcal W}$ if and only if $\alpha \in \mathrm{PO}_{\mathcal W}(A)$.
(ii) If the set of functions ${\{f^\alpha \, : \,\alpha \in A\}}$ satisfies the Identity property, we have that $\alpha$ is a strongly feasible answer to query $A$ given ${\mathcal W}$ if and only if $\alpha \in \mathrm{PSO}_{\mathcal W}(A)$.

#### Proof

(i): $\alpha$ is a feasible answer to query $A$ given ${\mathcal W}$ if and only if $\mathrm{Opt}_{\mathcal W}^A(\alpha )$ is non-empty, i.e., $\alpha \in \mathrm{PO}_{\mathcal W}(A)$.

(ii) follows immediately from Proposition 11. $\square$

### Results in Sect. 10

We first give two basic lemmas involving the set $J_{\mathcal W}^\alpha$ defined below.

#### Definition 17

For any ${\mathcal W}\subseteq {\mathcal U}$, and any $\alpha \in {I\!R}^{p}$ we define $J_{\mathcal W}^\alpha$ to be the set $\{(w,r) \, : \,$ $w\in {\mathcal W}\  \& \ r \ge f_w(\alpha )\}$.

#### Auxiliary Lemma 9

(i) If ${\mathcal W}$ is closed and $f_w(\alpha )$ is a continuous function of $w$ then $J_{\mathcal W}^\alpha$ is a closed subset of ${I\!R}^{p}\times {I\!R}$.
(ii) If ${\mathcal W}$ is convex and $f_w(\alpha )$ is a convex function of $w$ then $J_{\mathcal W}^\alpha$ is convex. ($f_w(\alpha )$ is a convex function means that for any $s \in (0,1)$, and any $w_1, w_2 \in {I\!R}^{p}$, $f_w(\alpha ) \le sf_{w_1}(\alpha ) + (1-s)f_{w_2}(\alpha )$ where $w= sw_1 + (1-s)w_2$.)

#### Proof

(i): Showing $J_{\mathcal W}^\alpha$ is closed if ${\mathcal W}$ is closed and $f_w(\alpha )$ is continuous: Suppose $(w,r)\notin J_{\mathcal W}^\alpha$. Either (a) $w\notin {\mathcal W}$ or (b) $r < f_w(\alpha )$. If (a) $w\notin {\mathcal W}$ then the fact that ${\mathcal W}$ is closed implies that there’s an open set *S* in ${I\!R}^{p}$ containing $w$ and such that $S \cap {\mathcal W}= \emptyset$. Let $S' = S \times (a, b)$ for any open interval (*a*, *b*) of ${I\!R}$ containing *r*. Then $S'$ is an open ball in ${I\!R}^{p}\times {I\!R}$, and $(w,r) \in S'$ and $S' \cap J_{\mathcal W}^\alpha = \emptyset$.

If (b) $r < f_w(\alpha )$: let $\epsilon = \frac{1}{3}(f_w(\alpha ) - r)$. By continuity of $f_w(\alpha )$, there exists $\delta > 0$ such that for all $w' \in {I\!R}^{p}$ with $|w' - w| < \delta$ we have $|f_{w'}(\alpha ) - f_w(\alpha )| < \epsilon$. Let $S_\delta = {\{w' \in {I\!R}^{p}\, : \,|w' - w| < \delta \}}$. Let $S'' = S_\delta \times (r-\epsilon , r+\epsilon )$ which contains $(w,r)$. For any $(w', r') \in S''$, $|f_{w'}(\alpha ) - f_w(\alpha )| < \epsilon$ and $|r' - r| < \epsilon$ so $|f_{w'}(\alpha ) - r'| > \epsilon$, since $|f_{w}(\alpha ) - r| = 3\epsilon$. Thus, $S''$ is an open set containing $(w,r)$ that is disjoint from $J_{\mathcal W}^\alpha$.

We’ve shown that for any $(w,r)\notin J_{\mathcal W}^\alpha$ there exists an open set containing $(w,r)$ that is disjoint from $J_{\mathcal W}^\alpha$, which proves that $({I\!R}^{p}\times {I\!R}) \setminus J_{\mathcal W}^\alpha$ is an open subset of ${I\!R}^{p}\times {I\!R}$, and thus, $J_{\mathcal W}^\alpha$ is a closed subset.

(ii): Showing $J_{\mathcal W}^\alpha$ is convex if ${\mathcal W}$ is convex and $f_w(\alpha )$ is a convex function: Consider any $(w_1, r_1), (w_2, r_2)\in J_{\mathcal W}^\alpha$, let $s \in (0,1)$ and let $(w_0, r_0) = s(w_1, r_1) + (1-s)(w_2, r_2)$, so that $w_0 = sw_1 + (1-s)w_2$ and $r_0 = sr_1 + (1-s)r_2$. Since, $(w_1, r_1), (w_2, r_2)\in J_{\mathcal W}^\alpha$ we have $r_1 \ge f_{w_1}(\alpha )$ and $r_2 \ge f_{w_2}(\alpha )$. Since $f_w(\alpha )$ is a convex function of $w$ we have $f_{w_0}(\alpha ) \le sf_{w_1}(\alpha ) + (1-s)f_{w_2}(\alpha ) \le sr_1 + (1-s)r_2 = r_0$. Thus, $(w_0, r_0) \in J_{\mathcal W}^\alpha$, proving that $J_{\mathcal W}^\alpha$ is convex. $\square$

#### Auxiliary Lemma 10

Consider any finite subset $A$ of ${I\!R}^{p}$, and any ${\mathcal W}\subseteq {\mathcal U}$, and any $\alpha \in {I\!R}^{p}$. Then:

(i) $$\Gamma ({\mathcal W},{\{\alpha \}}) = J_{\mathcal W}^\alpha $$;
(ii) $$\Gamma ({\mathcal W},A) = \bigcap _{\alpha \in A} \Gamma ({\mathcal W},{\{\alpha \}})= \bigcap _{\alpha \in A} J_{\mathcal W}^\alpha $$.
(iii) If ${\mathcal W}$ is closed and for all $\alpha \in A$, $f_w(\alpha )$ is continuous then $\Gamma ({\mathcal W},A)$ is closed.
(iv) If ${\mathcal W}$ is convex and for all $\alpha \in A$, $f_w(\alpha )$ is a convex function of $w$ then $\Gamma ({\mathcal W},A)$ is convex.
(v) If ${\mathcal W}$ is a convex polytope and for $w\in {I\!R}^{p}, \alpha \in {I\!R}^{p}$, $f_w(\alpha ) = w\cdot \hat{\alpha }$ then $\Gamma ({\mathcal W},A)$ is a convex polytope.

#### Proof

(i): $(w,r) \in \Gamma ({\mathcal W},{\{\alpha \}})$ if and only if $w\in {\mathcal W}$ and $r \ge \textit{Ut}_{{\{\alpha \}}}(w) = f_w(\alpha )$, so $\Gamma ({\mathcal W},{\{\alpha \}}) = J_{\mathcal W}^\alpha$.

(ii): Given $w\in {\mathcal W}$, we have $(w,r) \in \Gamma ({\mathcal W},A)$ if and only if and $r \ge \textit{Ut}_{A}(w) = \max _{\alpha \in A} f_w(\alpha )$ if and only if for all $\alpha \in A$, $r \ge f_w(\alpha )$, which, by part (i), is if and only if for all $\alpha \in A$, $(w,r) \in \Gamma ({\mathcal W},{\{\alpha \}})$. Thus, $\Gamma ({\mathcal W},A) = \bigcap _{\alpha \in A} \Gamma ({\mathcal W},{\{\alpha \}})= \bigcap _{\alpha \in A} J_{\mathcal W}^\alpha$.

(iii): Assume that ${\mathcal W}$ is closed and for all $\alpha \in A$, $f_w(\alpha )$ is continuous. Auxiliary Lemma 9(i) implies that for all $\alpha \in A$, $J_{\mathcal W}^\alpha$ is closed, which implies that $\Gamma ({\mathcal W},A)$ is closed using (ii), since an intersection of closed sets is closed.

(iv): Assume that ${\mathcal W}$ is convex and for all $\alpha \in A$, $f_w(\alpha )$ is a convex function of $w$. Then Auxiliary Lemma 9(ii) implies that for all $\alpha \in A$, $J_{\mathcal W}^\alpha$ is convex, which implies that $\Gamma ({\mathcal W},A)$ is convex using (ii), since an intersection of convex sets is convex.

(v): Assume that ${\mathcal W}$ is a convex polytope and for $w\in {I\!R}^{p}, \alpha \in {I\!R}^{p}$, $f_w(\alpha ) = w\cdot \hat{\alpha }$. Then, for each $\alpha \in A$, $J_{\mathcal W}^\alpha$ is the intersection of ${\mathcal W}\times {I\!R}$ with a closed half-space, and is thus a convex polytope. Therefore, using (ii), $\Gamma ({\mathcal W},A)$ is a convex polytope, since it is the intersection of a finite number of convex polytopes. $\square$

The following technical lemma is important in proving Auxiliary Lemmas 12 and 13 and thus Theorem 4.

#### Definition 18

Suppose that $A$ is a finite subset of ${I\!R}^{p}$, and ${\mathcal W}$ is a compact subset of ${I\!R}^{p}$, and that for all $\alpha \in A$, $f_w(\alpha )$ is a continuous function of $w\in {\mathcal W}$. Define $\textit{MaxUt}_A^{\mathcal W}$ to be $\max _{\alpha \in A} \sup _{w\in {\mathcal W}} f_w(\alpha )$.

Note that the assumed conditions ensure that $\textit{MaxUt}_A^{\mathcal W}$ is finite, because a continuous function on a compact set is bounded. $\textit{MaxUt}_A^{\mathcal W}$ is also equal to $\sup _{w\in {\mathcal W}} \textit{Ut}_{A}(w)$.

For $E\subseteq {I\!R}^{p}$ we let $\textit{CH}(E)$ be the convex hull of *E*.

#### Auxiliary Lemma 11

Assume that ${\mathcal W}$ is a compact and convex subset of ${I\!R}^{p}$, and for all $\alpha \in A$, $f_w(\alpha )$ is a convex and continuous function of $w\in {\mathcal W}$. There exists real value *N* such that for all $w\in {\mathcal W}$, $N > \textit{Ut}_{A}(w)$; in particular, this holds for any $N > \textit{MaxUt}_A^{\mathcal W}$. Define $\Gamma _{\! N}({\mathcal W},A)$ to be the intersection of $\Gamma ({\mathcal W},A)$ with the half-space ${\{(w,r) \, : \,r \le N\}}$. Assume that ${\mathcal W}$ is a convex polytope. Then

(i) $\Gamma _{\! N}({\mathcal W},A)$ is compact and convex.
(ii) $$\textit{CH}(\textit{Ext}(\Gamma _{\! N}({\mathcal W},A))) = \Gamma _{\! N}({\mathcal W},A)$$.
(iii) If $(w, r) \in \textit{Ext}(\Gamma ({\mathcal W},A))$ then $r = \textit{Ut}_{A}(w)$.
(iv) $$\textit{Ext}(\Gamma ({\mathcal W},A)) \subseteq \Gamma _{\! N}({\mathcal W},A)$$.
(v) $$\textit{Ext}(\Gamma ({\mathcal W},A)) \subseteq \textit{Ext}(\Gamma _{\! N}({\mathcal W},A))$$.
(vi) If $(w,r) \in \textit{Ext}(\Gamma _{\! N}({\mathcal W},A)) {\setminus } \textit{Ext}(\Gamma ({\mathcal W},A))$ then $r = N$.

#### Proof

Firstly, $\textit{Ut}_{A}(w)$ is a continuous function on compact set ${\mathcal W}$ so is bounded; we can therefore choose *N* and *M* such that for all $w\in {\mathcal W}$, $M< \textit{Ut}_{A}(w) < N$. Since for all $w\in {\mathcal W}$, $\textit{Ut}_{A}(w) \le \textit{MaxUt}_A^{\mathcal W}$, if $N' > \textit{MaxUt}_A^{\mathcal W}$ then for all $w\in {\mathcal W}$, $\textit{Ut}_{A}(w) < N'$.

(i) $\Gamma _{\! N}({\mathcal W},A)$ is bounded and thus compact, since it is a subset of the cylinder ${\mathcal W}\times [M,N]$, and ${\mathcal W}$ is compact. $\Gamma _{\! N}({\mathcal W},A)$ is therefore compact and convex. Using a well-known property of extreme points (e.g., the Krein-Milman theorem), this implies (ii) $\textit{CH}($ $\textit{Ext}(\Gamma _{\! N}({\mathcal W},A))) = \Gamma _{\! N}({\mathcal W},A)$.

(iii) immediately follows from the fact that the line ${\{(w, r') \, : \,r' \ge \textit{Ut}_{A}(w) \}}$ is a subset of $\Gamma ({\mathcal W},A)$. Since, we have $N > \textit{Ut}_{A}(w)$, this implies (iv) $\textit{Ext}(\Gamma ({\mathcal W},A)) \subseteq \Gamma _{\! N}({\mathcal W},A)$. This also implies (v) $\textit{Ext}(\Gamma ({\mathcal W},A)) \subseteq \textit{Ext}(\Gamma _{\! N}({\mathcal W},A))$, since if it were the case that $(w,r) \in \textit{Ext}(\Gamma ({\mathcal W},A)) \setminus \textit{Ext}(\Gamma _{\! N}({\mathcal W},A))$ then there would exist a line segment, containing $(w,r)$ as an internal point, within $\Gamma _{\! N}({\mathcal W},A)$, and thus, within $\Gamma ({\mathcal W},A)$, which contradicts $(w,r) \in \textit{Ext}(\Gamma ({\mathcal W},A))$.

(vi): Assume that $(w,r) \in \textit{Ext}(\Gamma _{\! N}({\mathcal W},A)) \setminus \textit{Ext}(\Gamma ({\mathcal W},A))$. Now, $(w,r) \in \Gamma ({\mathcal W},A) \setminus \textit{Ext}(\Gamma ({\mathcal W},A))$, so there exists a line segment *L* within $\Gamma ({\mathcal W},A)$ which has $(w,r)$ as an internal point. Let $L'$ be the line segment intersected with the half-space ${\{(w,r') \, : \,r' \le N\}}$. Then $L' \subseteq \Gamma _{\! N}({\mathcal W},A)$. Since $(w,r) \in \textit{Ext}(\Gamma _{\! N}({\mathcal W},A))$, $(w,r)$ cannot be an internal point of $L'$, (and $r \not = \textit{Ut}_{A}(w)$ because $(w,\textit{Ut}_{A}(w))$ is not an internal point of *L*) and so *r* must equal *N*. $\square$

#### Auxiliary Lemma 12

Assume that ${\mathcal W}$ is a compact and convex subset of ${I\!R}^{p}$, and for all $\alpha \in A$, $f_w(\alpha )$ is a convex and continuous function of $w\in {\mathcal W}$. Let $\Theta ({\mathcal W},A) = \{(w,\textit{Ut}_{A}(w)) \, : \,$ $w\in {\mathcal W}\}$. Then $\textit{CH}(\textit{Ext}(\Gamma ({\mathcal W},A)))$ contains $\Theta ({\mathcal W},A)$, i.e., for all $w\in {\mathcal W}$, $\textit{CH}(\textit{Ext}(\Gamma ({\mathcal W},A)))$ contains $(w,\textit{Ut}_{A}(w))$.

#### Proof

Consider any $w\in {\mathcal W}$. By definition, $(w,\textit{Ut}_{A}(w)) \in \Gamma ({\mathcal W},A)$. Choose any $N > \textit{MaxUt}_A^{\mathcal W}$; by Auxiliary Lemma 11, for all $w\in {\mathcal W}$, $N > \textit{Ut}_{A}(w)$, and $(w,\textit{Ut}_{A}(w)) \in \Gamma _{\! N}({\mathcal W},A)$. Using Auxiliary Lemma 11(ii), $\textit{CH}(\textit{Ext}(\Gamma _{\! N}({\mathcal W},A))) \ni (w,\textit{Ut}_{A}(w))$ so we can write $(w,\textit{Ut}_{A}(w))$ as $\sum _{j=1}^J \tau _j q_j$ where $q_j \in \textit{Ext}(\Gamma _{\! N}({\mathcal W},A))$, and the $\tau _j$ are non-negative reals that sum to 1. We will show that $\tau _j = 0$ unless $q_j \in \textit{Ext}(\Gamma ({\mathcal W},A))$. This then implies that $(w,\textit{Ut}_{A}(w)) \in \textit{CH}(\textit{Ext}(\Gamma ({\mathcal W},A)))$, proving Auxiliary Lemma 12.

So, suppose that there exists *k* with $\tau _k > 0$ and $q_k \in \textit{CH}(\textit{Ext}(\Gamma _{\! N}({\mathcal W},A))) \setminus \textit{Ext}(\Gamma ({\mathcal W},A))$. By Auxiliary Lemma 11(vi), $q_k = (w', N)$ for some $w'\in {\mathcal W}$ and $N > \textit{Ut}_{A}(w')$. Let $q' = \sum _{j=1}^J \tau _j q'_j$, where $q'_k = (w', \textit{Ut}_{A}(w'))$, and $q'_j = q_j$ for $j \not = k$. Then for all *j*, $q'_j \in \Gamma ({\mathcal W},A)$ and so, by convexity of $\Gamma ({\mathcal W},A)$, $q' \in \Gamma ({\mathcal W},A)$. Also, $q'$ can be written as $(w, r')$ for some $r'$, and $r' < \textit{Ut}_{A}(w)$ (see below). The definition of $\Gamma ({\mathcal W},A)$ implies that $(w, r') \notin \Gamma ({\mathcal W},A)$, giving the required contradiction.

In more detail, we have $(w,\textit{Ut}_{A}(w)) - q' = \sum _{j=1}^J \tau _j (q_j - q'_j) = \tau _k (q_k - q'_k) = \tau _k ((w', N) - (w', \textit{Ut}_{A}(w'))) = \tau _k (0, N - \textit{Ut}_{A}(w'))$. Then, $q' = (w, r')$ where $r' = \textit{Ut}_{A}(w) - \tau _k(N - \textit{Ut}_{A}(w')) < \textit{Ut}_{A}(w)$. $\square$

The following result states that $\Gamma ({\mathcal W},A)$ is determined by its extreme points, even though it is not compact.

#### Auxiliary Lemma 13

Consider any finite subsets $A$ and $B$ of ${I\!R}^{p}$, and any compact and convex subset ${\mathcal W}$ of ${I\!R}^{p}$, and assume that for all $\alpha \in A\cup B$, $f_w(\alpha )$ is a convex and continuous function of $w\in {\mathcal W}$. Then $\Gamma ({\mathcal W},A) = \Gamma ({\mathcal W},B)$ $\iff$ $\textit{Ext}(\Gamma ({\mathcal W},A)) = \textit{Ext}(\Gamma ({\mathcal W},B))$.

#### Proof

If $\Gamma ({\mathcal W},A) = \Gamma ({\mathcal W},B)$ then obviously $\textit{Ext}(\Gamma ({\mathcal W},A)) = \textit{Ext}(\Gamma ({\mathcal W},B))$. Regarding the converse, assume that $\textit{Ext}(\Gamma ({\mathcal W},A)) = \textit{Ext}(\Gamma ({\mathcal W},B))$. For any $w\in {\mathcal W}$, $(w,\textit{Ut}_{A}(w)) \in \Gamma ({\mathcal W},A)$ and $(w,r) \in \Gamma ({\mathcal W},A)$ if and only if $r \ge \textit{Ut}_{A}(w)$. Now, Auxiliary Lemma 12 implies that $(w,\textit{Ut}_{B}(w))$ is in the convex hull of $\textit{Ext}(\Gamma ({\mathcal W},B))$, and thus, in the convex hull of $\textit{Ext}(\Gamma ({\mathcal W},A))$; hence, by convexity of $\Gamma ({\mathcal W},A)$, we have $(w,\textit{Ut}_{B}(w)) \in \Gamma ({\mathcal W},A)$, which shows that $\textit{Ut}_{B}(w)\ge \textit{Ut}_{A}(w)$, which holds for an arbitrary element $w$ of ${\mathcal W}$. This implies $\Gamma ({\mathcal W},A) \supseteq \Gamma ({\mathcal W},B)$. Switching the roles of $A$ and $B$ in the argument shows also $\Gamma ({\mathcal W},A) \subseteq \Gamma ({\mathcal W},B)$, and thus, $\Gamma ({\mathcal W},A) = \Gamma ({\mathcal W},B)$. $\square$

#### Theorem 4

Consider any finite subsets $A$ and $B$ of ${I\!R}^{p}$, any $\beta \in {I\!R}^{p}$, and any compact and convex subset ${\mathcal W}$ of ${I\!R}^{p}$, and assume that for all $\alpha \in A\cup B\cup {\{\beta \}}$, $f_w(\alpha )$ is a convex and continuous function of $w\in {\mathcal W}$.

(i) $$A\succcurlyeq _{\forall \forall \exists }^{{\mathcal W}} B$$ $$\iff $$ $$\Gamma ({\mathcal W},A) \subseteq \Gamma ({\mathcal W},B)$$ $$\iff $$ $$\Gamma ({\mathcal W},A) = \Gamma ({\mathcal W},A\cup B)$$.
(ii) $A\succcurlyeq _{\forall \forall \exists }^{{\mathcal W}} B$ if and only if $\textit{Ext}(\Gamma ({\mathcal W},A)) = \textit{Ext}(\Gamma ({\mathcal W},A\cup B))$.
(iii) $A\succcurlyeq _{\forall \forall \exists }^{{\mathcal W}} B$ holds if and only if for all $(w,r) \in \textit{Ext}(\Gamma ({\mathcal W},A))$ and for all $\beta \in B$ we have $f_w(\beta ) \le r$.
(iv) $$\textit{SMR}_{\mathcal W}(A, {\{\beta \}}) = \max {\{f_w(\beta ) - r \, : \,(w,r) \in \textit{Ext}(\Gamma ({\mathcal W},A)) \}}$$.

#### Proof

Regarding (i): Proposition 3 implies that $A\succcurlyeq _{\forall \forall \exists }^{{\mathcal W}} B$ $\iff$ for all $w\in {\mathcal W}$, $\textit{Ut}_A(w) \ge \textit{Ut}_B(w)$, which is if and only if $\Gamma ({\mathcal W},A) \subseteq \Gamma ({\mathcal W},B)$. Using Auxiliary Lemma 10, $\Gamma ({\mathcal W},A\cup B) = \Gamma ({\mathcal W},A) \cap \Gamma ({\mathcal W},B)$. This implies $\Gamma ({\mathcal W},A) \subseteq \Gamma ({\mathcal W},B)$ $\iff$ $\Gamma ({\mathcal W},A) = \Gamma ({\mathcal W},A\cup B)$.

(ii): By (i), $A\succcurlyeq _{\forall \forall \exists }^{{\mathcal W}} B$ if and only if $\Gamma ({\mathcal W},A) = \Gamma ({\mathcal W},A\cup B)$, which, by Auxiliary Lemma 13 is if and only if $\textit{Ext}(\Gamma ({\mathcal W},A)) = \textit{Ext}(\Gamma ({\mathcal W},A\cup B))$.

(iii): Using (i), $A\succcurlyeq _{\forall \forall \exists }^{{\mathcal W}} {\{\beta \}}$ holds if and only if $\Gamma ({\mathcal W},A) \subseteq \Gamma ({\mathcal W},{\{\beta \}})$. Now, $\Gamma ({\mathcal W},A) \subseteq \Gamma ({\mathcal W},{\{\beta \}})$ $\iff$ $\Theta ({\mathcal W},A) \subseteq \Gamma ({\mathcal W},{\{\beta \}})$ which is if and only if $\textit{CH}(\textit{Ext}(\Gamma ({\mathcal W},A))) \subseteq \Gamma ({\mathcal W},{\{\beta \}})$ because $\Theta ({\mathcal W},A) \subseteq \textit{CH}(\textit{Ext}(\Gamma ({\mathcal W},A))) \subseteq \Gamma ({\mathcal W},A)$, by Auxiliary Lemma 12. Because, by Auxiliary Lemma 10, $\Gamma ({\mathcal W},{\{\beta \}})$ is convex, $\textit{CH}(\textit{Ext}(\Gamma ({\mathcal W},A))) \subseteq \Gamma ({\mathcal W},{\{\beta \}})$ holds if and only if $\textit{Ext}(\Gamma ({\mathcal W},A)) \subseteq \Gamma ({\mathcal W},{\{\beta \}})$, which, using Auxiliary Lemma 10(i), is if and only if for all $(w,r) \in \textit{Ext}(\Gamma ({\mathcal W},A))$, $f_w(\beta ) \le r$. Then $A\succcurlyeq _{\forall \forall \exists }^{{\mathcal W}} B$ holds if and only if for all $\beta \in B$, $A\succcurlyeq _{\forall \forall \exists }^{{\mathcal W}} {\{\beta \}}$, which holds if and only if for all $(w,r) \in \textit{Ext}(\Gamma ({\mathcal W},A))$ and for all $\beta \in B$ we have $f_w(\beta ) \le r$.

(iv): For real value *x*, let us define $\beta _x$ to be such that $f_w(\beta _x) = f_w(\beta ) - x$. Clearly, $\textit{SMR}_{\mathcal W}(A, {\{\beta _x\}}) = \textit{SMR}_{\mathcal W}(A, {\{\beta \}}) - x$. Thus, by Auxiliary Lemma 8(iii), $A\succcurlyeq _{\forall \forall \exists }^{{\mathcal W}} {\{\beta _x\}}$ if and only if $\textit{SMR}_{\mathcal W}(A, {\{\beta _x\}}) \le 0$, which is if and only if $\textit{SMR}_{\mathcal W}(A, {\{\beta \}}) \le x$.

So, $\textit{SMR}_{\mathcal W}(A, {\{\beta \}}) \le x$ if and only if $A\succcurlyeq _{\forall \forall \exists }^{{\mathcal W}} {\{\beta _x\}}$, which by part (iii) is if and only if for all $(w,r) \in \textit{Ext}(\Gamma ({\mathcal W},A))$ we have $f_w(\beta _x) \le r$. Now, $f_w(\beta _x) \le r$ $\iff$ $f_w(\beta ) -x \le r$ $\iff$ $f_w(\beta ) -r\le x$. Thus, $\textit{SMR}_{\mathcal W}(A, {\{\beta \}}) \le x$ if and only if $\max \{f_w(\beta ) - r \, : \,(w,r) \in$ $\textit{Ext}(\Gamma ({\mathcal W},A))\} \le x$. Since *x* is an arbitrary real number, this implies (iv). $\square$

### Results in Sect. 11

#### Proposition 17

Assume that for $w\in {I\!R}^{p}, \alpha \in {I\!R}^{p}$, $f_w(\alpha ) = w\cdot \hat{\alpha }$. Let ${\mathcal W}, {\mathcal W}' \subseteq {I\!R}^{p}$. If $\textit{CH}({\mathcal W}) = \textit{CH}({\mathcal W}')$ then $\succcurlyeq _{\mathcal W}$ $=$ $\succcurlyeq _{{\mathcal W}'}$. In particular, if ${\mathcal W}$ is a compact convex subset of ${I\!R}^{p}$ and ${\mathcal W}^0 = \textit{Ext}({\mathcal W})$ is the set of extreme points of ${\mathcal W}$ then $\succcurlyeq _{\mathcal W}$ $=$ $\succcurlyeq _{{\mathcal W}^0}$. Furthermore, binary relations $\succcurlyeq _{\forall \exists \forall }^{{\mathcal W}}$ and $\succcurlyeq _{\forall \exists \forall }^{{\mathcal W}^0}$ are equal; and $\succcurlyeq _{\exists \forall \forall }^{{\mathcal W}}$ equals $\succcurlyeq _{\exists \forall \forall }^{{\mathcal W}^0}$, and $A\succcurlyeq _{\exists \forall \forall }^{{\mathcal W}} B$ holds if and only if there exists $\alpha \in A$ such that for all $w\in {\mathcal W}^0$, $f_w(\alpha ) \ge \textit{Ut}_B(w)$.

#### Proof

Let ${\mathcal W}'' = \textit{CH}({\mathcal W}) = \textit{CH}({\mathcal W}')$. We will show that $\succcurlyeq _{\mathcal W}$ equals $\succcurlyeq _{{\mathcal W}''}$; the same proof will show $\succcurlyeq _{{\mathcal W}'}$ equals $\succcurlyeq _{{\mathcal W}''}$, and thus, $\succcurlyeq _{\mathcal W}$ $=$ $\succcurlyeq _{{\mathcal W}'}$. Since ${\mathcal W}'' \supseteq {\mathcal W}$, we have $\succcurlyeq _{\mathcal W}$ $\subseteq$ $\succcurlyeq _{{\mathcal W}''}$; to prove the converse, suppose that $\alpha \succcurlyeq _{\mathcal W}\beta$ and consider any $w\in {\mathcal W}''$. Then, by definition of convex hull, there exists $w_i \in {\mathcal W}$ and strictly positive reals $r_i$, for $i=1\ldots , k$, such that $\sum _{i=1}^k r_i = 1$ and $w= \sum _{i=1}^k r_i w_i$. Because $\alpha \succcurlyeq _{\mathcal W}\beta$, for each $i=1\ldots , k$, $w_i\cdot (\hat{\alpha }-\hat{\beta }) \ge 0$, and thus $w\cdot (\hat{\alpha }-\hat{\beta }) \ge 0$, showing that $w\cdot \hat{\alpha }\ge w\cdot \hat{\beta }$. This shows that $\alpha \succcurlyeq _{{\mathcal W}''} \beta$, and thus $\succcurlyeq _{\mathcal W}$ equals $\succcurlyeq _{{\mathcal W}''}$, and hence, $\succcurlyeq _{\mathcal W}$ $=$ $\succcurlyeq _{{\mathcal W}'}$.

Since ${\mathcal W}$ is compact and ${\mathcal W}^0$ is the set of extreme points of ${\mathcal W}$ we have $\textit{CH}({\mathcal W}^0) = {\mathcal W}$ and so $\succcurlyeq _{\mathcal W}$ $=$ $\succcurlyeq _{{\mathcal W}^0}$.

$A\succcurlyeq _{\forall \exists \forall }^{{\mathcal W}} B$ if and only if for all $\beta \in B$ there exists $\alpha \in A$ such that $\alpha \succcurlyeq _{\mathcal W}\beta$. And $A\succcurlyeq _{\exists \forall \forall }^{{\mathcal W}} B$ if and only if there exists $\alpha \in A$ such that for all $\beta \in B$, $\alpha \succcurlyeq _{\mathcal W}\beta$. The first part then implies $\succcurlyeq _{\forall \exists \forall }^{{\mathcal W}}$ and $\succcurlyeq _{\forall \exists \forall }^{{\mathcal W}^0}$ are equal; and $\succcurlyeq _{\exists \forall \forall }^{{\mathcal W}}$ equals $\succcurlyeq _{\exists \forall \forall }^{{\mathcal W}^0}$. And we also have that $A\succcurlyeq _{\exists \forall \forall }^{{\mathcal W}} B$ if and only if there exists $\alpha \in A$ such that for all $w\in {\mathcal W}^0$, $f_w(\alpha ) \ge \textit{Ut}_B(w)$. $\square$

#### Proposition 18

Assume that ${\mathcal W}$ is a compact subset of ${I\!R}^{p}$, and that for $w\in {I\!R}^{p}, \alpha \in {I\!R}^{p}$, $f_w(\alpha ) = w\cdot \hat{\alpha }$. Consider $A\in {\mathcal M}$ and $\beta \in {I\!R}^{p}$. Then $\textit{SMR}_{\mathcal W}(A, {\{\beta \}})$ is equal to the maximum value of *x* such that there exists $w\in {I\!R}^{p}$ satisfying the constraints (i) $w\in {\mathcal W}$; and (ii) for all $\alpha \in A$, $w\cdot (\hat{\beta }-\hat{\alpha }) \ge x$.

#### Proof

Because ${\mathcal W}$ is compact and $f_w(\gamma )$ is a continuous function of $w$ we have that $\textit{SMR}_{\mathcal W}(A, {\{\beta \}})$ equals $\max _{w\in {\mathcal W}} f_w(\beta ) - \textit{Ut}_A(w)$. Thus, for real-valued *x*, we have $\textit{SMR}_{\mathcal W}(A,$ ${\{\beta \}}) \ge x$ if and only if there exists $w\in {\mathcal W}$ such that for all $\alpha \in A$, $f_w(\beta ) - f_w(\alpha ) \ge x$, i.e., if and only if there exists $w\in {I\!R}^{p}$ satisfying the constraints (i) $w\in {\mathcal W}$; and (ii) for all $\alpha \in A$, $w\cdot (\hat{\beta }-\hat{\alpha }) \ge x$. Now, $x = \textit{SMR}_{\mathcal W}(A, {\{\beta \}})$ is the maximum value of *x* such that $\textit{SMR}_{\mathcal W}(A, {\{\beta \}}) \ge x$, i.e., such that there exists $w\in {I\!R}^{p}$ satisfying the constraints (i) $w\in {\mathcal W}$; and (ii) for all $\alpha \in A$, $w\cdot (\hat{\beta }-\hat{\alpha }) \ge x$. $\square$

#### Proposition 19

Assume that ${\mathcal W}$ is a convex subset of ${I\!R}^{p}$, and that for $w\in {I\!R}^{p}, \alpha \in {I\!R}^{p}$, $f_w(\alpha ) = w\cdot \hat{\alpha }$. Consider $A\in {\mathcal M}$, $\alpha \in A$, $w\in {\mathcal W}$.

(i) For any $\alpha \in A$, $\mathrm{Opt}_{\mathcal W}^A(\alpha )$ is a convex subset of ${\mathcal W}$.
(ii) If ${\mathcal W}$ is compact and $\alpha ,\beta \in A$, then $\mathrm{Opt}_{\mathcal W}^A(\alpha ) \subseteq \mathrm{Opt}_{\mathcal W}^A(\beta )$ $\iff$ $E_{\mathcal W}^A(\alpha ) \subseteq E_{\mathcal W}^A(\beta )$, i.e., $\textit{Ext}(\mathrm{Opt}_{\mathcal W}^A(\alpha )) \subseteq \textit{Ext}(\mathrm{Opt}_{\mathcal W}^A(\beta ))$.

#### Proof

(i) By Auxiliary Lemma 4, $\mathrm{Opt}_{\mathcal W}^A(\alpha ) = \bigcap _{\beta \in A} {\mathcal W}_{\alpha \ge \beta }$, where ${\mathcal W}_{\alpha \ge \beta } = \{w\in {\mathcal W}\, : \,$ $w\cdot (\hat{\alpha }-\hat{\beta }) \ge 0\}$, which is the intersection of a half-space in ${I\!R}^{p}$ with ${\mathcal W}$, and is thus convex, since ${\mathcal W}$ is convex. Any intersection of convex sets is convex, so $\mathrm{Opt}_{\mathcal W}^A(\alpha )$ is convex.

(ii) Assume ${\mathcal W}$ is compact. By a classical result, since $\mathrm{Opt}_{\mathcal W}^A(\alpha )$ is a compact convex subset of ${I\!R}^{p}$, it is the convex hull of its extreme points. Then $\mathrm{Opt}_{\mathcal W}^A(\alpha ) = \textit{CH}(\textit{Ext}(\mathrm{Opt}_{\mathcal W}^A(\alpha ))$, and $\mathrm{Opt}_{\mathcal W}^A(\beta ) = \textit{CH}(\textit{Ext}(\mathrm{Opt}_{\mathcal W}^A(\beta ))$. Thus, if $\textit{Ext}(\mathrm{Opt}_{\mathcal W}^A(\alpha )) \subseteq \textit{Ext}(\mathrm{Opt}_{\mathcal W}^A(\beta ))$ then $\textit{CH}(\textit{Ext}(\mathrm{Opt}_{\mathcal W}^A(\alpha )) \subseteq \textit{CH}(\textit{Ext}(\mathrm{Opt}_{\mathcal W}^A(\beta ))$, and hence, $\mathrm{Opt}_{\mathcal W}^A(\alpha ) \subseteq \mathrm{Opt}_{\mathcal W}^A(\beta )$.

For the converse, assume that $\mathrm{Opt}_{\mathcal W}^A(\alpha ) \subseteq \mathrm{Opt}_{\mathcal W}^A(\beta )$, and that $w$ is not an extreme point of $\mathrm{Opt}_{\mathcal W}^A(\beta )$. We will show that $w$ is not an extreme point of $\mathrm{Opt}_{\mathcal W}^A(\alpha )$. If $w\notin \mathrm{Opt}_{\mathcal W}^A(\alpha )$ then obviously $w$ is not an extreme point of $\mathrm{Opt}_{\mathcal W}^A(\alpha )$. Now consider the case of $w\in \mathrm{Opt}_{\mathcal W}^A(\alpha )$. We thus also have $w\in \mathrm{Opt}_{\mathcal W}^A(\beta )$. Since $w$ is not an extreme point of the convex set $\mathrm{Opt}_{\mathcal W}^A(\beta )$ there exists a line segment in $\mathrm{Opt}_{\mathcal W}^A(\beta )$ containing $w$, with $w$ not being an endpoint, i.e., there exists some $w_1, w_2 \in \mathrm{Opt}_{\mathcal W}^A(\beta )$ and $s \in (0,1)$ such that $w= sw_1 + (1-s)w_2$. Because $w_1, w_2 \in \mathrm{Opt}_{\mathcal W}^A(\beta )$, for $i=1,2$, $w_i\cdot \hat{\beta }\ge w_i\cdot \hat{\alpha }$, i.e., $w_i\cdot (\hat{\beta }-\hat{\alpha }) \ge 0$. Also, since $w\in \mathrm{Opt}_{\mathcal W}^A(\alpha )$ and $w\in \mathrm{Opt}_{\mathcal W}^A(\beta )$ we have $w\cdot (\hat{\beta }-\hat{\alpha }) =0$. Thus, $0 = w\cdot (\hat{\beta }-\hat{\alpha }) = sw_1\cdot (\hat{\beta }-\hat{\alpha }) + (1-s)w_2\cdot (\hat{\beta }-\hat{\alpha })$, and so, since both terms are non-negative, they are both zero: $w_1\cdot (\hat{\beta }-\hat{\alpha }) = w_2\cdot (\hat{\beta }-\hat{\alpha }) =0$. Because $w_1, w_2 \in \mathrm{Opt}_{\mathcal W}^A(\beta )$ ($\beta$ is optimal in $A$ with respect to both $w_1$ and $w_2$), this implies that $\alpha$ is optimal in $A$ with respect to both $w_1$ and $w_2$, i.e., $w_1, w_2 \in \mathrm{Opt}_{\mathcal W}^A(\alpha )$. Thus, there exists a line segment in the convex set $\mathrm{Opt}_{\mathcal W}^A(\alpha )$ containing $w$, where $w$ is not one of the endpoints of the line segment, so $w$ is not an extreme point of $\mathrm{Opt}_{\mathcal W}^A(\alpha )$. $\square$

#### Auxiliary Lemma 14

Assume that ${\mathcal W}$ is a convex subset of ${I\!R}^{p}$, and that for $w\in {I\!R}^{p}, \alpha \in {I\!R}^{p}$, $f_w(\alpha ) = w\cdot \hat{\alpha }$. Consider $A\in {\mathcal M}$, $\alpha \in A$, $w\in {\mathcal W}$. Let $I_\alpha = \{(w,r)\in {I\!R}^{p}\times {I\!R}\, : \,$ $r = w\cdot \hat{\alpha }\}$. For $K \subseteq {I\!R}^{p}\times {I\!R}$ we write $K^{\downarrow }$ for the projection of *K* to ${I\!R}^{p}$, i.e., $K^{\downarrow } = {\{w\in {I\!R}^{p}\, : \,(w,r) \in K\}}$.

(iii) $$\mathrm{Opt}_{\mathcal W}^A(\alpha ) = (\Gamma ({\mathcal W},A) \cap I_\alpha )^{\downarrow }$$.
(iv) $$\textit{Ext}((\Gamma ({\mathcal W},A) \cap I_\alpha )^{\downarrow }) = (\textit{Ext}(\Gamma ({\mathcal W},A) \cap I_\alpha ))^{\downarrow }$$.
(v) $$\textit{Ext}(\Gamma ({\mathcal W},A) \cap I_\alpha ) = \textit{Ext}(\Gamma ({\mathcal W},A)) \cap I_\alpha $$.

#### Proof

(i) $(w,r)\in (\Gamma ({\mathcal W},A) \cap I_\alpha )$ if and only if $r \ge \textit{Ut}_A(w)$ and $w\cdot \hat{\alpha }= r$, which is if and only if $r = w\cdot \hat{\alpha }= \textit{Ut}_A(w)$, which is if only if $w\in \mathrm{Opt}_{\mathcal W}^A(\alpha )$ and $r = w\cdot \hat{\alpha }$. Thus, $\mathrm{Opt}_{\mathcal W}^A(\alpha ) = (\Gamma ({\mathcal W},A) \cap I_\alpha )^{\downarrow }$.

(ii) Let us write $K = \Gamma ({\mathcal W},A) \cap I_\alpha$. First we prove, by contradiction, that $\textit{Ext}(K^{\downarrow }) \subseteq (\textit{Ext}(K))^{\downarrow }$. So, suppose that there exists some $w\in \textit{Ext}(K^{\downarrow }) {\setminus } (\textit{Ext}(K))^{\downarrow }$. Now, $w\in K^{\downarrow }$, so $(w,w\cdot \hat{\alpha }) \in K$. However, $w\notin (\textit{Ext}(K))^{\downarrow }$ implies that $(w,w\cdot \hat{\alpha }) \notin \textit{Ext}( K )$. Thus, there exists some $(w_1,r_1), (w_2,r_2) \in K$ and $s \in (0,1)$ such that $(w,w\cdot \hat{\alpha }) = s(w_1,r_1) + (1-s)(w_2,r_2)$. We must have $r_1 = w_1\cdot \hat{\alpha }$ and $r_2 = w_2\cdot \hat{\alpha }$. $w_1, w_2 \in (K)^{\downarrow }$ and $w= sw_1 + (1-s)w_2$, which contradicts $w\in \textit{Ext}(K^{\downarrow })$.

Conversely, we prove, by contradiction, that $\textit{Ext}(K^{\downarrow }) \supseteq (\textit{Ext}(K))^{\downarrow }$. Suppose there exists some $w\in (\textit{Ext}(K))^{\downarrow } \setminus \textit{Ext}(K^{\downarrow })$. Now, because $w\in (\textit{Ext}(K))^{\downarrow }$, there exists *r* such that $(w,r) \in \textit{Ext}(K)$. Then $(w,r) \in K$ implies $r = w\cdot \hat{\alpha }$, and thus, $(w,w\cdot \hat{\alpha }) \in \textit{Ext}(K)$. Since $w\in K^{\downarrow }$ and $w\notin \textit{Ext}(K^{\downarrow })$, there exists $w_1, w_2 \in K^{\downarrow }$ $s \in (0,1)$ such that $w= sw_1 + (1-s)w_2$. Since $w_1, w_2 \in K^{\downarrow }$, there exists $r_1$ and $r_2$ such that $(w_1,r_1), (w_2,r_2) \in K$. Then, $r_1 = w_1\cdot \hat{\alpha }$ and $r_2 = w_2\cdot \hat{\alpha }$, and so $(w,w\cdot \hat{\alpha }) = s(w_1,r_1) + (1-s)(w_2,r_2)$, which contradicts $(w,w\cdot \hat{\alpha }) \in \textit{Ext}(K)$.

(iii) For any convex subsets *P* and *Q* of some set we have $\textit{Ext}(P) \cap Q \subseteq \textit{Ext}(P \cap Q)$. To show this, we proceed with proof by contradiction: suppose that $x \in \textit{Ext}(P) \cap Q$ and $x\notin \textit{Ext}(P \cap Q)$. Since $x\in P \cap Q$ but is not in $\textit{Ext}(P \cap Q)$, there exists a line segment in $P \cap Q$ that contains *x* as an internal point. But this line segment is also in *P*, contradicting $x \in \textit{Ext}(P)$.

The above result shows that $\textit{Ext}(\Gamma ({\mathcal W},A) \cap I_\alpha ) \supseteq \textit{Ext}(\Gamma ({\mathcal W},A)) \cap I_\alpha$. To prove the converse, we proceed by contradiction and assume that there exists some element $(w,r) \in \textit{Ext}(\Gamma ({\mathcal W},A) \cap I_\alpha )$ with $(w,r) \notin \textit{Ext}(\Gamma ({\mathcal W},A)) \cap I_\alpha$. Since $(w,r) \in \Gamma ({\mathcal W},A) \setminus \textit{Ext}(\Gamma ({\mathcal W},A))$ there exist some $(w_1,r_1), (w_2,r_2) \in \Gamma ({\mathcal W},A)$ and $s \in (0,1)$ such that $(w,r) = s(w_1,r_1) + (1-s)(w_2,r_2)$. Now, $(w,r) \in I_\alpha$ implies that $r = w\cdot \hat{\alpha }$; and $(w_1,r_1), (w_2,r_2) \in \Gamma ({\mathcal W},A)$ implies that $r_1 \ge \textit{Ut}_A(w_1)\ge w_1\cdot \hat{\alpha }$ and $r_2 \ge \textit{Ut}_A(w_2) \ge w_2\cdot \hat{\alpha }$. We have $r = s r_1 + (1-s) r_2$ and so $0 = r-(w\cdot \hat{\alpha }) = s (r_1 - w_1\cdot \hat{\alpha }) + (1-s) (r_2 -w_2\cdot \hat{\alpha })$. Since the two parts of the right-hand-side are non-negative, they must be zero, showing that $r_1 = w_1\cdot \hat{\alpha }$ and $r_2 =w_2\cdot \hat{\alpha }$. This implies that $(w_1,r_1), (w_2,r_2) \in \Gamma ({\mathcal W},A) \cap I_\alpha$, which contradicts $(w,r) \in \textit{Ext}(\Gamma ({\mathcal W},A) \cap I_\alpha )$. $\square$

#### Proposition 20

Assume that ${\mathcal W}$ is a convex subset of ${I\!R}^{p}$, and that for $w\in {I\!R}^{p}, \alpha \in {I\!R}^{p}$, $f_w(\alpha ) = w\cdot \hat{\alpha }$. Consider $A\in {\mathcal M}$, $w\in {\mathcal W}$, and $\alpha ,\beta \in A$.

(i) $$E_{\mathcal W}^A(\alpha ) = {\{w\in {I\!R}^{p}\, : \,(w,w\cdot \hat{\alpha }) \in \textit{Ext}(\Gamma ({\mathcal W},A)) \}}$$.
(ii) If ${\mathcal W}$ is compact then $\textit{dim}(\mathrm{Opt}_{\mathcal W}^A(\alpha )) < |E_{\mathcal W}^A(\alpha )|$.

#### Proof

(i): using parts (i), (ii) and (iii) of Auxiliary Lemma 14, $\textit{Ext}(\mathrm{Opt}_{\mathcal W}^A(\alpha )) =$ $(\textit{Ext}(\Gamma ({\mathcal W},$ $A)) \cap I_\alpha )^{\downarrow } = {\{w\in {I\!R}^{p}\, : \,(w,w\cdot \hat{\alpha }) \in \textit{Ext}(\Gamma ({\mathcal W},A)) \}}$.

(ii): Since ${\mathcal W}$ is compact, $\mathrm{Opt}_{\mathcal W}^A(\alpha )$ is compact since it is a closed subset of compact set ${\mathcal W}$, and so, $\mathrm{Opt}_{\mathcal W}^A(\alpha ) = \textit{CH}(E_{\mathcal W}^A(\alpha ))$. Let $e_0$ be an arbitrary element of $E_{\mathcal W}^A(\alpha )$. The dimension of $\textit{CH}(E_{\mathcal W}^A(\alpha ))$ is the same as the dimension of the affine closure of $E_{\mathcal W}^A(\alpha )$, which equals the dimension of the vector space spanned by $X = {\{e - e_0 \, : \,e \in E_{\mathcal W}^A(\alpha ) \setminus {\{e_0\}}\}}$, which is at most $|X| = |E_{\mathcal W}^A(\alpha ))| -1$. This implies that $\textit{dim}(\mathrm{Opt}_{\mathcal W}^A(\alpha )) < |E_{\mathcal W}^A(\alpha ))|$. $\square$

#### Auxiliary Lemma 15

Assume that ${\mathcal W}$ is a convex subset of ${I\!R}^{p}$, and ${\mathcal W}'$ is a convex subset of ${\mathcal W}$. Let $int ({\mathcal W}')$ be the interior of ${\mathcal W}'$ with respect to the topology on ${\mathcal W}$. The following are equivalent.

- $int ({\mathcal W}') \not =\emptyset$, i.e., there exists an open set *B* in ${I\!R}^{p}$ such that $B \cap {\mathcal W}\subseteq {\mathcal W}'$;
- ${\mathcal W}'$ has the same dimension as ${\mathcal W}$.

#### Proof

From the definition of interior we have $int ({\mathcal W}') \not =\emptyset$ if and and only if ${\mathcal W}'$ contains an open set $B'$ in the topology on ${\mathcal W}$ induced from ${I\!R}^{p}$, which is if and only if there exists an open set *B* in ${I\!R}^{p}$ such that $B \cap {\mathcal W}\subseteq {\mathcal W}'$.

We write $$ aff U$$ for the affine hull of convex set *U*. Since $${\mathcal W}' \subseteq {\mathcal W}$$, $${\mathcal W}'$$ and $${\mathcal W}$$ have the same dimension if and only if $$ aff {\mathcal W}' = aff {\mathcal W}$$, which is if and only if the interior of $${\mathcal W}'$$ is the same as the relative interior of $${\mathcal W}'$$. The relative interior of a convex set in $${I\!R}^{p}$$ is non-empty by [48], Theorem 6.2, page 45, and so, if (b) then (a) the interior of $${\mathcal W}'$$ is non-empty.

If (a) there exists an open set *B* in ${I\!R}^{p}$ such that $B \cap {\mathcal W}\subseteq {\mathcal W}'$ then $aff B \cap {\mathcal W}= aff {\mathcal W}$ and so $aff {\mathcal W}' = aff {\mathcal W}$, which implies (b) that ${\mathcal W}'$ and ${\mathcal W}$ have the same dimension. $\square$

#### Proposition 21

Assume that ${\mathcal W}$ is a compact convex subset of ${I\!R}^{p}$, and that for $w\in {I\!R}^{p}, \alpha \in {I\!R}^{p}$, $f_w(\alpha ) = w\cdot \hat{\alpha }$. Consider $A\in {\mathcal M}$ and $\alpha ,\beta \in A$.

(i) $\beta$ $\mathrm{Opt}_{\mathcal W}^A$-dominates $\alpha$ if and only if $E_{\mathcal W}^A(\alpha ) \subsetneqq E_{\mathcal W}^A(\beta )$,
(ii) If $E_{\mathcal W}^A(\alpha ) =E_{\mathcal W}^A(\beta )$ and $\alpha \not \equiv _{\mathcal W}\beta$ then both $\alpha$ and $\beta$ are $\mathrm{Opt}_{\mathcal W}^A$-dominated.
(iii) If $|E_{\mathcal W}^A(\alpha )| \le \textit{dim}({\mathcal W})$ then $\alpha$ is $\mathrm{Opt}_{\mathcal W}^A$-dominated.

#### Proof

By definition, $\beta$ $\mathrm{Opt}_{\mathcal W}^A$-dominates $\alpha$ if and only if $\mathrm{Opt}_{\mathcal W}^A(\alpha ) \subsetneqq \mathrm{Opt}_{\mathcal W}^A(\beta )$, which, using Proposition 19(ii), is if and only if $E_{\mathcal W}^A(\alpha ) \subsetneqq E_{\mathcal W}^A(\beta )$.

(ii): Assume $\alpha \not \equiv _{\mathcal W}\beta$ and $E_{\mathcal W}^A(\alpha ) =E_{\mathcal W}^A(\beta )$, and so, by Proposition 19(ii), $\mathrm{Opt}_{\mathcal W}^A(\alpha ) = \mathrm{Opt}_{\mathcal W}^A(\beta )$. This implies that neither $\alpha$ nor $\beta$ is strictly optimal, i.e., $\alpha ,\beta \notin \mathrm{PSO}_{\mathcal W}(A)$, so $\alpha ,\beta \notin \mathrm{MPO}_{\mathcal W}(A)$ (because and $\mathrm{PSO}_{\mathcal W}(A)$ equals $\mathrm{MPO}_{\mathcal W}(A)$, by Corollary 3). Thus, both $\alpha$ and $\beta$ are $\mathrm{Opt}_{\mathcal W}^A$-dominated, by Lemma 7.

(iii): If $|E_{\mathcal W}^A(\alpha )| \le \textit{dim}({\mathcal W})$ then, by Proposition 20(ii), $\textit{dim}(\mathrm{Opt}_{\mathcal W}^A(\alpha )) < \textit{dim}({\mathcal W})$. Then, using Auxiliary Lemma 15, $\mathrm{Opt}_{\mathcal W}^A(\alpha )$ contains no open set in ${\mathcal W}$, which implies that $\alpha \notin \mathrm{PSO}_{\mathcal W}(A)$, by Proposition 11. Thus, by Corollary 3, $\alpha \notin \mathrm{MPO}_{\mathcal W}(A)$, and so, by Lemma 7, $\alpha$ is $\mathrm{Opt}_{\mathcal W}^A$-dominated. $\square$

### Results in Sect. 12

#### Proposition 22

Assume that for $w\in {I\!R}^{p}, \alpha \in {I\!R}^{p}$, $f_w(\alpha ) = w\cdot \hat{\alpha }$. Let ${\mathcal W}$ be a compact and convex subset of ${I\!R}^{p}$ and let ${\mathcal W}^0 = \textit{Ext}({\mathcal W})$ be the set of extreme points of ${\mathcal W}$. Then

(i) $$A\succcurlyeq _{\exists \forall \forall }^{{\mathcal W}^0} B\ \Rightarrow \ A\succcurlyeq _{\forall \forall \exists }^{{\mathcal W}} B\ \Rightarrow \ A\succcurlyeq _{\forall \forall \exists }^{{\mathcal W}^0} B$$.
(ii) $A\succcurlyeq _{\exists \forall \forall }^{{\mathcal W}^0} B$ holds iff there exists $\alpha \in A$ such that for all $w\in {\mathcal W}^0$, $f_w(\alpha ) \ge \textit{Ut}_B(w)$.
(iii) $A\succcurlyeq _{\forall \forall \exists }^{{\mathcal W}^0} B$ holds iff for each $w\in {\mathcal W}^0$ there exists $\alpha \in A$ such that $f_w(\alpha ) \ge \textit{Ut}_B(w)$.

#### Proof

(i): The first implication holds because $A\succcurlyeq _{\exists \forall \forall }^{{\mathcal W}^0} B$ $\iff$ $A\succcurlyeq _{\exists \forall \forall }^{{\mathcal W}} B$ $\Rightarrow$ $A\succcurlyeq _{\forall \forall \exists }^{{\mathcal W}} B$ by Proposition 17 and nestedness (Proposition 1). The second implication holds by monotonicity with respect to ${\mathcal W}$ (Proposition 4).

(ii) is an immediate consequence of Proposition 3(iii).

(iii): By Proposition 3(i), $A\succcurlyeq _{\forall \forall \exists }^{{\mathcal W}^0} B$ holds and only if for each $w\in {\mathcal W}^0$, $\textit{Ut}_A(w) \ge \textit{Ut}_B(w)$, that is, if and only if, for each $w\in {\mathcal W}^0$ there exists $\alpha \in A$ such that $f_w(\alpha ) \ge \textit{Ut}_B(w)$. $\square$

#### Lemma 9

(i) $A\succcurlyeq _{\forall \forall \exists }^{{\mathcal W}} B$ if and only if $\mathrm{UD}_{\mathcal W}(A) \succcurlyeq _{\forall \forall \exists }^{{\mathcal W}} \mathrm{UD}_{\mathcal W}(B)$.
(ii) Suppose that $A\succcurlyeq _{\forall \exists \forall }^{{\mathcal W}} C$. Then $A\succcurlyeq _{\forall \forall \exists }^{{\mathcal W}} B$ if and only if $A\succcurlyeq _{\forall \forall \exists }^{{\mathcal W}} B\setminus C$.

#### Proof

Part (i) is immediate from Proposition 5. Regarding part (ii), the $\Rightarrow$ part follows immediately from the definition; $\Leftarrow$: $A\succcurlyeq _{\forall \exists \forall }^{{\mathcal W}} C$ implies $A\succcurlyeq _{\forall \forall \exists }^{{\mathcal W}} C$; the latter together with $A\succcurlyeq _{\forall \forall \exists }^{{\mathcal W}} B\setminus C$ implies $A\succcurlyeq _{\forall \forall \exists }^{{\mathcal W}} B$ from the definition of $\succcurlyeq _{\forall \forall \exists }^{{\mathcal W}}$. $\square$

## B Random problem generator

This appendix includes a description of the random problem generator that is used for the experiments in Sect. 13.

Let $j\in \{1,\dots , J\}$ be the index of each set of alternatives $A_j$ that we are going to generate. Consider now any *j*. For each criterion $i\in \{1,\dots , {p}\}$ we pick random parameters $\mu _j(i)$ and $\delta _j(i)$, and each of the *n* elements $\alpha$ of $A_j$ is picked independently as follows: for each criterion *i*, choose value $\alpha (i)$ uniformly in range $[\mu _j(i) - \delta _j(i), \mu _j(i) + \delta _j(i)]$. Choosing random parameters $\mu _j(i)$ and $\delta _j(i)$ for $A_j$: first we randomly pick $\mu _j$ uniformly in range $[-\mu , \mu ]$, and $\theta _j$ is chosen uniformly in range $[0, 2\theta ]$, and $\delta _j$ is chosen uniformly in range $[0, 2\delta ]$. Then, for each criterion *i*, $\mu _j(i)$ is chosen uniformly in range $[\mu _j - \theta _j, \mu _j + \theta _j]$ and $\delta _j(i)$ is chosen uniformly in range $[0, 2\delta _j]$. The pseudocode is shown in Algorithm 1. The generated sets will contain only undominated elements if and only if the Boolean input *u* is True, and integer numbers if the Boolean input *int* is True, rational otherwise.

Note that if $\theta$ and $\delta$ are both very small, then each $\theta _j$ and $\delta _j$ will be very small, so each $\mu _j(i)$ will be very close to $\mu _j$. Generating two sets $A$ and $B$ (i.e. $J=2$), this would lead to a case in which we will tend to almost always get that $A\succcurlyeq _{\exists \forall \forall }^{{\mathcal W}} B$ or $B\succcurlyeq _{\exists \forall \forall }^{{\mathcal W}} A$ (since the elements of e.g., $A$ will be very similar to each other). This will also tend (to a somewhat lesser extent) to be the case if just $\theta$ is very small. On the other hand, if $\theta$ and $\delta$ are relatively large, then we will tend to get less dominance. We tried different values of the input parameters, obtaining the lowest rate of dominance with $\mu =10$, $\theta =50$ and $\delta =60$.

In our random problem generator we also randomly generate $\rho$ user preferences of the form $aw_i+bw_j\ge cw_k$ meaning that the user prefers *a* units of $w_i$ and *b* units of $w_j$ to *c* units of $w_k$. We ensure the consistency of such constraints by first randomly picking a normalised vector $w$, and only including constraints that are consistent with this vector (e.g., if a constraint randomly generated that is not consistent with this, then we change the sign of the constraint to make it consistent). The values *a*, *b* and *c* are rational numbers.

## Footnotes

1 One can define $\textit{Filter}_\sigma (A; \gg _{\forall \forall \exists }^{{\mathcal W}})$ analogously, using the strong strict version of the dominance relation (see the remark after Proposition 3) with the result being $\mathrm{PO}_{\mathcal W}(A)$, irrespective of $\sigma$, and without requiring any conditions on $A$; see Algorithm 1 and Theorem 1 in [10].

2 In contrast, in generating $\mathrm{PSO}_{\mathcal W}(B)$ we are finding a minimal subset $A$ of $B$ such that $\textit{SMR}_{\mathcal W}(A, B) = 0$ (under appropriate assumptions, such as those for Theorem 3).

3 Learning an inconsistency could in theory be useful information, allowing the potential of updating the model in some way to restore consistency; however, this would probably have a heavy computational cost, and in a practical application, one will want to avoid the incremental elicitation procedure breaking down.

4 In fact, if $\mathrm{Opt}_{\mathcal W}^A(\alpha ) \subsetneqq \mathrm{Opt}_{\mathcal W}^A(\beta )$ then $\mathrm{Opt}_{\mathcal W}^A(\beta )$ is of higher dimension than $\mathrm{Opt}_{\mathcal W}^A(\alpha )$, and $T \cap \mathrm{Opt}_{\mathcal W}^A(\beta ) = \mathrm{Opt}_{\mathcal W}^A(\alpha )$, where *T* is the affine hull of $\mathrm{Opt}_{\mathcal W}^A(\alpha )$ (the vector subspace of ${I\!R}^{p}$ generated by $\mathrm{Opt}_{\mathcal W}^A(\alpha )$). This is because for any $w\in \mathrm{Opt}_{\mathcal W}^A(\alpha )$, we have $w\in \mathrm{Opt}_{\mathcal W}^A(\beta )$, which implies that $w\cdot (\hat{\beta }-\hat{\alpha }) =0$. This then implies that $w'\cdot (\hat{\beta }-\hat{\alpha }) =0$ for any $w' \in T$, and so $T \cap \mathrm{Opt}_{\mathcal W}^A(\beta ) = \mathrm{Opt}_{\mathcal W}^A(\alpha )$.
